# Design, Synthesis and Antimicrobial Properties of New Tetracyclic Quinobenzothiazine Derivatives

**DOI:** 10.3390/ijms232315078

**Published:** 2022-12-01

**Authors:** Ewa Kisiel-Nawrot, Dominika Pindjakova, Malgorzata Latocha, Andrzej Bak, Violetta Kozik, Kinga Suwinska, Aleksander Sochanik, Alois Cizek, Josef Jampilek, Andrzej Zięba

**Affiliations:** 1 Department of Organic Chemistry, Faculty of Pharmaceutical Sciences in Sosnowiec, Medical University of Silesia, Jagiellońska 4, 41-200 Sosnowiec, Poland; 2 Department of Analytical Chemistry, Faculty of Natural Sciences, Comenius University, Ilkovicova 6, 842 15 Bratislava, Slovakia; 3 Department of Cell Biology, Faculty of Pharmaceutical Sciences in Sosnowiec, Medical University of Silesia, Jedności 9, 41-200 Sosnowiec, Poland; 4 Institute of Chemistry, University of Silesia, Szkolna 9, 40-007 Katowice, Poland; 5 Faculty of Mathematics and Natural Sciences, Cardinal Stefan Wyszyński University, K. Woycickiego 1/3, 01-938 Warszawa, Poland; 6 Center for Translational Research and Molecular Biology of Cancer, Maria Skłodowska-Curie National Research Institute of Oncology, Wybrzeże AK 15, 44-101 Gliwice, Poland; 7 Department of Infectious Diseases and Microbiology, Faculty of Veterinary Medicine, University of Veterinary Sciences Brno, Palackeho 1946/1, 612 42 Brno, Czech Republic; 8 Department of Chemical Biology, Faculty of Science, Palacky University Olomouc, Slechtitelu 27, 783 71 Olomouc, Czech Republic

**Keywords:** phenothiazine, azaphenothiazines, antibacterial activity, cytotoxicity, descriptor-based similarity analysis

## Abstract

A new method for modifying the structure of tetracyclic quinobenzothiazinium derivatives has been developed, allowing introduction of various substituents at different positions of the benzene ring. The method consists of reacting appropriate aniline derivatives with 5,12-(dimethyl)thioquinantrenediinium bis-chloride. A series of new quinobenzothiazine derivatives was obtained with propyl, allyl, propargyl and benzyl substituents in 9, 10 and 11 positions, respectively. The structure of the obtained compounds was analyzed by ^1^H and ^13^C NMR (HSQC, HMBC) and X-ray analysis. All the compounds were tested against reference strains *Staphylococcus aureus* ATCC 29213 and *Enterococcus faecalis* ATCC 29212, and representatives of multidrug-resistant clinical isolates of methicillin-resistant *S. aureus* (MRSA) and vancomycin-resistant *E. faecalis* (VRE). In addition, all the compounds were evaluated in vitro against *Mycobacterium smegmatis* ATCC 700084 and *M. marinum* CAMP 5644. 9-Benzyloxy-5-methyl-12*H*-quino [3,4-*b*][1,4]benzothiazinium chloride (**6j**), 9-propoxy-5-methyl-12*H*-quino[3,4-*b*][1,4]benzothiazinium chloride (**6a**) and 9-allyloxy-5-methyl-12*H*-quino[3,4-*b*][1,4]benzothiazinium chloride (**6d**) demonstrated high activity against the entire tested microbial spectrum. The activities of the compounds were comparable with oxacillin, tetracycline and ciprofloxacinagainst staphylococcal strains and with rifampicin against both mycobacterial strains. Compound **6j** had a significant effect on the inhibition of bacterial respiration as demonstrated by the MTT assay. The compounds showed not only bacteriostatic activity, but also bactericidal activity. Preliminary in vitro cytotoxicity screening of the compounds performed using normal human dermal fibroblasts (NHDF) proved that the tested compounds showed an insignificant cytotoxic effect on human cells (IC_50_ > 37 µM), making these compounds interesting for further investigation. Moreover, the intermolecular similarity of novel compounds was analyzed in the multidimensional space (mDS) of the structure/property-related in silico descriptors by means of principal component analysis (PCA) and hierarchical clustering analysis (HCA), respectively. The distance-oriented structure/property distribution was related with the experimental lipophilic data.

## 1. Introduction

Phenothiazine is a tricyclic heterocyclic compound with a 1,4-thiazine system. This compound was first obtained by Bernthsen in 1883 and has been an object of interest for researchers since, because the compound exhibits very interesting chemical and biological properties [1]. Phenothiazines, substituted at position 10 with alkylaminoalkyl groups, constitute the first group of effective neuroleptics. They have also been used as antiemetic, antihistamine, antipruritic, analgesic and anthelmintic drugs [2,3]. Increasingly, the design and production of new drugs is based on modifications of the basic structural fragments of known and already used drugs. The phenothiazine system is an example of how slight structural changes can affect the strength and the direction of drugs’ interaction. Thus, the phenothiazine skeleton has become a platform for the design and synthesis of biologically active compounds [4,5,6,7]. Some sources claim that over 6000 new phenothiazine derivatives have been reported to date [8]. Recent reports concern, inter alia, antitumor, antibacterial, antiviral, anti-inflammatory and antioxidant activities, reversal of multi-drug resistance and potential treatment of Alzheimer’s and Creutzfeldt-Jakob disease [9,10,11,12,13,14]. The influence of various types of pharmacophore groups on the direction of biological interaction has been extensively analysed. However, there are no reports on the influence of the location of these groups in the phenothiazine or azaphenothiazine system on the interaction of these compounds. The phenothiazine system is usually obtained by four basic methods: (i) thionation of diphenylamines with elemental sulphide, (ii) cyclization of diphenyl sulphides proceeding directly as Ullmann cyclization or with Smiles rearrangement, (iii) reactions of *ortho*-aminobenzenethiols with *ortho*-disubstituted benzenes, (iv) addition of the benzene ring to the benzene moiety 1,4-thiazines [15]. The substitution of one or both of the benzene rings with heteroaromatic azine rings leads to the production of nitrogen analogues of phenothiazines (azaphenothiazines). The structural modifications of phenothiazines and azaphenothiazines described so far mainly involved the introduction of various types of substituents to the thiazine nitrogen atom. In fact, the introduction of substituents to the carbon atoms of the benzene or azine rings is difficult; therefore, there are few published papers on the synthesis of these types of derivatives [16,17,18]. Halogen atoms or small functional groups were most often introduced into benzene rings. So far, the influence of various types of substituents at the thiazine nitrogen atom on the biological activity of phenothiazine derivatives and their nitrogen analogues has been studied. The effect that the nature of the substituents and their position on the benzene rings will exert on the biological activity of phenothiazine derivatives is of particular interest.

As a matter of fact, similarity-based property profiling is still regarded as the core of many SAR-guided procedures which assume that small modifications of molecular structures correspond to small variations of biological activities, and vice versa [19].Despite some shortcomings, the distance-related similarity scrutiny of structurally alike compounds is common practice that contributes noticeably to quantitative and/or qualitative on-target (receptor-dependent) and off-target (receptor-independent) SAR mapping [20]. In practice, chemical composition (topology and/or topography) can be structurally coded by calculated multi-dimensional (mD) descriptors and/or represented by experimental property data. Accordingly, SAR-related exploration of the descriptor-based feature/structural chemical space (CS) seems crucial to quantitative potency modelling and ADMET-tailored property prediction or production. It is expected that in silico mapping of molecular descriptors to targeted functionalities can support the synthetic efforts at the decision-making phases of hit→lead→seed→drug design [21].

In this work, we present a new method of synthesis of phenothiazine derivatives that allows the introduction of substituents at different positions in the phenothiazine system. We analyse the dependence of the antibacterial/antimycobacterial and cytotoxic properties of the obtained quinobenzothiazine derivatives on both the nature of the introduced substituents and their position in the quinobenzothiazine system. Following the common practice, the intermolecular similarity of novel derivatives was estimated in the multidimensional space (mDS) of the structure/property-related in silico descriptors using principal component analysis (PCA) and hierarchical clustering analysis (HCA), respectively. Moreover, the distance-oriented property distribution for new series of compounds was correlated with the experimental TLC lipophilic data. Finally, the antibacterial profile of newly synthesised molecules was investigated.

## 2. Results and Discussion

### 2.1. Chemistry—Design and Synthesis

In our previous reports, we described the method for the synthesis of tetracylic quinobenzothiazinium derivatives, in which the intermediate products are 4-aminoquinoline betaine systems containing a negatively charged sulphur atom in the 3-quinoline position. In the presence of hydrogen chloride and oxidant (atmospheric oxygen) these compounds undergo cyclization, consisting of oxidative nucleophilic substitution of the hydrogen atom with a thialate sulphur atom [22,23]. Using this method, we obtained a number of tetracyclic azaphenothiazine and diazaphenothiazine derivatives containing simple substituents in the benzene ring, such as: halogen atoms, amino, hydroxyl or methoxy groups. We observed a significant influence of these substituents on the antibacterial and antitumor activity of this group of compounds [24,25]. As we reported earlier, there are few examples of modifications of the phenothiazine system structure consisting of introducing substituents to benzene rings. We undertook such attempts earlier by functionalising the hydroxyl group in the quinobenzothiazinium system [16]. Using this method, we introduced, among other things, aminoalkyl groups at position 10. However, such reactions could not be performed at other positions of the quinobenzothiazinium system. Therefore, in this study we present a new method of introducing substituents at various positions of the quinobenzothiazinium system by modifying aniline derivatives, the substrates in reactions leading to this group of compounds.

**Scheme 1:**
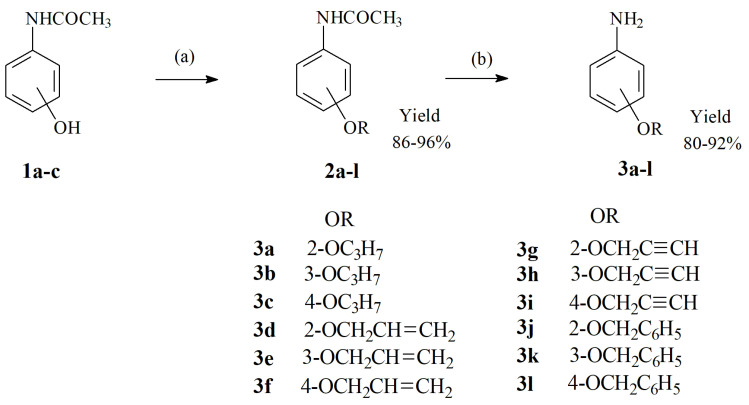
Preparation of the alkoxy aniline derivatives **2a**–**l** and **3a**–**l**. Reagents and conditions: (**a**) DMF, NaH, RBr, room temp. (**b**) 36% HCl aq, EtOH, 60 °C.

For this purpose, we performed alkylation reactions of isomeric hydroxyacetamides **1a**–**c** with propyl, allyl, propagyl and benzyl bromides. The reaction was carried out in anhydrous DMF in the presence of a stoichiometric amount of sodium hydride. The corresponding alkoxy acetanilide derivatives **2a**–**l** were obtained in good yields. The crude compounds **2** were identified immediately after receipt by ^1^H, ^13^C NMR and HR-MS and processed further without further purification; therefore, we do not report their melting points. Compounds **2a**–**l** were converted to the corresponding alkoxy aniline derivatives **3a**–**l** by hydrolysis in a mixture of hydrochloric acid and ethanol as shown in Scheme 1. The structure of compounds **3** was confirmed by ^1^H and ^13^C NMR spectroscopy as well as HR-MS spectrometry.

Next we conducted the reaction of 5,12-(dimethyl)thiquinanthrenediinium bis-chloride **4** with the corresponding aniline derivatives **3a**–**l**. The reactions were carried out in anhydrous pyridine using a 2.5-fold molar excess of compounds **3** with respect to the bis-chloride **4**.

**Scheme 2:**
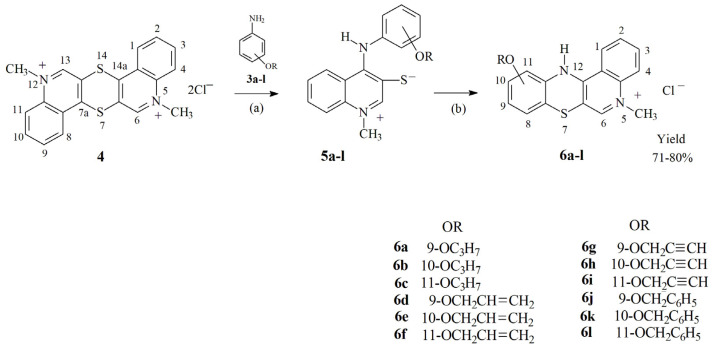
Preparation of alkoxy quinobenzothiazinium derivatives **6a**–**l**. Reagents and conditions: (**a**) Procedure A: pyridine, 80 °C; Procedure B: pyridine, 20 °C; (**b**) pyridine, HCl, O_2_.

As in the case of the previously described reactions of bis-chloride **4** with aniline derivatives, the reactions with compounds **3** proceeded in two stages. First, there was a nucleophilic attack of the amine in positions 7a and 14a, which led to the cleavage of the dithiine ring and the formation of two intermediate molecules with the structure of 1-methyl-4-(arylamino)quinolinio-3-thiolates **5a**–**l**. In the second step, compounds **5a**–**l** were cyclised to quinobenzothiazinium derivatives **6a**–**l** (see Scheme 2). Cyclization of the intermediates **5** proceeds as a nucleophilic oxidative substitution of a hydrogen atom by a thiolate sulphur atom. Such a reaction requires the presence of atmospheric oxygen as an oxidant in the reaction medium.

**Scheme 3:**
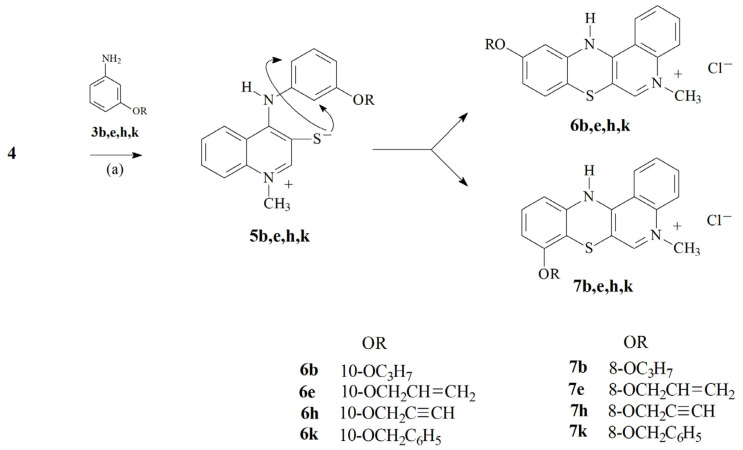
Influence of the structure of intermediate products **5** on the direction of the cyclization reaction. (**a**) Procedure A: pyridine, 80 °C; Procedure B: pyridine, 20 °C.

In the case of quinolinio-3-thiolanes formed by the reaction of bis-salt **4** with 2- or 4-alkoxy aniline derivatives, the cyclization reaction proceeded selectively to the corresponding compounds **6**. In the case of quinolinio-3-thiolanes formed by the reaction of bis-chloride **4** of 3-alkoxy aniline derivatives, the cyclization can take place at the 2 or 4 position of the arylamino ring of the compounds **5** (see Scheme 3). When the reactions were carried out at 80 °C, it resulted in a mixture of products **6** and **7** in a molar ratio close to 3:1. The composition of the mixtures was determined on the basis of signal integration in the ^1^H NMR spectra. These compounds have very similar physicochemical properties and it was not possible to separate them by chromatographic methods and recrystallization. When the reaction of bis-chloride **4** with 3-alkoxy aniline derivatives was carried out at 20 °C, it mainly led to compounds **6** having alkoxy substituents at the 10 position of the quinobenzothiazinium system. Only traces of compounds **7** were found in the crude products. Pure compounds **6** were obtained from the crude products. It was not possible to isolate and characterise compounds **7** in pure form from post-reaction mixtures.

### 2.2. Structural Analysis

The structure of all compounds obtained was confirmed by ^1^H and ^13^C NMR spectroscopy using two-dimensional HSQC and HMBC techniques. Additionally, the structure was confirmed by the HR-MS method using the ESI ionization method. For all the compounds, the experimentally determined molecular weights differed from the theoretically calculated values only in the third decimal place, which fully confirms the elemental composition of these compounds and their structure.

**Figure 1:**
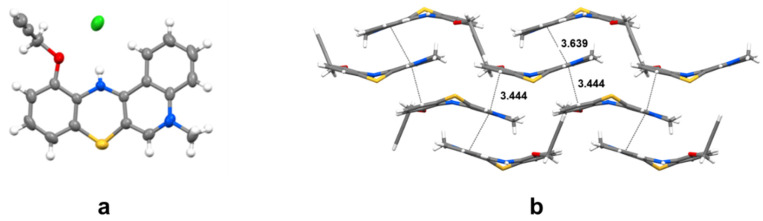
(**a**) Molecular structure of 11-propargyloxy-5-methyl-12*H*-quinolino[3,4-*b*][1,4]benzo thiazinium chloride (**6i**) (displacement ellipsoids for non-hydrogen atoms are drawn at a 50% probability level). (**b**) π–π stacking interactions in the crystal.

X-ray analysis not only confirmed the structure of the final products determined by spectroscopic methods, but also showed their spatial structure in the solid phase. By crystallization from ethanol, it was possible to obtain a single crystal of the derivative **6i**. The X-ray structure of the derivative **6i** is shown in Figure 1. The molecule of 11-propargyloxy-5-methyl-12*H*-quino[3,4-*b*][1,4]benzothiazinium chloride **6i** is bent along the axis defined by the nitrogen and sulphur atom of the thiazine ring under the angle of 146.5°. The angle formed by the C6a-S7-C7a atoms in the thiazine ring is 97.9°, while that between the C11a-N12-C12a atoms is 122.3°. Most of the structural parameters of the molecule are similar (or correspond well) to the parameters of the previously reported unsubstituted tetracyclic structure of 5-methyl-12*H*-quino[3,4-*b*][1,4]benzothiazinium chloride [22].

Each molecule interacts with adjacent molecules with three π–π stacking interactions, shown in Figure 1 with grey broken lines.

### 2.3. Biological Screening

#### 2.3.1. In Vitro Antimicrobial Activity

Some of the previously obtained quinobenzothiazinium derivatives showed interesting antimicrobial properties [10]. All the investigated compounds were tested for in vitro antibacterial and antimycobacterial activity against a battery of microbial pathogens. The selection of the studied bacterial strains was adopted following the CLSI (National Committee for Clinical Laboratory Standards) international reference methodologies [26], i.e., standardization. For this purpose, universally sensitive collection strains from ATCC (*Staphylococcus aureus* ATCC 29213 and *Enterococcus faecalis* ATCC 29212) were selected. The second aspect of strain selection was the current state of occurrence of strains with an epidemiologically significant type of resistance, represented by clinical isolates of human and veterinary origin, i.e., different sequence types limited to human and animal populations, e.g., methicillin-resistant *Staphylococcus aureus* (MRSA) SA 3202, SA 630 and 63718 isolates carrying the *mecA* gene [27]. In the case of vancomycin-resistant *E. faecalis* (VRE) 342B, 368 and 725B isolates carrying the *vanA* gene [28], these were isolates from wild birds that were colonised from US hospital wastewater, as confirmed. Therefore, it can be concluded that the tested strains differed in the spectrum of antibiotic resistance, genetic make-up and, probably, accessory genome. In addition, all the compounds were evaluated in vitro against *Mycobacterium smegmatis* ATCC 700084 and *M. marinum* CAMP 5644 as a safe alternative to *M. tuberculosis*. The genus *Mycobacterium* is a closely related group of fast- and slow-growing species. Alternative model pathogens for *M. tuberculosis* can be used in laboratory studies to reduce risks and facilitate laboratory handling. *M. smegmatis* is an ideal representative of a fast-growing non-pathogenic microorganism particularly useful in the study of basic cellular processes of particular importance for pathogenic mycobacteria [29]. *M. marinum* is very closely related to *M. tuberculosis* and is the cause of TB-like infections in poikilothermic organisms, especially frogs and fish. *M. marinum* is a good model for study mainly due to the lower risk for laboratory workers, and its genetic relatedness and similar pathology to human TB [30]. Activities are expressed as the minimum inhibitory concentrations (MICs) and the minimum bactericidal concentrations (MBCs) as reported in Table 1. To establish that a compound demonstrates a bactericidal effect against a particular tested strain, it must meet the condition MIC/MBC ≤ 4 [27,31]. MBC values that fulfil this requirement, i.e., the compound is bactericidal, are indicated in bold in Table 1.

9-Benzyloxy-5-methyl-12*H*-quino[3,4-*b*][1,4]benzothiazinium chloride (**6j**) demonstrated the highest activity against the entire tested microbial spectrum. Furthermore, 9-propoxy-5-methyl-12*H*-quino[3,4-*b*][1,4]benzothiazinium chloride (**6a**) and 9-allyloxy-5-methyl-12*H*-quino[3,4-*b*][1,4]benzothiazinium chloride (**6d**) also showed high activity. Compounds **6b**, **6e**, **6k** and **6l** expressed antistaphylococcal activity, while compound **6h** had only a moderate effect against enterococci. The efficacy of the active agents was comparable to the antibiotics clinically used against staphylococcal strains, especially against MRSA isolates. It should be noted that compounds showed comparable antistaphylococcal activities, both against methicillin-susceptible *S. aureus* and MRSA isolates; therefore, it can be assumed that the presence of the *mecA* gene (which encodes an alternative transpeptidase and causes methicillin resistance [27,32]) in MRSA does not affect the activity of these compounds. Thus, the above can be speculated concerning the specific activity against *Staphylococcus* sp. Similarly, the close activity of the compounds against both *E. faecalis* and VRE indicates a mechanism of action unrelated to vancomycin resistance [28]. It is important to mention that all compounds demonstrated bactericidal activity (see bolded MBC values in Table 1).

In addition to **6j**, **6a** and **6d**, the compounds **6k**, **6b**, **6e**, **6g** and **6h** proved to be active against fast-growing *M. smegnatis*, and compounds **6l**, **6e**, **6j** and **6g** were effective against slow-growing *M. marinum*. All the mentioned compounds were more effective than isoniazid, and their activities were comparable with rifampicin against both mycobacterial strains.

If these compounds are compared with recently described ones [25], it can be stated that there was a significant increase in antibacterial activity. The absence of heteroatoms in positions 8 or 10, and the replacement of halogens by alkoxy substituents on C_(9)_ resulted in significant progress in the design of quino[3,4-*b*][1,4]benzothiazines as antimicrobial agents [25]. Based on the data presented in Table 1, it can be summarised that the substitution by the benzyloxy or propoxy chain appears to be the most advantageous, specifically in the 9 position, followed by the 10 position. The position 11 is completely disadvantageous in terms of antimicrobial activity. This empirical reasoning is supported by a multidimensional-based study of structure–property and structure–activity relationships (see Section 2.4). In addition, similar observations were found in the previous studies, where the highest antistaphylococcal and antimycobacterial activities were shown by derivatives substituted in the 9 or 10 position with branched alkoxy tails [33,34] or with a phenylalkyl chain [35,36,37], which mimics the benzyloxy substituent used here.

Since the compounds act more on aerobic staphylococci than on facultatively anaerobic enterococci [38], known among other things for high resistance to disinfection procedures and antibiotics [39,40,41], it was hypothesised that a possible mechanism of action could be inhibition of respiration and, therefore, a standard MTT test was performed with the most active derivatives. The MTT assay can be used to assess cell growth by measuring respiration. Respiratory activity of bacterial cells (which is finally reflected in their viability) of less than 70% after exposure to the MIC values for each tested compound is considered as a positive result of this assay. This low level of cell oxidative metabolism indicates inhibition of cell growth by inhibition of respiration [42,43]. The lowest multiples of the MIC values by which inhibition of *S. aureus* ATCC 29213 viability (%) greater than 70% was achieved are given in Table 2. It can be concluded that evaluated compounds **6a**, **6e**, **6g** and **6k** did not show a decrease in viability < 70% at its MIC value, which suggests that the main mechanism of action is not inhibition of the respiratory chain, although they are able to significantly affect it, compared to, e.g., ciprofloxacin. On the other hand, compound **6j** (R = 9-OCH_2_C_6_H_5_) demonstrated strong inhibition of the respiratory chain (0.5×MIC/0.5×MBC).

**Figure 2:**
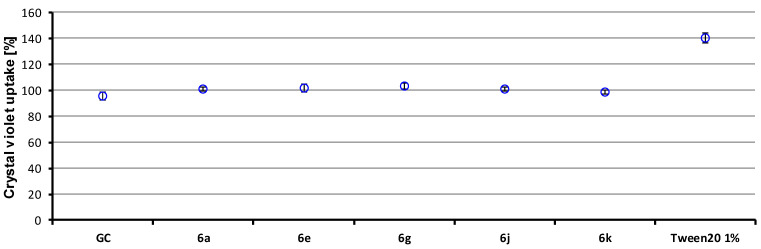
Uptake of crystal violet by *S. aureus* (GC = growth control).

Phenothiazines are also known for their ability to interact with and damage the cell wall, which has fatal consequences for the microorganism [44,45]. Therefore, the bacterial suspensions were treated by compounds **6a**, **6e**, **6g**, **6j** and **6k** (4× MIC) for 1 h. The uptake of crystal violet was expressed as a percentage compared to the original crystal violet solution. The results are shown in Figure 2. The test compounds did not affect plasma membrane permeability of *S. aureus*, because the percentage absorption of crystal violet was comparable to the growth control and disproportionately lower than the 1% Tween 20 solution used as the positive control. Based on these results, it could be assumed that an increase in the membrane permeability was not the mechanism of action of these compounds.

As the antibacterial activity of the discussed compounds cannot be explained solely on the basis of inhibition of respiration, it is also very difficult to reach a conclusion regarding the actual mechanism of action of the antimycobacterial activity. Phenothiazines, like quinolines, are multi-target agents, i.e., they have the ability to affect several different targets, leading to the death of treated microorganisms. Quinolines are known to be able to inhibit, for example, the respiratory chain (possessing a similar mechanism of action to bedaquiline [46,47,48,49]), sulphur metabolic pathways [50] or mycobacterial FtsZ protein [51]; phenothiazines, in addition to the aforementioned ability to interact with the bacterial membrane, have the ability to inhibit the respiratory chain, dissipate the membrane potential, reduce the level of ATP, increase oxidative stress or increase the level of intracellular ions [44,45,52,53].

Therefore, the results obtained in this study will need to be supported by functional genomic and proteomic studies to reveal the mechanisms of actions of these 5-methyl-12*H*-quino[3,4-*b*][1,4]benzothiazinium chlorides on staphylococci and mycobacteria.

#### 2.3.2. In Vitro Cell Viability

A preliminary in vitro cytotoxicity screening of target compounds **6a**–**l** was performed using normal human dermal fibroblasts (NHDF) and was expressed as IC_50_ values (see Table 1). A compound is considered cytotoxic if it demonstrates a toxic effect on cells at a concentration up to 10 µM [54]. For example, the IC_50_ for doxorubicin was found to be 5.5 µM in this assay. The lowest concentration that affected human non-tumour cells was 37.7 µM for compound **6j**, i.e., more than three times the value at which the bioactive agent is considered cytotoxic. Even this fact does not prevent active substances from being considered interesting for further investigation and from being generally considered as insignificantly cytotoxic.

### 2.4. Similarity-Mediated Property Mapping

Many factors and parameters play an important role in the design and subsequent development of bioactive agents [55]. One of them is lipophilicity, which is among the most important of all investigated physicochemical properties, as it affects not only the ligand–target binding interaction, but also solubility and subsequent absorption (biological availability), binding to transporters, metabolism and excretion [56]. The lipophilicity of the studied compounds was determined using TLC as logP_TLC_ parameter (see Table 1).

**Figure 3:**
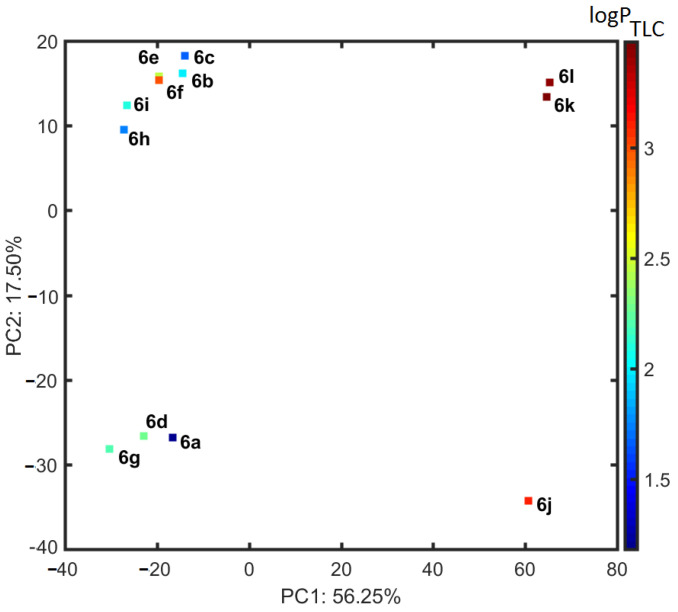
Projection of molecules **6a**–**l** on plane defined by PC1 and PC2 with corresponding experimental lipophilicity in logarithmic scale. Colours code logP_TLC_ values.

In practice, the clustering tendency of the descriptor-driven data can be investigated by tracing the (dis)similarities between objects in the multidimensional variable space; therefore, the distance-related property mapping was conducted using principal component analysis (PCA) and hierarchical clustering analysis (HCA) on the pool of 2666 descriptors produced by Dragon 6.0 software. The calculated data were organised into matrix X_12×2666_ with rows representing objects (compounds **6a**–**l**) and columns representing in silico parameters (descriptors). The resulting matrix was centred and standardised, because the numerical parameters varied noticeably. The percentage of the modelled data variance was taken into account in order to specify the number of relevant principal components (PCs). The first three PCs describe 87.75% of the total data variance, while the first two PCs account for 73.75%, respectively. The corresponding projection (scoreplot) of molecules **6a**–**l** on the plane defined by PC1 vs. PC2, and additionally colour-coded according to the empirical lipophilicity (logP_TLC_), is shown in Figure 3.

Not surprisingly, the benzyl-substituted isomers (**6j**–**l**) are located separately (PC1 > 0) from the aliphatic derivatives **6a**–**i**, which are characterised by the negative values of the first component (PC1 < 0) and relatively lower lipophilic characteristics (logP_TLC_ < 3). The interesting distribution of analogues **6a**–**l** is observed according to the second principal component (PC2), where isomers **6a**, **d**, **g** and **j** are grouped together along the negative values of PC2. Bafflingly, derivatives with substituents in positions 10 and 11 are clustered together on the positive part of PC2, as illustrated in Figure 3.

**Figure 4:**
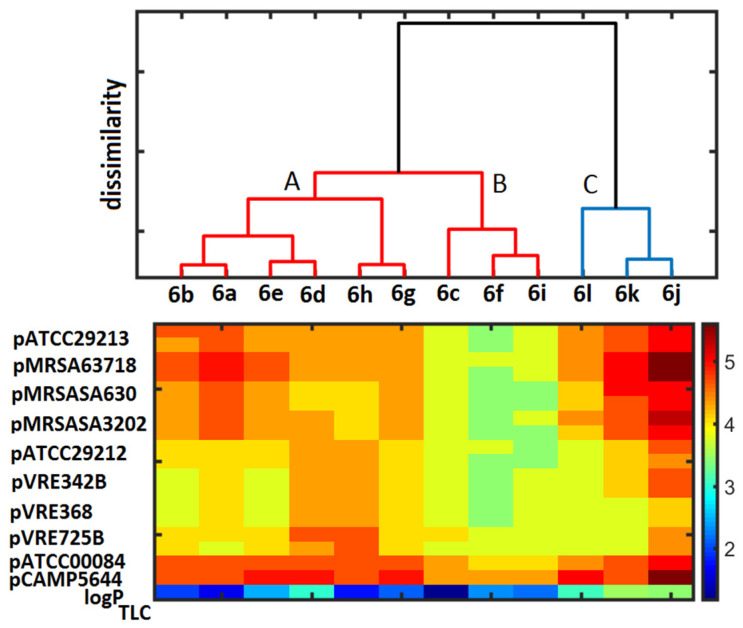
Dendrogram of molecules **6a**–**l** in descriptor-based space with colour-coded map of biological and lipophilic data in logarithmic scale.

In an attempt to relate the (dis)similarity between objects (molecules) in the multidimensional descriptor-based space to the molecular property profile (e.g., biological or lipophilic compound characteristics), hierarchical clustering analysis (HCA) was combined with a colour-coded map of the empirical dataset [25,57]. On the whole, the exploratory HCA approach produces the sub-optimal clustering pattern of objects that is largely dependent on the clusters’ linkage procedure engaged; therefore, the similarity measure (e.g., Euclidean distance) as well as the manner of resulting sub-clusters’ linkage (e.g., Ward’s algorithm) need to be chosen a priori [58]. A dendrogram in Figure 4, conjugated with the colourful display of experimental data (e.g., biological activities and lipophilic characteristics) allows for the direct interpretation of the produced clusters in terms of the original parameters, where OX illustrates the order of objects and the OY axis presents the dissimilarity, respectively. The exploratory HCA procedure generated the clustering pattern of objects that confirms our previous PCA findings (see Figure 3). Likewise, the benzyl-containing molecules **6j**–**l** vary noticeably (cluster C) from the remaining compounds (cluster A and B) of the dataset. Roughly speaking, molecules **6c**, **f** and **i** are grouped together in cluster B, which is characterised by noticeably lower values of antibacterial potency and lipophilic values, as shown in colour-coded vectors in Figure 4. An inverted SAR trend is observed for molecules **6a**, **b**, **d**, **e**, **g** and **h** (cluster A and C), which are generally marked by higher values of biological activities. On the other hand, there is no evident SPR (structure–property) relationship with logP_TLC_ in the **6a**, **b**, **d**, **e**, **g** and **h** subunit, because these isomers in cluster C are described by higher lipophilic values.

## 3. Materials and Methods

### 3.1. Chemisty

Melting points are uncorrected. NMR spectra were recorded using a Bruker Ascend 600 spectrometer (Bruker, Billerica, MA, USA). To assign the structures, the following 2D experiments were employed: ^1^H-^13^C gradient selected HSQC and HMBC sequences. Standard experimental conditions and standard Bruker programs were used. The ^1^H NMR and ^13^C NMR spectral data are provided relative to the TMS signal at 0.0 ppm. HR mass spectra were recorded with Bruker Impact II (Bruker, Billerica, MA, USA).

#### 3.1.1. Synthesis of Acetanilide Derivatives **2a**–**l**

A solution of 10 mmol of the appropriate hydroxyacetanilide (**1**) in 10 mL of anhydrous DMF was added portionwise to a suspension of 10 mmol NaH (60% suspension in mineral oil) in 10 mL dry DMF with stirring. To the reaction mixture obtained, 12 mmol of the alkylating agent were added in portions while stirring. After 12 h the mixture was poured into 100 mL of water and extracted with chloroform (3 × 15 mL). The combined extracts were dried over anhydrous sodium sulphate. After evaporation of the solvent in vacuo, the dry residue was purified by aluminium oxide column chromatography, eluting with chloroform: ethanol *v*/*v* 10:1. 

*2-(propoxy)acetanilide* (**2a**): yield: 92%; ^1^H NMR (CDCl_3_, 600 MHz), δ(ppm): 1.02–1.10 (t, *J* = 7.2 Hz, 3H,CH_2_-CH_3_), 1.75–1.83 (m, 2H, CH_2_CH_2_CH_3_), 2.21 (s, 3H, COCH_3_) 3.97–4.07 (t, *J* = 6.6 Hz, 2H, CH_2_CH_2_CH_3_), 6.83–6.85 (m, 1H, H_arom_), 6.85–6.97 (m, 1H, H_arom_), 6.99–7.05 (m, 1H, H_arom_), 7.82 (s, 1H, NH), 6.83–6.88 (m, 1H, H_arom_); ^13^C NMR, (CDCl_3_, 150.9 MHz) δ (ppm): 9.93, 21.48, 23.89, 69.08, 109.92, 118.73, 1189.86, 122.56, 126.81, 148.06, 168.14; ESI-HRMS Calcd for C_11_H_16_NO_2_ ([M + H]^+^): 194.1181, found: 194.1177.

*3-(propoxy)acetanilide* (**2b**): yield: 95%; ^1^H NMR (CDCl_3_, 600 MHz), δ (ppm): 0.96–1.07 (t, *J* = 7.2 Hz, 3H,CH_2_-CH_3_), 1.73–1.83 (m, 2H, CH_2_CH_2_CH_3_), 2.15 (s, 3H, COCH_3_) 3.82–3.89 (t, *J* = 6.6 Hz, 2H, CH_2_CH_2_CH_3_), 6.60–6.73 (m, 1H, H_arom_), 6.95–6.99 (m, 1H, H_arom_), 7.05–7.15 (m, 1H, H_arom_), 7.23–7.30 (m, 1H, H_arom_), 8.53 (s, 1H, NH), ^13^C NMR (CDCl_3_, 150.9 MHz), δ (ppm): 9.46, 21.50, 23.37, 68.43 105.38, 109.45, 111.04, 128.44, 138.39, 158.56, 167.10; ESI-HRMS Calcd for C_11_H_16_NO_2_ ([M + H]^+^): 194.1181, found: 194.1175.

*4-(propoxy)acetanilide* (**2c**): yield: 96%; ^1^H NMR (DMSO, 600 MHz), δ (ppm): 0.92–1.00 (t, *J* = 7.2 Hz, 3H, CH_2_-CH_3_), 1.64–1.73 (m, 2H, CH_2_CH_2_CH_3_), 2.03 (s, 3H, COCH_3_), 3.80–3.90 (t, *J* = 6.6 Hz, 2H, CH_2_CH_2_CH_3_), 6.80–6.87 (m, 2H, H_arom_), 7.42–7.50 (m, 2H, H_arom_), 9.77 (s, 1H, NH); ^13^C NMR(DMSO, 150.9 MHz), δ (ppm): 10.99 (CH_3_), 22.55 (CH_2_), 24.28 (COCH_3_), 69.46 (OCH_2_), 114.79, 114.39, 120.94, 132.90, 154.88, 168.14; ESI-HRMS Calcd for C_11_H_16_NO_2_ ([M + H]^+^): 194.1181, found: 194.1172.

*2-(allyloxy)acetanilide* (**2d**): yield: 94%: ^1^H NMR (CDCl_3_, 600 MHz), δ (ppm): 2.19 (s, 3H, CH_3_), 4.53–4.65 (m, 2H, CH_2_-CH=CH_2_), 5.30–5.38 (m, 1H, CH_2_-CH=CH_2_), 5.38–5.45 (m, 1H, CH_2_-CH=CH_2_), 5.98–6.15 (m, 1H, CH_2_-CH=CH_2_), 6.80–6.89 (m, 1H, H_arom_), 6.89–7.05 (m, 1H, H_arom_), 7.78–7.89 (m, 1H, H_arom_), 8.30–8.40 (m, 1H, H_arom_); ^13^C NMR (CDCl_3_, 150.9 MHz), δ (ppm): 24.28 (CH_3_), 69.79 (CH_2_-CH), 115.23, 120.93, 128.14, 128.23, 128.86, 133.22, 137.68, 154.52, 168.20 (C=O); ^13^C NMR (CDCl_3_, 150.9 MHz), δ (ppm): 24,93 (CH_3_), 69.48 (CH_2_), 111,37, 118.21, 119.97, 121.26, 123.57, 127.93, 132.84, 146.72, 168.21; ESI-HRMS Calcd for C_11_H_14_NO_2_ ([M + H]^+^): 192.1024, found: 192.1018.

*3-(allyloxy)acetanilide* (**2e**): yield: 94%: ^1^H NMR (CDCl_3_, 600 MHz), δ (ppm): 2.06 (s, 3H, CH_3_), 4.35–4.48 (m, 2H, CH_2_-CH=CH_2_), 5.05–5.20 (m, 1H, CH_2_-CH=CH_2_), 5.20–5.37 (m, 1H, CH_2_-CH=CH_2_), 5.80–98 (m, 1H, CH_2_-CH=CH_2_), 6.50–6.58 (m, 1H, H_arom_), 6.90–6.98 (m, 1H, H_arom_), 7.03–7.10 (m,1H, H_arom_), 7.25–7.30 (m, 1H, H_arom_), 8.55 (s, 1H, NH); ^13^C NMR (CDCl_3_, 150.9 MHz), δ (ppm): 24.41 (CH_3_), 68.76 (CH_2_-CH), 106.60, 110.61, 112.40, 117.63, 129.52, 133.17, 133.17, 139.51, 159.01, 169.22 (C=O); ESI-HRMS Calcd for C_11_H_14_NO_2_ ([M + H]^+^): 192.1024, found: 192.1012.

*4-(allyloxy)acetanilide* (**2f**): yield: 90%: ^1^H NMR (CDCl_3_, 600 MHz), δ (ppm): 2.15 (s, 3H, CH_3_), 4.48–4.48 (m, 2H, CH_2_-CH=CH_2_), 5.20–5.28 (m, 1H, CH_2_-CH=CH_2_), 5.35–5.42 (m, 1H, CH_2_-CH=CH_2_), 5.98–6.10 (m, 1H, CH_2_-CH=CH_2_), 6.82–6.88 (m, 2H, H_arom_), 7.35–7.42 (m, 2H, H_arom_), 7.90 (s, 1H, NH); ^13^C NMR (CDCl_3_, 150.9 MHz), δ (ppm): 23.09 (CH_3_), 68.04 (CH_2_-CH), 113.95, 116.67 (CH=CH_2_), 120.93, 130.17 (CH=CH_2_), 132.20 154.40, 167.69 (C=O); ESI-HRMS Calcd for C_11_H_14_NO_2_ ([M + H]^+^): 192.1024, found: 192.32.

*2-(propargyloxy)acetanilide* (**2g**): yield: 87%: ^1^H NMR (CDCl_3_, 600 MHz), δ (ppm): 2.02 (s, 3H, CH_3_), 2.50–2.52 (t, *J* = 2.4 Hz, 1H, CH), 4.53–4.59 (d, *J* = 2.4 Hz, 2H, CH_2_), 6.75–6.90 (m, 3H, H_arom_), 7.93 (s, 1H, NH), 8.11–8.16 (m, 1H, H_arom_); ^13^C NMR (CDCl_3_, 150.9 MHz), δ (ppm): 24.57, 56.37, 76.28, 73.03, 111.87, 120.44, 121.71, 123.49, 128.10, 146.06, 168.40; ESI-HRMS Calcd for C_11_H_12_NO_2_ ([M + H]^+^): 190.0868, found: 190.0863.

*3-(propargyloxy)acetanilide* (**2h**): yield: 87%: ^1^H NMR (CDCl_3_, 600 MHz), δ (ppm): 2.06 (s, 3H, CH_3_), 2.42–2.51 (t, *J* = 2.4 Hz, 1H, CH), 4.49–4.55 (d, *J* = 2.4 Hz, 2H, CH_2_), 6.56–6.61 (m, 1H, H_arom_), 7.05–7.12 (m, 2H, H_arom_), 7.25–7.31 (m, 1H, H_arom_), 9.26 (s, 1H, NH); ^13^C NMR (CDCl_3_, 150.9 MHz), δ (ppm): 24.26, 55.71, 75.71, 78.55, 110.30, 113.21, 129.50, 139.80, 157.84, 169.58; ESI-HRMS Calcd for C_11_H_12_NO_2_ ([M + H]^+^): 190.0868, found: 190.0865.

*4-(propargyloxy)acetanilide* (**2i**): yield: 92%: ^1^H NMR (CDCl_3_, 600 MHz), δ (ppm): 2.06 (s, 3H, CH_3_), 2.42–2.52 (t, *J* = 2.4 Hz, 1H, CH), 4.55–4.62 (d, *J* = 2.4 Hz, CH_2_), 6.80–6.85 (d, *J* = 9 Hz, 2H, H_arom_), 7.38–7.45 (d, *J* = 9 Hz, 2H, H_arom_), 8.88 (s, 1H, NH); ^13^C NMR (CDCl_3_, 150.9 MHz), δ (ppm): 24.01, 56.07, 75.57, 78.60, 115.07, 121.78, 132,56, 153,91, 168.98; ESI-HRMS Calcd for C_11_H_12_NO_2_ ([M + H]^+^): 190.0868, found: 190.0857.

*2-(benzyloxy)acetanilide* (**2j**): yield: 86%: ^1^H NMR (DMSO, 600 MHz), δ (ppm): 2.11 (s, 3H, CH_3_); 5.21 (s, 2H, CH_2_); 6.83–6.92 (m, 1H, H_arom_), 6.98–7.10 (m, 2H, H_arom_), 7.28–7.35 (m, 1H, H_arom_), 7.35–7.42 (m, 2H, H_arom_), 7.47–7.54 (m, 1H, H_arom_), 7.81–7.90 (m, 1H, H_arom_), 9.17 (s, 1H, NH); ^13^C NMR (DMSO, 150.9 MHz), δ (ppm): 24.28 (CH_3_), 70.02 (CH_2_), 113.31, 120.90, 123.33, 124.83, 127.75, 128.17, 128.22, 128.85, 137.58, 149.31, 168.97; ESI-HRMS Calcd for C_15_H_16_NO_2_ ([M + H]^+^): 242.1181, found: 242.1176.

*3-(benzyloxy)acetanilide* (**2k**): yield: 89%: ^1^H NMR (CDCl_3_, 600 MHz), δ (ppm): 2.19 (s, 3H, CH_3_), 5.07 (s, 2H, CH_2_), 6.71–6.78 (m, 1H, H_arom_), 6.96–7.03 (m, 1H, H_arom_), 7.15–7.25 (m, 1H, H_arom_), 7.29–7.38 (m, 1H, H_arom_), 7.39–7.48 (m, 5H, H_arom_); ^13^C NMR (CDCl_3_, 150.9 MHz), δ (ppm): 24.70 (CH_3_), 70.01 (CH_2_), 106.55, 110.99, 112.22, 127.54, 127.98, 128.58, 129.71, 136.87, 139.11, 159.33, 168.46; ESI-HRMS Calcd for C_15_H_16_NO_2_ ([M + H]^+^): 242.1181, found: 242.1173.

*4-(benzyloxy)acetanilide* (**2l**): yield: 93%: ^1^H NMR (CDCl_3_, 600 MHz), δ (ppm): 2.16 (s, 3H, CH_3_), 5.05 (s, 2H, CH_2_), 6.90–6.97 (m, 2H, H_arom_), 7.37–7.49 (m, 7H, H_arom_), 7.79 (s, 1H, NH); ^13^C NMR (CDCl_3_, 150.9 MHz), δ (ppm): 24.15 (CH_3_), 70.29 (CH_2_), 115.15, 122.07, 127.50, 128.00, 128.60, 131,19, 136,93, 155.69, 168.77; ESI-HRMS Calcd for C_15_H_16_NO_2_ ([M + H]^+^): 242.1181, found: 242.1183.

#### 3.1.2. Synthesis of Aniline Derivatives **3a**–**l**

To a solution of 10 mmol of the appropriate acetanilide (**2**) in 30 mL of ethanol, 30 mL of 36% hydrochloric acid was added. The resulting mixture was heated (while stirring) at 60 °C for 24 h. After cooling to room temperature, the mixture was poured into 50 mL of water and extracted with chloroform (3 × 15 mL). The combined extracts were dried over anhydrous sodium sulphate. After evaporation of the solvent in vacuo, the dry residue was purified by aluminium oxide column chromatography, eluting with chloroform: ethanol *v*/*v* 10:1.

*2-(propoxy)aniline* (**3a**): oil; yield: 84%; ^1^H NMR (DMSO, 600 MHz) δ (ppm): 0.95–1.05 (t, *J* = 7.2 Hz, 3H, CH_2_-CH_3_), 1.70–1.79 (m, 2H, CH_2_CH_2_CH_3_), 3.82–3.93 (t, *J* = 6.3 Hz, 2H, CH_2_CH_2_CH_3_), 4.64 (s, 2H, NH_2_), 6.48–6.56 (m, 1H, H_arom_), 6.65–6.72 (m, 2H, H_arom_), 6.75–6.79 (m, 1H, H_arom_); ^13^C NMR (CDCl_3_, 150.9 MHz), δ (ppm): 10.99 (CH_3_), 22.73 (CH_2_), 69.59 (OCH_2_), 112.03, 114.39, 116.73, 121.23, 138.19, 146.13; ESI-HRMS Calcd for C_9_H_14_NO ([M + H]^+^): 152.1075, found: 152.1067.

*3-(propoxy)aniline* (**3b**): oil; yield: 92%; ^1^H NMR (DMSO, 600 MHz) δ (ppm): 0.90–1.02 (t, *J* = 7.4 Hz, 3H, CH_2_-CH_3_), 1.62–1.71 (m, 2H, CH_2_CH_2_CH_3_), 3.73–3.82 (t, *J* = 6.6 Hz, 2H, CH_2_CH_2_CH_3_), 5.02 (s, 2H, NH_2_), 6.02–6.10 (m,1H, H_arom_), 6.12–6.20 (m, 2H, H_arom_), 6.85–6.92 (m, 1H, H_arom_); ^13^C NMR (CDCl_3_, 150.9 MHz), δ (ppm):): 10.89 (CH_3_), 22.64 (CH_2_), 68.84 (OCH_2_), 100.48, 102.53, 107.20, 129.94, 150.38, 160.16; ESI-HRMS Calcd for C_9_H_14_NO ([M + H]^+^): 152.1075, found: 152.1085.

*4-(propoxy)aniline* (**3c**): oil; yield: 85%; ^1^H NMR (DMSO, 600 MHz) δ (ppm): 0.91–0.98 (t, *J* = 7.2 Hz, 3H, CH_2_-CH_3_), 1.60–1.69 (m, 2H, CH_2_CH_2_CH_3_), 3.73–3.78 (t, *J* = 6.6 Hz, 2H, CH_2_CH_2_CH_3_), 4.59 (s, 2H, NH_2_), 6.46–6.53 (m, 2H, H_arom_), 6.60–6.65 (m, 2H, H_arom_); ^13^C NMR (DMSO, 150.9 MHz), δ (ppm): 10.94 (CH_3_), 22.72 (CH_2_), 69.88 (OCH_2_), 115.41, 115.77, 142.74, 150.47; ESI-HRMS Calcd for C_9_H_14_NO ([M + H]^+^): 152.1075, found: 152.1077.

*2-(allyloxy)aniline* (**3d**): oil; yield: 82%; ^1^H NMR (CDCl_3_, 600 MHz), δ (ppm): 3.73 (s, 2H, NH_2_), 4.52–4.62 (d, *J* = 4.8 Hz, 2H, CH_2_-CH=CH_2_), 5.25–5.32 (m, 1H, CH_2_-CH=CH_2_), 5.38–5.48 (m, 1H, CH_2_-CH=CH_2_), 6.02–6.12 (m, 1H, CH_2_-CH=CH_2_), 6.70–6.78 (m, 2H, H_arom_), 6.80–6.90 (m, 2H, H_arom_); ^13^C NMR (CDCl_3_ 150.9 MHz), δ (ppm): 69.16 (OCH_2_), 112.00, 115.20, 117.41, 118.30, 121.37, 133.58, 136.52, 146.21; ESI-HRMS Calcd for C_9_H_12_NO ([M + H]^+^): 150.0918, found: 150.0915.

*3-(allyloxy)aniline* (**3e**): oil; yield: 89%; ^1^H NMR (CDCl_3_, 600 MHz), δ (ppm): 3.56 (s, 2H, NH_2_), 4.52–4.62 (d, *J* = 3.6 Hz, 2H, CH_2_-CH=CH_2_), 5.25–5.32 (m, 1H, CH_2_-CH=CH_2_), 5.38–5.48 (m, 1H, CH_2_-CH=CH_2_), 5.98–6.12 (m, 1H, CH_2_-CH=CH_2_), 6.32–6.40 (m, 3H, H_arom_), 6.98–7.10 (m, 1H, H_arom_); ^13^C NMR (CDCl_3_ 150.9 MHz), δ (ppm): 68.63 (OCH_2_), 101.85, 104.68, 108.09, 117.49, 130.08, 133.48, 147.84, 159.75; ESI-HRMS Calcd for C_9_H_12_NO ([M + H]^+^): 150.0918, found: 150.0909.

*4-(allyloxy)aniline* (**3f**): oil; yield: 80%; ^1^H NMR (CDCl_3_, 600 MHz), δ (ppm): 3.48 (s, 2H, NH_2_), 4.42–4.50 (d, *J* = 4.8 Hz, 2H, CH_2_-CH=CH_2_), 5.22–5.31 (m, 1H, CH_2_-CH=CH_2_), 5.35–5.45 (m, 1H, CH_2_-CH=CH_2_), 6.02–6.12 (m, 1H, CH_2_-CH=CH_2_), 6.60–6.69 (d, *J* = 8.4 Hz, 2H, H_arom_), 6.72–6.80 (d, *J* = 8.4 Hz, 2H, H_arom_); ^13^C NMR (CDCl_3_ 150.9 MHz), δ (ppm): 69.59 (OCH_2_), 115.93, 116.38, 117.39, 133.84, 140.26, 151.72; ESI-HRMS Calcd for C_9_H_12_NO ([M + H]^+^): 150.0918, found: 150.0911.

*2-(propargyloxy)aniline* (**3g**): oil; yield: 85%; ^1^H NMR (CDCl_3_, 600 MHz), δ (ppm): 2.50–2.60 (m, 1H, CH), 3.76 (s, 2H, NH_2_), 4.70–4.78 (m, 2H, CH_2_), 6.20–6.30 (m, 2H, H_arom_), 6.85–6.90 (m, 1H, H_arom_), 7.92–6.97 (m, 1H, H_arom_);^13^C NMR (CDCl_3_, 150.9 MHz), δ (ppm): 56.37 (CH_2_), 75.48 (CH), 78.84 (CH_2_-CCH), 112.62, 115.55, 118.35, 122.29, 136.68, 145.30; ESI-HRMS Calcd for C_9_H_10_NO ([M + H]^+^): 148.0765, found: 148.0746.

*3-(propargyloxy)aniline* (**3h**): oil; yield: 88%; ^1^H NMR (CDCl_3_ 600 MHz), δ (ppm): 2.38–2.45 (m, 1H, CH), 3.37 (s, 2H, NH_2_), 4.57–4.66 (m, 2H, CH_2_), 6.28–6.36 (m, 2H, H_arom_), 6.36–6.43 (m, 1H, H_arom_), 7.02–7.12 (m, 1H, H_arom_); ^13^C NMR (CDCl_3_, 150.9 MHz), δ (ppm): 56.21 (CH_2_), 75.41 (CH), 78.82 (CH_2_-CCH), 101.97, 104.63, 108.77, 130.14, 147.85, 158.73; ESI-HRMS Calcd for C_9_H_10_NO ([M + H]^+^): 148.0765, found: 148.0758.

*4-(propargyloxy)aniline* (**3i**): oil; yield: 92%; ^1^H NMR (CDCl_3_, 600 MHz), δ (ppm): 2.48–2.55 (m, 1H, CH), 3.52 (s, 2H, NH_2_), 4.60–2.65 (m, 2H, CH_2_), 6.62–6.70 (m, 2H, H_arom_), 6.80–6.88 (m, 2H, H_arom_), ^13^C NMR (CDCl_3_, 150.9 MHz), δ (ppm): 56.73 (CH_2_), 75.18 (CH), 79.12 (CH_2_-CCH), 116.27, 116.38, 149.86, 150.70; ESI-HRMS Calcd. for C_9_H_10_NO ([M + H]^+^): 148.0765, found: 148.0761.

*2-(benzyloxy)aniline* (**3j**): oil; yield: 83%; ^1^H NMR (CDCl_3_, 600 MHz), δ (ppm): 4.76 (s, 2H, NH_2_), 5.09 (s, 2H, CH_2_), 6.48–6.56 (m, 1H, H_arom_), 6.68–6.74 (m, 2H, Ha_rom_), 6.87–6.90 (m, 1H, H_arom_), 7.30–7.37 (m, 1H, H_arom_), 7.37–7.42 (m, 2H, H_arom_), 7.48–7.53 (m, 2H, H_arom_); ^13^C NMR (CDCl_3_, 150.9 MHz), δ (ppm):69.77 (CH_2_), 112.66, 114.65, 116.68, 121.67, 127.87, 128.11, 128.84, 138.02, 138.47, 145.77; ESI-HRMS Calcd for C_13_H_14_NO ([M + H]^+^): 200.1075, found: 200.1076.

*3-(benzyloxy)aniline* (**3k**): oil; yield: 85%; ^1^H NMR (CDCl_3_, 600 MHz), δ (ppm): 3.78 (s, 2H, NH_2_), 5.04 (s, 2H, CH_2_), 6.39–6.38 (m, 2H, H_arom_), 6.41–6.47 (m, 1H, H_arom_), 7.03–7.11 (m, 1H, H_arom_), 7.30–7.47 (m, 5H, H_arom_); ^13^C NMR (CDCl_3_, 150.9 MHz), δ (ppm): 69.81 (CH_2_), 102.09, 104.95, 108.31, 127.50, 127.90, 128.58, 130.15, 137.23, 147.66, 159.99; ESI-HRMS Calcd for C_13_H_14_NO ([M + H]^+^): 200.1075, found: 200.1070.

*4-(benzyloxy)aniline* (**3l**): oil; yield: 80%; ^1^H NMR (CDCl_3_, 600 MHz), δ (ppm): 3.76 (s, 2H, NH_2_), 5.02 (s, 2H, CH_2_), 6.68–6.73 (m, 2H, H_arom_), 6.82–6.88 (m, 2H, H_arom_), 7.30–7.49 (m, 5H, H_arom_); ^13^C NMR (CDCl_3_, 150.9 MHz), δ (ppm): 70.74 (CH_2_), 116.05, 116.72, 127.52, 127.84, 128.53, 137.45, 152.29, 162.58; ESI-HRMS Calcd for C_13_H_14_NO ([M + H]^+^): 200.1075, found: 200.1077.

#### 3.1.3. Synthesis of 5-Methyl-12H-Quino[3,4-b][1,4]Benzothiazine Chloride **6a**–**l**

Procedure A. 2.5 mmol of the appropriate aniline (**3**) was added to a suspension of 1 mmol (0.419 g) of 5,12-(dimethyl)thioquinantrenediinium bis-chloride (**4**) in 5 mL of anhydrous pyridine. The resulting reaction mixture was heated at 80 °C with vigorous (continuous) stirring to allow the introduction of atmospheric oxygen. After 24 h, the mixture was cooled to room temperature and the resulting solid was filtered off and washed with anhydrous ether (3 × 5 mL). The crude product was purified on an alumina chromatography column, eluting with chloroform: ethanol *v*/*v* 10:1.

Procedure B. 2.5 mmol of the appropriate aniline (**3**) was added to a suspension of 1 mmol (0.419 g) of 5,12-(dimethyl)thioquinantrenediinium bis-chloride (**4**) in 5 mL of anhydrous pyridine. The resulting reaction mixture was heated at 20 °C with vigorous (continuous) stirring to allow the introduction of atmospheric oxygen. After 7 days, the resulting solid was filtered off and washed with anhydrous ether (3 × 5 mL). The crude product was purified on an alumina chromatography column, eluting with chloroform: ethanol *v*/*v* 10:1.

*9-propoxy-5-methyl-12H-quino[3,4-b][1,4]benzothiazinium chloride* (**6a**): Procedure A. Yield: 82%; ^1^H NMR (DMSO, 600 MHz), δ (ppm): 0.90–1.00 (t, *J* = 7.5 Hz, 3H, CH_2_CH_3_), 1.62–1.75 (m, 2H, CH_2_CH_2_CH_3_), 3.80–3.89 (t, *J* = 6.3 Hz, 2H, CH_2_CH_2_CH_3_), 4.08 (s, 3H, NCH_3_), 6.58–6.63 (m, 1H, H_arom_), 6.63–6.70 (m, 1H, H_arom_), 7.54–7.61 (m, 1H, H_arom_), 7.92–8.00 (m, 2H, H_arom_), 8.50–8.60 (m, 2H, H_arom_), 9.02–9.12 (m, 1H, H_arom_), 11.28 (s, 1H, NH); ^13^C NMR (DMSO, 150.9 MHz), δ (ppm): 10.80 (CH_3_), 22.39 (CH_2_), 42.75 (NCH_3_), 69.88 (OCH_2_), 104.98, 113.04, 114.07, 118.52, 118.86, 120.50, 124.42, 124.82, 134.65, 136.71, 139.08, 142.84, 150.00, 151.00, 158.03; ESI-HRMS Calcd for C_19_H_19_N_2_OS ([M]^+^): 323.1218, found: 323.1217.

*10-propoxy-5-methyl-12H-quino[3,4-b][1,4]benzothiazinium chloride* (**6b**): Procedure B. Yield: 80%; ^1^H NMR (DMSO, 600 MHz), δ (ppm): 0.88–1.03 (t, *J* = 7.2 Hz, 3H, CH_2_CH_3_), 1.65–1.80 (m, 2H, CH_2_CH_2_CH_3_), 3.80–3.90 (t, *J* = 6.3 Hz, 2H, CH_2_CH_2_CH_3_), 4.114 (s, 3H, NCH_3_), 6.62–6.67 (m, 1H, H_arom_), 6.89–6.94 (m, 1H, H_arom_), 7.38–7.45 (m, 1H, H_arom_), 7.75–7.84 (m, 1H, H_arom_), 7.95–8.09 (m, 2H, H_arom_), 8.67 (s, 1H, H6), 9.12–9.20 (m, 1H, H_arom_), 11.26 (s, 1H, NH); ^13^C NMR (DMSO, 150.9 MHz), δ (ppm): 10.80 (CH_3_), 22.34 (CH_2_), 43.08 (NCH_3_), 69.76 (OCH_2_), 106.48, 106.75, 106.99, 113.20, 116.09, 119.12, 124.76, 127.83, 128.17, 134.78, 137.91, 139.04, 143.58, 151.76, 159.32; ESI-HRMS Calcd for C_19_H_19_N_2_OS ([M]^+^): 323.1218, found: 323.1256.

*11-propoxy-5-methyl-12H-quino[3,4-b][1,4]benzothiazinium chloride* (**6c**): Procedure A. Yield: 76%; ^1^H NMR (DMSO, 600 MHz), δ (ppm): 0.99-1.05 (t, *J* = 7.5 Hz, 3H, CH_2_CH_3_), 1.80–1.90 (m, 2H, CH_2_CH_2_CH_3_), 4.02–4.12 (t, *J* = 6.3 Hz, 2H, CH_2_CH_2_CH_3_), 4.19 (s, 3H, NCH_3_), 6.70–6.74 (m, 1H, H_arom_), 697–7.02 (m, 1H, H_arom_), 7.08–7.13 (m, 1H, H_arom_), 7.86–7.92 (m, 1H, H_arom_), 8.06–8.10 (m, 1H, H_arom_), 8.10–8.14 (m, 1H, H_arom_), 8.40–8.45 (m, 1H, H_arom_), 8.84 (s, 1H, H6), 9.84 (s, 1H, NH); ^13^C NMR (DMSO, 150.9 MHz), δ (ppm): 10.84 (CH_3_), 22.30 (CH_2_), 43.34 (NCH_3_), 71.01 (OCH_2_), 107.77, 113.18, 116.30, 118.89, 119.32, 119.41, 123.50, 125.57, 128.11, 128.56, 135.01, 139.14, 144.31, 148.25, 152.02; ESI-HRMS Calcd for C_19_H_19_N_2_OS ([M]^+^): 323.1218, found: 323.1215.

*9-allyloxy-5-methyl-12H-quino[3,4-b][1,4]benzothiazinium chloride* (**6d**): Procedure A. Yield: 78%; ^1^H NMR (DMSO_d-6_, 600 MHz), δ(ppm): 4.09 (s, 3H, CH_3_), 4.49–4.55 (d, *J* = 4.8 Hz, 2H, CH_2_-CH=CH_2_), 5.25–5.30 (m, 1H, CH=CH_2_), 5.35–5.45 (m, 1H, CH=CH_2_), 5.95–6.10 (m, 1H, CH=CH_2_), 6.63–6.73 (m, 2H, H_arom_), 7.50–7.58 (m, 1H, H_arom_), 7.72–7.80 (m, 1H, H_arom_), 7.95–8.05 (m, 2H, H_arom_), 8.57 (s, 1H, H6), 8.98–9.05 (m, 1H, H_arom_), 11.20 (s, 1H, NH); ^13^C NMR (DMSO, 150.9 MHz), δ (ppm): 42.80 (CH_3_), 69.04 (CH_2_), 105.02, 113.38, 114.39, 115.83, 118.27, 118.51, 118.95, 120.39, 124.71, 127.88, 129.59, 133.75, 134.72, 139.09, 142.99, 151.08, 157.50; ESI-HRMS Calcd for C_19_H_17_N_2_OS ([M]^+^): 321.1062, found: 321.1056.

*10-allyloxy-5-methyl-12H-quino[3,4-b][1,4]benzothiazinium chloride* (**6e**): Procedure B. Yield: 78%; ^1^H NMR (DMSO, 600 MHz), δ (ppm): 4.14 (s, 3H, CH_3_), 4.48–4.54 (d, *J* = 4.8 Hz, 2H, CH_2_-CH=CH_2_), 5.28–5.32 (m, 1H, CH=CH_2_), 5.38–5.45 (m, 1H, CH=CH_2_), 5.95–6.10 (m, 1H, CH=CH_2_), 6.60–6.68 (m, 1H, H_arom_), 6.90–6.95 (m, 1H, H_arom_), 7.40–7.49 (m, 1H, H_arom_), 7.73–7.80 (m, 1H, H_arom_), 7.95–8.18 (m, 2H, H_arom_), 8.67 (s, 1H, H6), 9.10–9.19 (m, 1H,H_arom_), 11.27 (s, 1H, NH); ^13^C NMR (DMSO, 150.9 MHz), δ (ppm): 4.10 (CH_3_), 68.90 (CH_2_), 106.75, 106.98, 107.15, 113.33, 116.11, 118.46, 119.14, 124.74, 127.86, 128.20, 133.63, 134.80, 137.92, 139.05, 143.62, 151.76, 158.80; ESI-HRMS Calcd for C_19_H_17_N_2_OS ([M]^+^): 321.1062, found: 321.1063.

*11-allyloxy-5-methyl-12H-quino[3,4-b][1,4]benzothiazinium chloride* (**6f**): yield: Procedure A. 72%; ^1^H NMR (DMSO, 600 MHz), δ (ppm): 4.19 (s, 3H, CH_3_), 4.70–4.78 (d, *J* = 4.8 Hz, 2H, CH_2_-CH=CH_2_), 5.30–5.36 (m, 1H, CH=CH_2_), 5.45–5.55 (m, 1H, CH_2_-CH=CH_2_), 6.09–6.19 (m, 1H, CH=CH_2_), 6.70–6.75 (m, 1H, H_arom_), 6.95–7.0 (m, 1H, H_arom_), 7.05–7.13 (m, 1H, H_arom_), 7.85–7.92 (m, 1H, H_arom_), 8.05–8.15 (m, 2H, H_arom_), 8.45–8.52 (m, 1H, H_arom_), 8.85 (s, 1H, H6), 9.92 (s, 1H, NH); ^13^C NMR (DMSO, 150.9 MHz), δ (ppm): 43.32 (CH_3_), 70.17 (CH_2_), 107.71, 113.70, 116.30, 118.53, 119.09, 119.35, 119.58, 123.71, 125.70, 127.98, 128.52, 133.70, 135.01, 139.12, 144.27, 147.81, 152.02; ESI-HRMS Calcd for C_19_H_17_N_2_OS ([M]^+^): 321.1062, found: 321.1062.

*9-propargyloxy-5-methyl-12H-quino[3,4-b][1,4]benzothiazinium chloride* (**6g**): yield: Procedure A. 75%; ^1^H NMR (DMSO, 600 MHz), δ (ppm): 3.62–3.67 (m, 1H, CH), 4.09 (s, 3H, CH_3_), 4.76–4.97 (m, 2H, CH_2_), 6.70–6.82 (m, 2H, H_arom_), 7.45–7.58 (m, 1H, H_arom_), 7.73–7.82 (m, 1H, H_arom_), 7.94–8.08 (m, 2H, H_arom_), 8.59 (s, 1H, H6), 8.90–9.04 (m, 1H, H_arom_), 11.15 (s, 1H, NH); ^13^C NMR (DMSO, 150.9 MHz), δ (ppm): 42.86 (CH_3_), 56.32 (CH_2_), 79.15, 79.28, 105.07, 113.72, 114.79, 115.89, 118.54, 119.03, 120.25, 124.56, 127.98, 130.21, 134.78, 139.13, 143.15, 151.23, 156.48; ESI-HRMS Calcd for C_19_H_15_N_2_OS ([M]^+^): 319.0905, found: 319.0900.

*10-propargyloxy-5-methyl-12H-quino[3,4-b][1,4]benzothiazinium chloride* (**6h**): Procedure B. Yield: 71%; ^1^H NMR (DMSO, 600 MHz), δ (ppm): 3.64–3.68 (m, 1H, CH), 4.15 (s, 3H, CH_3_), 4.77–4.92 (m, 2H, CH_2_), 6.70–6.75 (m, 1H, H_arom_), 6.97–7.05 (m, 1H, H_arom_), 7.70–7.77 (m, 1H, H_arom_), 7.80–7.90 (m, 1H, H_arom_), 8.02–8.14 (m, 2H, H_arom_), 8.69 (s, 1H, H6), 8.88–8.97 (m, 1H, H_arom_), 10.98 (s, 1H, NH); ^13^C NMR (DMSO, 150.9 MHz), δ (ppm): 43.16 (CH_3_), 56.17 (CH_2_), 79.24, 79.55, 106.91, 107.07, 108.06, 113.34, 116.12, 119.28, 124.26, 127.98, 128.34, 14.87, 137.90, 139.07, 143.77, 151.74, 157.91; ESI-HRMS Calcd for C_19_H_15_N_2_OS ([M]^+^): 319.0905, found: 319.0903.

*11-propargyloxy-5-methyl-12H-quino[3,4-b][1,4]benzothiazinium chloride* (**6i**): Procedure A. Yield: 71%; ^1^H NMR (DMSO, 600 MHz), δ (ppm): 3.71 (t, *J* = 2.4 Hz, 1H, CH), 4.19 (s, 3H, CH_3_), 5.03 (d, *J* = 2.4 Hz, 2H, CH_2_), 6.78–6.83 (m, 1H, H_arom_), 7.03–7.18 (m, 2H, H_arom_), 7.82–7.90 (m, 1H, H_arom_), 8.02–8.14 (m, 2H, H_arom_), 8.52–8.58 (m,1H, H_arom_), 8.83 (s, 1H, H6), 9.96 (s. 1H, NH); ^13^C NMR (DMSO, 150.9 MHz), δ (ppm): 43.39 (CH_3_), 57.43 (CH_2_), 79.13, 79.82, 107.77, 114.15, 116.35, 119.24, 119.34, 120.29, 123.87, 126.19, 127.85, 128.52, 135.06, 139.11, 144.32, 146.82, 152.12; ESI-HRMS Calcd for C_19_H_15_N_2_OS ([M]^+^): 319.0905, found: 319.0901.

*9-benzyloxy-5-methyl-12H-quino[3,4-b][1,4]benzothiazinium chloride* (**6j**): Procedure A. Yield: 74%; ^1^H NMR (DMSO, 600 MHz), δ (ppm): 4.09 (s, 3H, CH_3_), 5.08 (s, 2H, CH_2_), 6.78–6.84 (m, 2H, H_arom_), 7.40–7.51 (m, 7H, H_arom_), 7.95–8.10 (m, 2H, H_arom_), 8.58 (s, 1H, H6), 8.87–8.99 (m, 1H, H_arom_), 11.08 (s, 1H, NH); ^13^C NMR (DMSO, 150.9 MHz), δ (ppm): 42.84 (CH_3_), 70.10 (CH_2_), 105.10, 113.66, 114.66, 118.56, 119.04, 120.30, 124.41, 124.44, 127.99, 128.27, 128.48, 128.95, 134.78, 136.76, 137.03, 139.13, 149.99, 151.12, 157.71; ESI-HRMS Calcd for C_23_H_19_N_2_OS ([M]^+^): 371.1218, found: 371.1226.

*10-benzyloxy-5-methyl-12H-quino[3,4-b][1,4]benzothiazinium chloride* (**6k**): yield: Procedure B. 71%; ^1^H NMR (DMSO, 600 MHz), δ (ppm): 4.14 (s, 3H, CH_3_), 5.08 (s, 2H, CH_2_), 6.70–6.82 (m, 1H, H_arom_), 6.95–7.04 (m, 1H, H_arom_), 7.34–7.38 (m, 1H, H_arom_), 7.38–7.51 (m, 5H, H_arom_), 7.79–7.88 (m, 1H, H_arom_), 7.96–8.14 (m, 2H, H_arom_), 8.68 (s, 1H, H_arom_), 9.05–9.12 (m, 1H, H_arom_), 11.18 (s, 1H, NH); ^13^C NMR (DMSO, 150.9 MHz), δ (ppm): 43.11 (CH_3_), 69.93 (CH_2_), 106.81, 107.04, 107.33, 113.45, 116.13, 119.18, 124.59, 127.94, 128.49, 128.55, 128.97, 129.00, 134.82, 136.97, 137.96, 139.06, 143.68, 151.77, 158.99; ESI-HRMS Calcd for C_23_H_19_N_2_OS ([M]^+^): 371.1218, found: 371.1217.

*11-benzyloxy-5-methyl-12H-quino[3,4-b][1,4]benzothiazinium chloride* (**6l**): Procedure A. Yield: 76%; ^1^H NMR (DMSO, 600 MHz), δ (ppm): 4.20 (s, 3H, CH_3_), 5.33 (s, 2H, CH_2_), 6.69–6.74 (m, 1H, H_arom_), 6.95–7.09 (m, 1H, H_arom_), 7.06–7.09 (m, 1H, H_arom_), 7.30–7.38 (m, 1H, H_arom_), 7.39–7.44 (m, 2H, H_arom_), 7.53–7.59 (m, 2H, H_arom_), 7.82–7.90 (m, 1H, H_arom_), 8.05–8.10 (m, 1H, H_arom_), 8.11–8.17 (m, 1H, H_arom_), 8.42–8.49 (m, 1H, H_arom_), 8.89 (s, 1H, H6), 9.97 (m, 1H, NH); ^13^C NMR (DMSO, 150.9 MHz), δ (ppm): 43.32 (CH_3_), 70.98 (CH_2_), 107.81, 114.11, 116.33, 119.13, 119.38, 119.72, 123.70, 125.15, 126.02, 127.92, 128.46, 128.48, 128.96, 135.02, 137.01, 139.11, 144.30, 147.83, 152.03; ESI-HRMS Calcd for C_23_H_19_N_2_OS ([M]^+^): 371.1218, found: 371.1225.

^1^HNMR and ^13^CNMR spectrum data are reported in Supplementary Materials.

### 3.2. X-ray Structural Analysis

C_19_H_15_ClN_2_OS, *M*_r_ = 354.85, red-brown block, 0.081 × 0.132 × 0.259 mm, monoclinic, space group *P*2_1_/*c*, *a* = 9.445(4), *b* = 13.847(5),*c* = 12.819(5) Å, *β* = 100.51(1)°, *V* = 1648(1) Å^3^, *Z* = 2, *D*_c_ = 1.430 g/cm^3^, *F*000 = 736, Bruker AXS D8 VENTURE, Mo*Kα* radiation, *λ* = 0.71073 Å, *T* = 293(2) K, 2*θ*_max_ = 55.06°, 40,268 reflections collected, 3711 unique (*R*_int_ = 0.047). The structure was solved and refined using the programs XT, VERSION 2018/2 [59] and SHELXL-2019/1 [60], respectively. Final *GooF* = 1.09, *R* = 0.05, *wR* = 0.125, *R* indices based on 3119 reflections with *I* > 2*σ*(*I*) (refinement on *F*^2^), 211 parameters, 0 restraints. Lp and absorption corrections applied, *μ* = 0.366 mm^−1^. CCDC deposition number 2178985.

### 3.3. Thin-Layer Chromatography

The lipophilicity parameters were determined experimentally using reverse phase thin-layer chromatography (RP-TLC). Chromatograms were prepared on RP-18F_254s_ plates (1.05559.0001, Merck, Germany) precoated with nonpolar silicone oil. Plates were developed in glass chromatography chambers previously saturated with vapor of mobile phase. The mobile phase was composed of 0.2M Tris buffer (pH = 7.4) and acetone in different concentrations, i.e., 50%, 60%, 70%, 80 and 90%. The chromatograms were visualised in UV light (λ = 254 nm). Determination of the R_F_ coefficient was carried out twice for all compounds; in all cases, acetone concentrations used. The final value of R_F_ is the mean of the two measurements. The obtained R_F_ coefficient was used to calculate the value of the R_M_ parameter according to the equation: R_M_ = log [(1/R_F_) − 1]. By extrapolating R_M_ values to zero acetone concentration, a relative R_M0_ lipophilicity parameter was obtained, according to the equation: R_M_ = R_M0_ + *b*C, where *b* is slope and C is concentration of acetone in mobile phase. Based on R_M0_ and the calibration curve, the values of logP_TLC_ for compounds **6a**–**l** were determined. The calibration curve was calculated using the relationship between R_M0_ of the standard molecules (acetanilide, p-kresol, p-bromoacetophenone, benzophenone, anthracene) and the corresponding logP parameters specified by the extraction method, respectively. R_M0_ parameters of the standard molecules and quinobenzothiazine derivatives **6a**–**l** were determined in the same empirical conditions. Experimental lipophilic values of the individual compounds in the logarithmic scale are reported in Table 1.

### 3.4. Biological Evaluation

#### 3.4.1. In Vitro Antibacterial Evaluation

In vitro antibacterial activity of the synthesised compounds was evaluated against representatives of multidrug-resistant bacteria, three clinical isolates of methicillin-resistant *S. aureus*: clinical isolate of animal origin, MRSA 63718 (Department of Infectious Diseases and Microbiology, Faculty of Veterinary Medicine, University of Veterinary Sciences, Brno, Czech Republic), carrying the *mecA* gene [32]; and MRSA SA 630 and MRSA SA 3202 [27] (National Institute of Public Health, Prague, Czech Republic), both of human origin. These three clinical isolates were classified as vancomycin-susceptible (but with higher MIC of vancomycin equal to 2μg*/*mL (VA2-MRSA), within the susceptible range for MRSA 63718) methicillin-resistant *S. aureus* (VS-MRSA) [27]. Vancomycin- and methicillin-susceptible *S. aureus* ATCC 29213 and vancomycin-susceptible *Enterococcus faecalis* ATCC 29212, obtained from the American Type Culture Collection, were used as the reference and quality control strains. Three *vanA* gene-carrying vancomycin-resistant isolates of *E. faecalis* (VRE 342B, VRE 368, VRE 725B) were provided by Oravcova et al. [28].

The minimum inhibitory concentrations (MICs) were evaluated by the microtitration broth method according to the CLSI, with some modifications [61,62]. The compounds were dissolved in DMSO (Sigma, St. Louis, MO, USA) to obtain concentration 10 µg/mL and diluted in a microtitration plate in an appropriate medium, i.e., Cation Adjusted Mueller–Hinton Broth (CaMH, Oxoid, Basingstoke, UK) for staphylococci, and Brain Heart Infusion Broth (BHI, Oxoid) for enterococci to reach the final concentration of 256–0.125 µg/mL. Microtiter plates were inoculated with test microorganisms so that the final concentration of bacterial cells was 10^5^. Ampicillin, oxacillin, tetracycline and ciprofloxacin (Sigma) were used as reference drugs. A drug-free control and a sterility control were included. The plates were incubated for 24 h at 37 °C for staphylococci and enterococci. After static incubation in the darkness in an aerobic atmosphere, the MIC was visually evaluated as the lowest concentration of the tested compound, which completely inhibited the growth of the microorganism. The experiments were repeated three times. The results are summarised in Table 1.

#### 3.4.2. Determination of Minimum Bactericidal Concentrations

For the above-mentioned strains/isolates, the agar aliquot subculture method was used as a test for bactericidal agents [63,64]. After the MIC value determination, the inoculum was transferred to CaMH (Oxoid) for staphylococci, and BHI (Oxoid) for enterococci medium using a multipoint inoculator. The plates were incubated in a thermostat at 37 °C for 24 h. The lowest concentration of test compound at which ≤5 colonies were obtained was then evaluated as MBC, corresponding to a 99.9% decrease in CFU relative to the original inoculum.

#### 3.4.3. MTT Assay

Compounds were prepared as previously stated and diluted in CaMH broth for *S.aureus* to achieve the desired final concentrations. *S. aureus* bacterial suspension in sterile distilled water at 0.5 McFarland was diluted 1:3. Inocula were added to each well by multi-inoculator. Diluted mycobacteria in broth free from inhibiting compounds were used as the growth control. All compounds were prepared in duplicate. Plates were incubated at 37 °C for 24 h for *S. aureus*. After the incubation period, 10% well volume of MTT (3-(4,5-dimethylthiazol-2-yl)-2,5-diphenyltetrazolium bromide) reagent (Sigma) was mixed into each well and incubated at 37 °C 1 h for *S. aureus*. Then, 100 µL of 17% sodium dodecyl sulphate in 40% dimethylformamide was added to each well. The plates were read at 570 nm. The absorbance readings from the cells grown in the presence of the tested compounds were compared with uninhibited cell growth to determine the relative percent inhibition. The percent inhibition was determined through MTT assay. The percent viability is calculated through the comparison of a measured value and that of the uninhibited control: % viability = OD_570E_/OD_570P_ × 100, where OD_570E_ is the reading from the compound-exposed cells, while OD_570P_ is the reading from the uninhibited cells (positive control). Cytotoxic potential is determined by a percent viability of <70% [42,43]. The results are summarised in Table 2.

#### 3.4.4. In Vitro Antimycobacterial Evaluation

The evaluation of in vitro antimycobacterial activity of the compounds was performed against *Mycobacterium marinum* CAMP 5644 and *M. smegmatis* ATCC 700084. The broth dilution micro-method in Middlebrook 7H9 medium (Difco, Lawrence, KS, USA) supplemented with ADC Enrichment (Difco) was used to determine the minimum inhibitory concentration (MIC), as previously described [62]. The compounds were dissolved in DMSO (Sigma), and the final concentration of DMSO did not exceed 2.5% of the total solution composition. The final concentrations of the evaluated compounds, ranging from 256 μg/mL to 0.125 μg/mL, were obtained by twofold serial dilution of the stock solution in a microtiter plate with sterile medium. Isoniazid and rifampicin (Sigma) were used as reference antibacterial drugs. Bacterial inocula were prepared by transferring colonies from culture to sterile water. The cell density was adjusted to 0.5 McFarland units using a densitometer (Densi-La-Meter, LIAP, Riga, Latvia). The final inoculum was made by 1:1000 dilution of the suspension with sterile water. Drug-free controls, sterility controls and controls consisting of medium and DMSO alone were included. The determination of results was performed visually after 3 days of static incubation in the darkness at 37 °C in an aerobic atmosphere for *M. smegmatis* and after 21 days of static incubation in the darkness at 28 °C in an aerobic atmosphere for *M. marinum*. The minimum inhibitory concentrations (MICs) were defined as the lowest concentration of the compound at which no visible bacterial growth was observed. The MIC value is routinely and widely used in bacterial assays and is a standard detection limit according to the CLSI [61]. The results are summarised in Table 1.

#### 3.4.5. In Vitro Cell Viability Analysis

Compounds were evaluated for their antiproliferative activity using NHDF (normal human dermal fibroblasts, ATCC, Manassas, VA, USA). The cultured cells were kept at 37 °C and 5% CO_2_. The cells were seeded (1 × 10^4^ cells/well/100 μL DMEM supplemented with 10% FCS and streptomycin/penicillin) into 96-well plates (Corning Inc., Corning, NY, USA). Cells were counted using a hemocytometer (Burker chamber) and phase contrast Olympus IX50 microscope equipped with Sony SSC-DC58 AP camera and Olympus DP10 digital camera. The cell viability of the compounds was determined using the Cell Proliferation Reagent WST-1 assay (Roche Molecular Biochemicals, Mannheim, Germany). The examined cells were exposed to the tested compounds (1 mg/mL DMSO stock) for 72 h at various concentrations (0.1–100 μg/mL). The control was included in order to eliminate the DMSO effect at the concentration used. Cell cultures were incubated with WST-1 (10 μL) for 1 h. The absorbance of the samples was measured against a background control at 450 nm using a microplate reader with a reference wavelength at 600 nm. The obtained results are expressed as means of at least two independent experiments performed in triplicate. The values of IC_50_ (compound concentration required to cause 50% inhibition) were calculated from the dose–response relationship with respect to control.

### 3.5. Principal Component and Hierarchical Clustering Analysis

The visual distribution of molecules in the experimental (FCS) and virtual (VCS) molecular 2D/3D space can be inspected using principal component analysis (PCA) and hierarchical clustering analysis (HCA). Briefly, PCA reduces the space dimensionality by transforming descriptor-based multi-dimensional data (mD) into 2D/3D space (scores and loadings) with a relatively small number of so-called principal components (PCs) in order to maximise the description of variance within the input data. The PCA model with *f* principal components for a data matrix *X* can be calculated according to simple formula:

*X* = *TP^T^* + *E* (1)

where *X* is a data matrix with *m* objects and *n* variables, *T* is the score matrix with dimensions (*m* × *f*), *P^T^* is a transposed matrix of loadings with dimensions (*f* × *n*) and *E* is a matrix of the residual variance (*m* × *n*) that is not explained by the first *f* principal components. Generally, the first few principal components usually describe data variance sufficiently.

In an attempt to investigate the (dis)similarities between objects in the descriptor-based mD space, hierarchical clustering analysis (HCA) can be combined with colour-coded vectors of empirical datasets. Practically, the similarity measure (e.g., Euclidean distance) as well as the manner of resulting sub-clusters’ linkage (e.g., Ward’s algorithm) should be specified a priori. A dendrogram augmented with visual map of experimental data allows to roughly examine structure–activity and structure–property relationships.

## 4. Conclusions

A new method for the preparation of quinobenzothiazine derivatives on the benzene ring has been developed. The method is based on the structural modification of isomeric hydroxylanilines (aminophenols) leading to aniline derivatives being substrates in the reaction of obtaining tetracyclic quinobenzothiazinium derivatives. The method allows for the introduction of various types of substituents in the 9, 10 and 11 positions of the quinobenzothiazine system. Compounds with such a structure have not been synthesised by other methods to date. Using this method of synthesis, a number of propoxy, allyloxy, propargyloxy and benzyloxy derivatives were obtained. The structure of the products was confirmed by NMR spectroscopy, HR-MS spectrometry and X-ray analysis. The developed method creates a wide range of possibilities for further modification of the structure of quinobenzothiazines by introducing further pharmacophore groups and creating hybrid systems with other structural systems that are important in the medical chemistry. It can be very helpful for finding structures with biological properties. 9-Benzyloxy-5-methyl-12*H*-quino[3,4-*b*][1,4]benzothiazinium chloride (**6j**) demonstrated the highest activity against the entire tested microbial spectrum. Furthermore, 9-propoxy-5-methyl-12*H*-quino[3,4-*b*][1,4]benzothiazinium chloride (**6a**) and 9-allyloxy-5-methyl-12*H*-quino[3,4-*b*][1,4]benzothiazinium chloride (**6d**) also showed high activity. The activity of the compounds were comparable with oxacillin, tetracycline and ciprofloxacin against staphylococcal strains and with rifampicin against both mycobacterial strains. Compound **6j** had a significant effect on the inhibition of bacterial respiration, as demonstrated by the MTT assay. Although none of the discussed compounds damaged the bacterial wall, the effective compounds demonstrated bactericidal activity. Preliminary in vitro cytotoxicity screening of the compounds performed using normal human dermal fibroblasts (NHDF) proved that the tested compounds showed an insignificant cytotoxic effect on human cells (IC_50_ > 37 µM).

Moreover, the intermolecular similarity of novel derivatives was analysed in the multidimensional space of the structure/property-related in silico descriptors using principal component analysis and hierarchical clustering analysis, respectively. Hence, the distance-oriented property distribution for newly synthesised series of isomers substituted on C_(9)_/C_(10)_/C_(11)_ was correlated with the experimental TLC lipophilic profile. Not surprisingly, the benzyl-containing molecules are located separately from the aliphatic derivatives, which are marked by relatively lower lipophilic values (logP_TLC_ < 3). Moreover, **6c**, **f** and **i** isomers are grouped together along the second principal component. In fact, the HCA findings confirmed the PCA results, where isomers **6c**, **f** and **i** compose cluster B, which is characterised by noticeably lower values of antibacterial potency and lipophilic logP_TLC_ values. The reverse SAR trend is observed for molecules **6a**, **b**, **d**, **e**, **g** and **h** (cluster A and C), which are generally marked by higher values of biological activities. Conversely, there is no evident structure–lipophilicity relationship in molecules **6a**, **b**, **d**, **e**, **g** and **h**, because isomers of cluster C are described by higher lipophilic values.

The antimicrobial activities of the discussed compounds and their negligible cytotoxicity make these multi-target compounds interesting candidates for further research. However, further molecular-biological studies at the genome and proteome level will be necessary to reveal the mechanisms of action.

## Figures and Tables

**Table 1:** In vitro antistaphylococcal, antienterococcal activities (MIC/MBC [μM]) compared to ampicillin (AMP), oxacillin (OXA), tetracycline (TTC) and ciprofloxacin (CPX). Antimycobacterial activities (MIC [μM]) compared to isoniazid (INH) and rifampicin (RIF), and in vitro cell viability on normal human dermal fibroblasts (NHDF). Experimental lipophilicity expressed as logP_TLC_.

No.	MIC [μM] MBC [μM]										IC_50_ [µM] NHDF	logP_TLC_
	SA	MRSA1	MRSA2	MRSA3	EF	VRE1	VRE2	VRE3	MS	MM		
**6a**	22.3 **22.3**	22.3 **22.3**	22.3 **22.3**	11.1 **11.1**	89.1 **89.1**	89.1 **89.1**	89.1 **89.1**	89.1 **178**	22.3 –	22.3 –	>100	1.65
**6b**	22.3 **44.6**	44.6 **44.6**	44.6 **44.6**	22.3 **22.3**	89.1 **89.1**	178 178	178 178	89.1 **89.1**	22.3 –	22.3 –	65.5	1.99
**6c**	178 178	178 178	178 178	178 178	178 178	178 178	178 178	89.1 178	44.6 –	44.6 –	>100	1.18
**6d**	44.8 **44.8**	44.8 **44.8**	89.6 **89.6**	44.8 **44.8**	44.8 **44.8**	44.8 **44.8**	44.8 **44.8**	22.4 **44.8**	22.4 –	11.2 –	>100	2.98
**6e**	44.8 **44.8**	44.8 **44.8**	44.8 **44.8**	22.4 **22.4**	89.6 **89.6**	179 179	179 179	89.6 **89.6**	22.4 –	11.2 –	>100	2.47
**6f**	359 359	359 359	359 359	179 359	179 359	359 359	359 359	179 179	89.6 –	44.8 –	>100	2.29
**6g**	45.1 **45.1**	45.1 **45.1**	45.1 **45.1**	45.1 **45.1**	90.1 **90.1**	90.1 **90.1**	90.1 **90.1**	90.1 **90.1**	22.5 –	11.3 –	>100	2.11
**6h**	45.1 **45.1**	90.1 **90.1**	90.1 **90.1**	45.1 **45.1**	45.1 **45.1**	45.1 **45.1**	45.1 **45.1**	22.5 **22.5**	22.5 –	22.5 –	>100	1.74
**6i**	180 180	180 361	361 361	180 180	361 361	180 180	180 180	180 180	90.1 –	45.1 –	47.5	2.20
**6j**	9.83 **9.83**	4.91 **9.83**	9.83 **9.83**	2.46 **2.46**	19.7 **39.3**	19.7 **19.7**	78.6 **78.6**	39.3 **39.3**	9.83 –	2.50 –	37.7	3.41
**6k**	19.7 **19.7**	19.7 **19.7**	9.83 **19.7**	9.83 **9.83**	78.6 **78.6**	78.6 **78.6**	157 157	157 157	19.7 –	19.7 –	>100	3.48
**6l**	39.3 **39.3**	39.3 **78.6**	78.6 **78.6**	39.3 **39.3**	157 157	157 157	157 157	157 157	39.3 –	9.83 –	>100	3.10
**AMP**	5.72 5.72	–	–	–	2.81 2.81	11.5 11.5	11.5 11.5	11.5 11.5	–	–	–	–
**OXA**	1.25 1.25	79.8 79.8	29.7 29.7	9.9 9.9	–	–	–	–	–	–	–	–
**TTC**	4.52 NT	36.1 NT	36.1 NT	>72.1 NT	–	–	–	–	–	–	–	–
**CPX**	1.51 NT	48.3 NT	24.2 NT	24.2 NT	–	–	–	–	–	–	–	–
**INH**	–	–	–	–	–	–	–	–	117 –	467 –	–	–
**RIF**	–	–	–	–	–	–	–	–	19.4 –	2.43 –	–	–

SA = *Staphylococcus aureus* ATCC 29213; MRSA1–3 = clinical isolates of methicillin-resistant *S. aureus*SA 3202, SA 630 (National Institute of Public Health, Prague, Czech Republic) and 63718 (Department of Infectious Diseases and Microbiology, Faculty of Veterinary Medicine, University of Veterinary Sciences Brno, Czech Republic); EF = *Enterococcus faecalis* ATCC 29213 and vancomycin-resistant enterococci VRE1–3 = VRE 342B, VRE 368 and VRE 725B; MS = *M. smegmatis* ATCC 700084; MM = *Mycobacterium marinum* CAMP 5644. NT = not tested. The real bactericidal values required by the MBC/MIC ≤ 4 rule are in bold.

**Table 2:** Lowest MIC values with at least 70% inhibition of S. aureus ATCC 29213 respiratory activity.

No.	Conc.	*S. aureus* Respiration Inhibition [%]
**6a**	2× MIC (2× MBC)	97.7
**6e**	2× MIC (2× MBC)	97.4
**6g**	2× MIC (2× MBC)	96.8
**6j**	0.5× MIC (0.5× MBC)	91.3
**6k**	2× MIC (2× MBC)	97.6
**APM**	16× MIC (>16× MBC)	81.9
**CPX**	32× MIC (32× MBC)	96.1

## Data Availability

Crystal data for structure (**6i**) are available in the Cambridge Crystallographic Data Centre. CCDC deposition number 2178985. https://ccdc.cam.ac.uk/ (accessed on 14 August 2022).

## Supplementary Materials

The following are available online at https://www.mdpi.com/article/10.3390/ijms232315078/s1.
