# Supplementary material for: Design, Synthesis and Antimicrobial Properties of New Tetracyclic Quinobenzothiazine Derivatives
Source: Int J Mol Sci. 2022 Dec 1;23(23):15078. doi: 10.3390/ijms232315078 (PMC9736374; doi:10.3390/ijms232315078)
Supplement: Supplementary file 1 [file ijms-23-15078-s001.zip › ijms-2045785-supplementary.pdf]

**Supplementary Materials**

# Design, Synthesis and Antimicrobial Properties of New Tetracyclic Quinobenzothiazine Derivatives

Ewa Kisiel-Nawrot [1], Dominika Pindjakova [2], Malgorzata Latocha [3],Andrzej Bak [4], Violetta Kozik [4], Kinga Suwinska [5], Aleksander Sochanik [6], Alois Cizek [7], Josef Jampilek [2,8] and Andrzej Zięba [1,*]

1 Department of Organic Chemistry, Faculty of Pharmaceutical Sciences in Sosnowiec, Medical University of Silesia, Jagiellońska 4, 41-200 Sosnowiec, Poland

2 Department of Analytical Chemistry, Faculty of Natural Sciences, Comenius University, Ilkovicova 6, 842 15 Bratislava, Slovakia

3 Department of Cell Biology, Faculty of Pharmaceutical Sciences in Sosnowiec, Medical University of Silesia, Jedności 9, 41-200 Sosnowiec, Poland

4 Institute of Chemistry, University of Silesia, Szkolna 9, 40-007 Katowice, Poland

5 Faculty of Mathematics and Natural Sciences, Cardinal Stefan Wyszyński University, K. Woycickiego 1/3, 01-938 Warszawa, Poland

6 Center for Translational Research and Molecular Biology of Cancer, Maria Skłodowska-Curie National Research Institute of Oncology, Wybrzeże AK 15, 44-101 Gliwice, Poland

7 Department of Infectious Diseases and Microbiology, Faculty of Veterinary Medicine, University of Veterinary Sciences Brno, Palackeho 1946/1, 612 42 Brno, Czech Republic

8 Department of Chemical Biology, Faculty of Science, Palacky University Olomouc, Slechtitelu 27, 783 71 Olomouc, Czech Republic

* Correspondence: zieba@sum.edu.pl

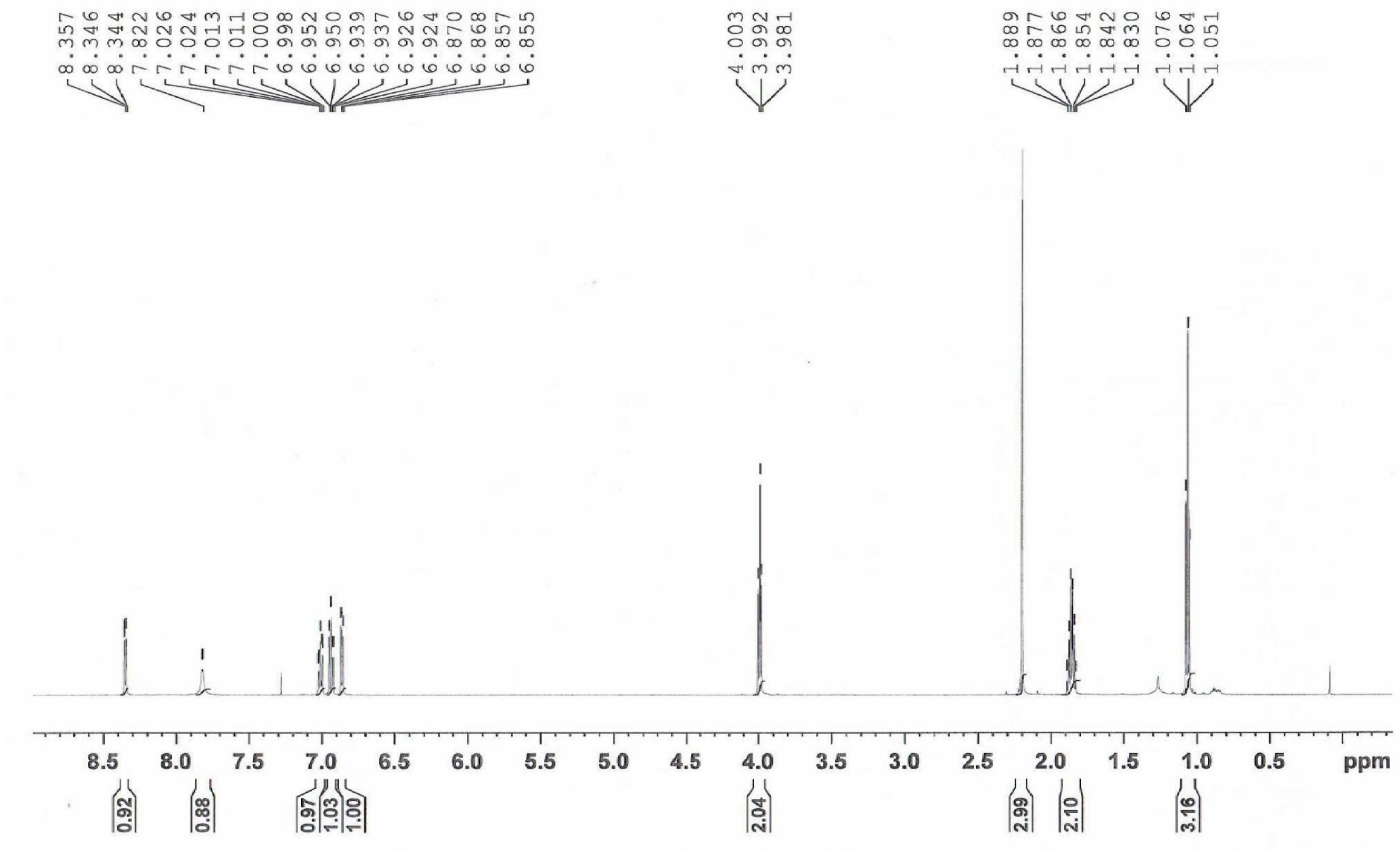


$^{1}$H NMR spectrum of 2-(propoxy)acetanilide **2a** in $CDCl_3$

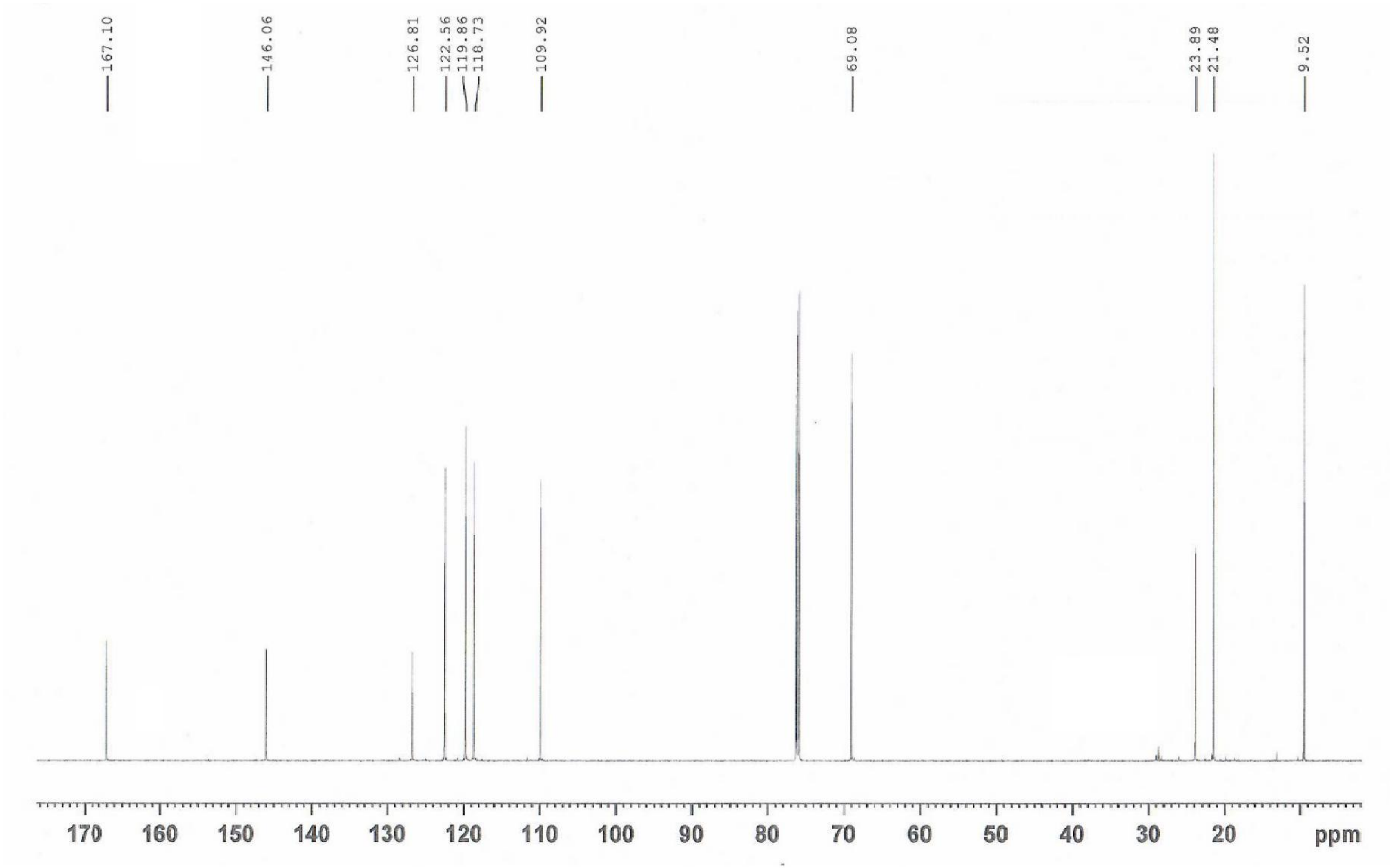


$^{13}C$ NMR spectrum of 2-(propoxy)acetanilide **2a** in $CDCl_3$

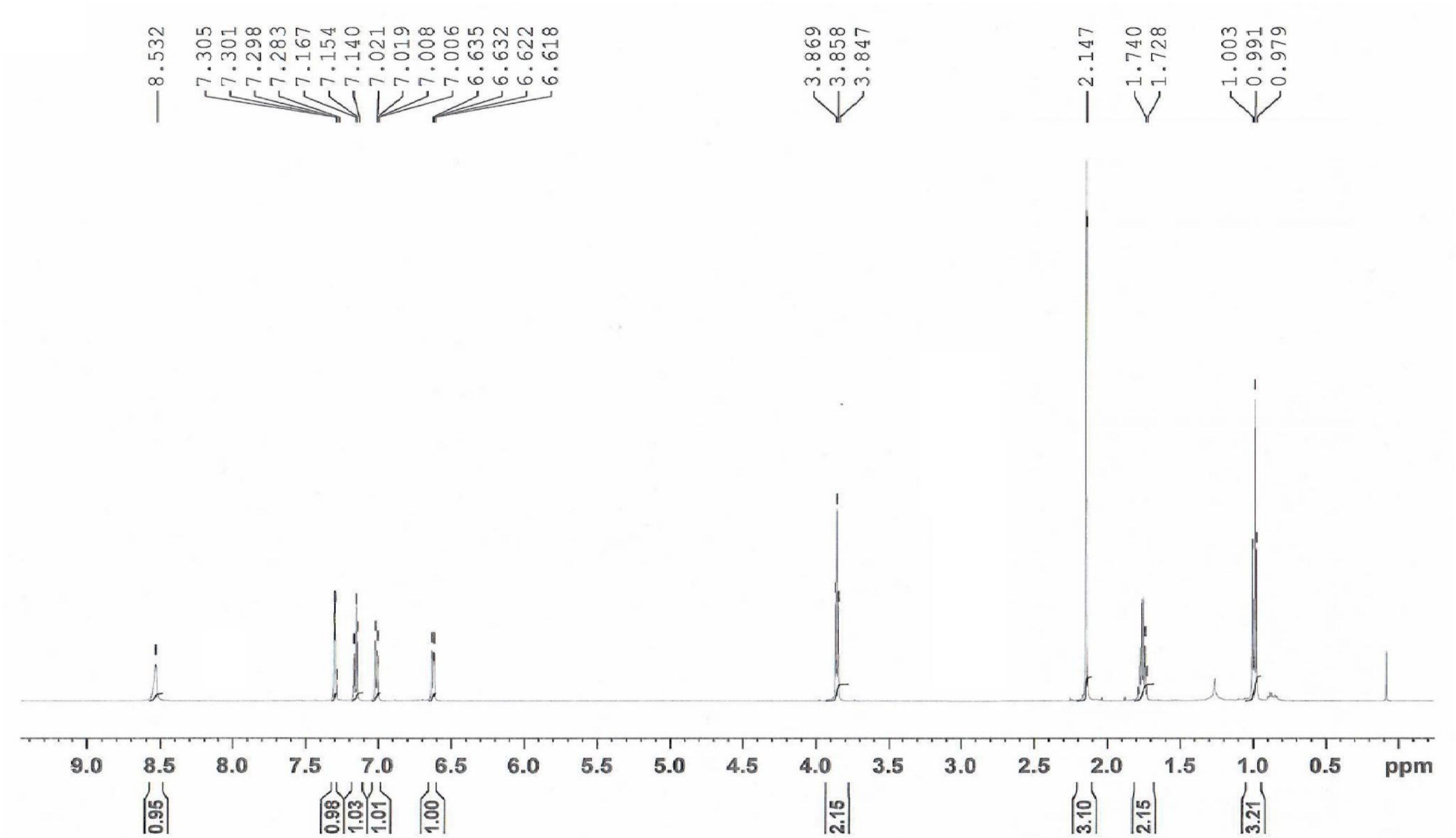


$^{1}$H NMR spectrum of 3-(propoxy)acetanilide **2b** in $CDCl_3$

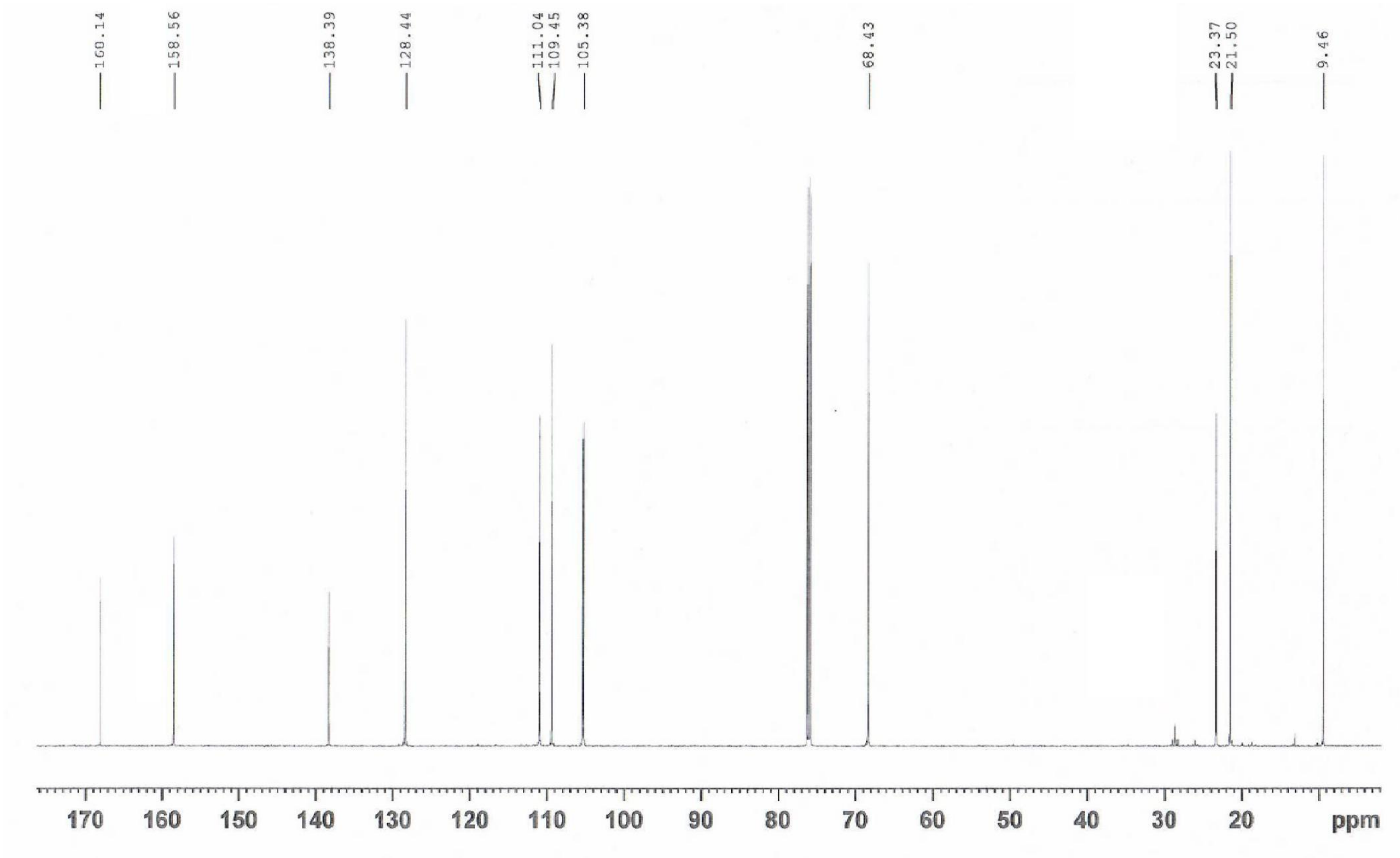


$^{13}$C NMR spectrum of 3-(propoxy)acetanilide 2**b** in $CDCl_3$

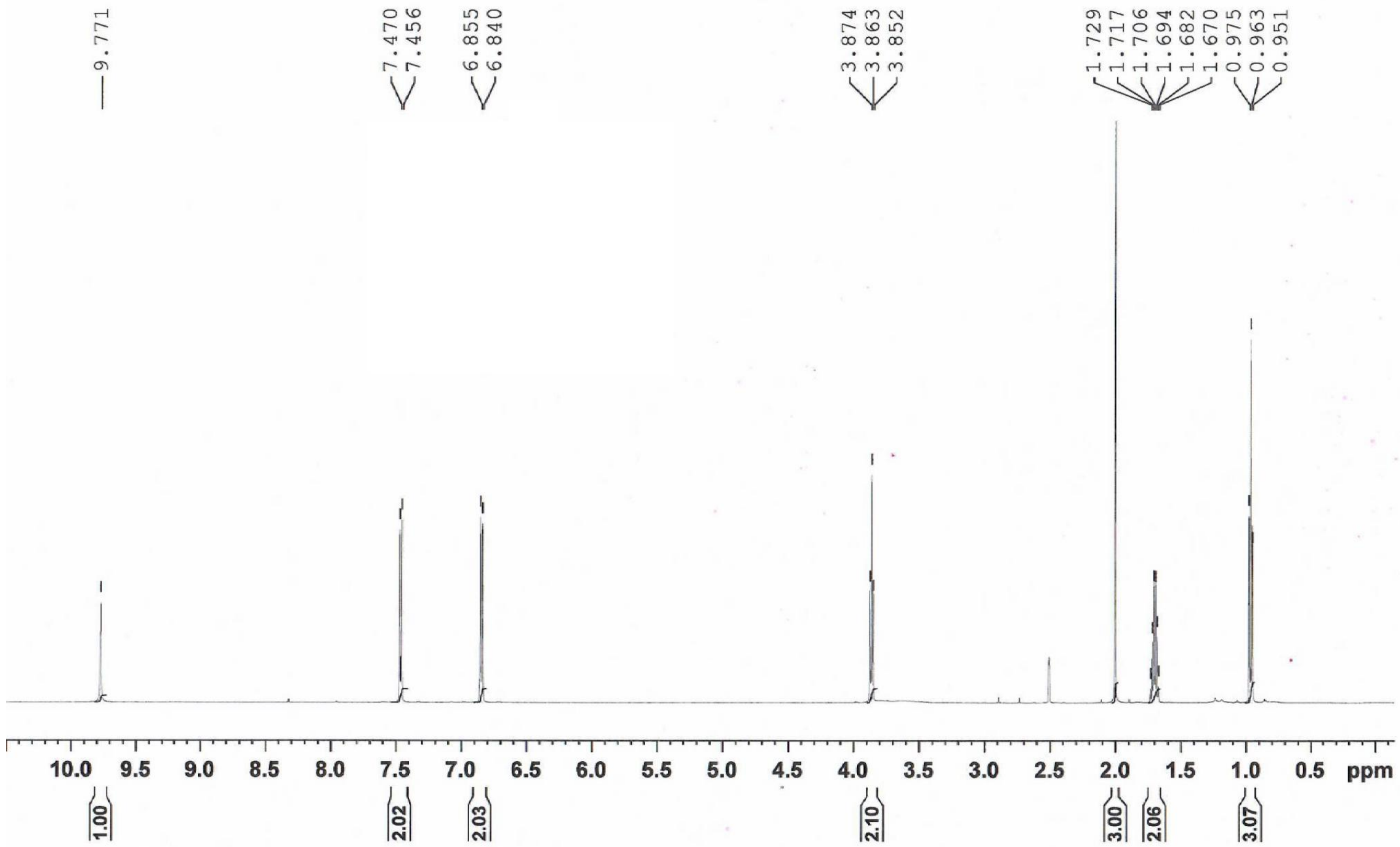


$^{1}$H NMR spectrum of 4-(propoxy)acetanilide **2c** in DMSO

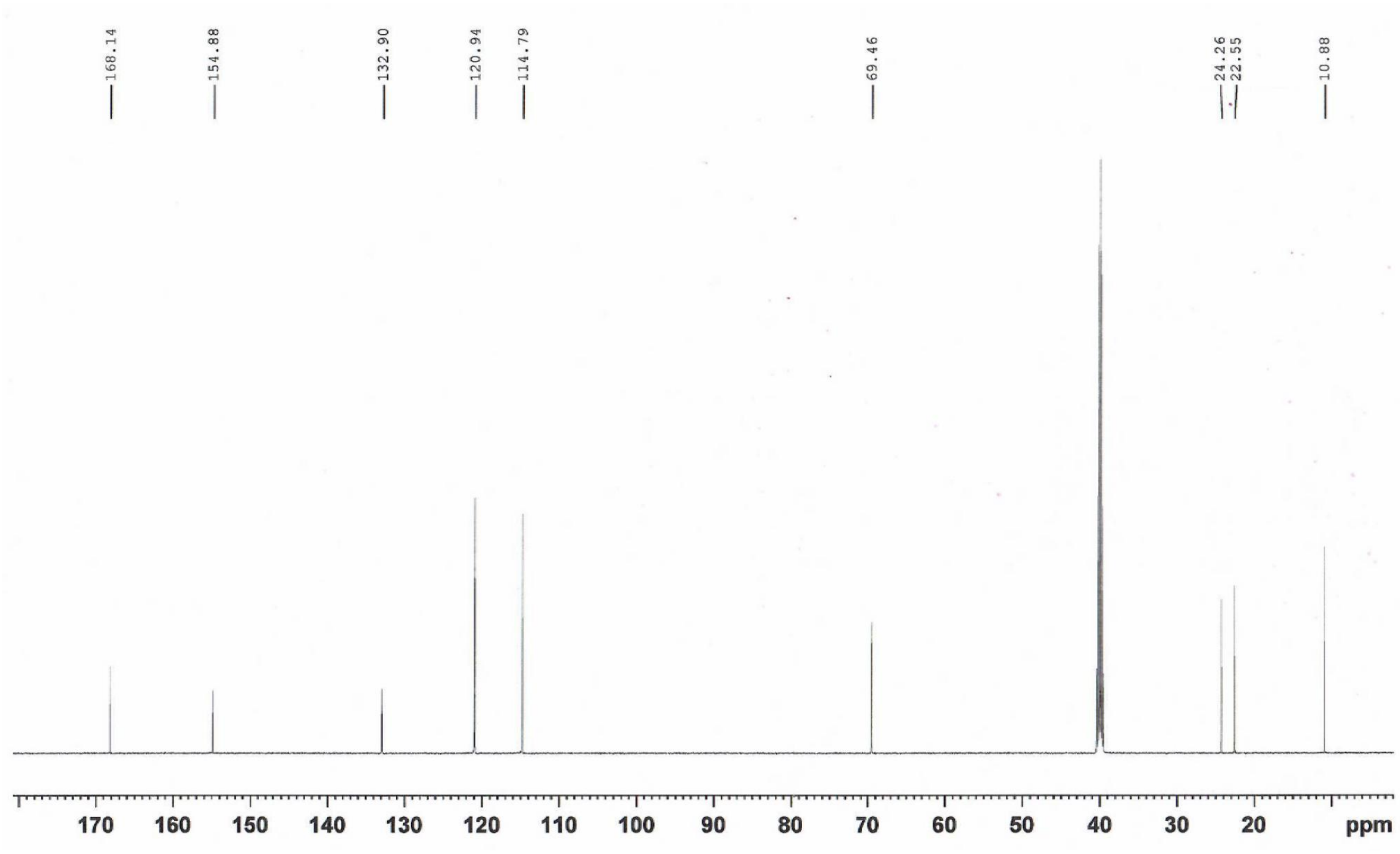


$^{13}$C NMR spectrum of 4-(propoxy)acetanilide **2c** in DMSO

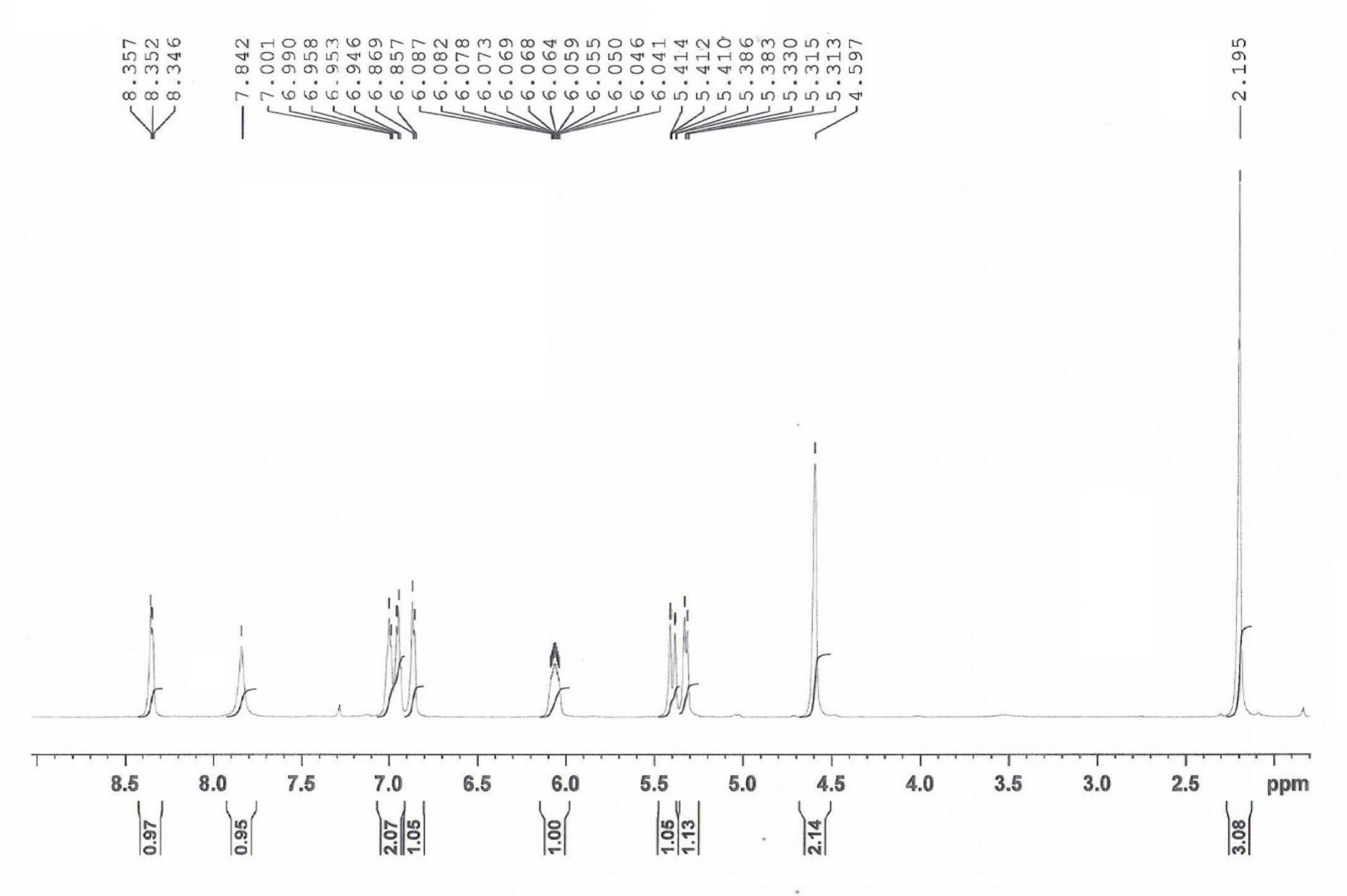


$^{1}$H NMR spectrum of 2-(allyloxy)acetanilide **2d** in $CDCl_3$

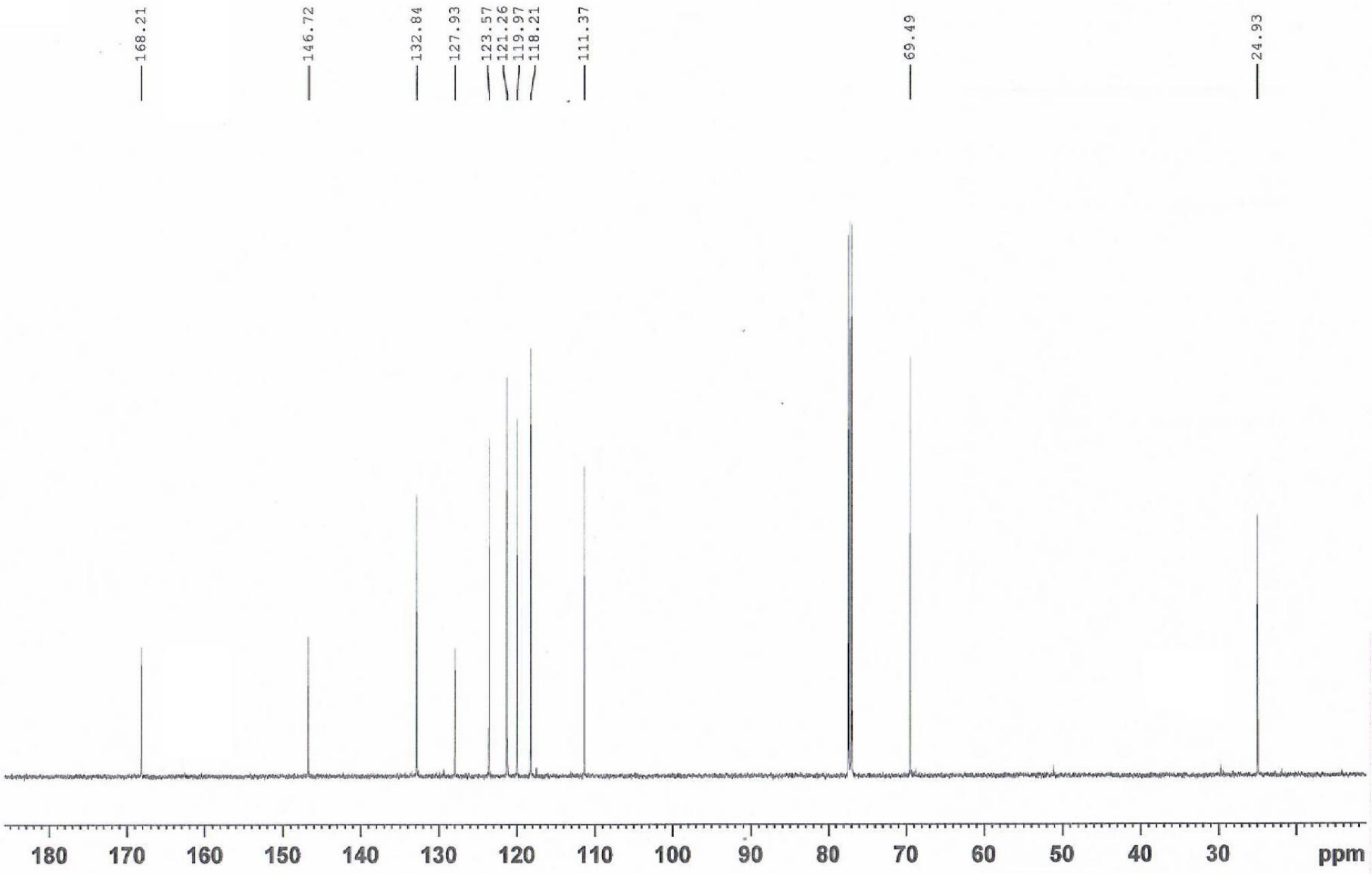


$^{13}C$ NMR spectrum of 2-(allyloxy)acetanilide **2d** in $CDCl_3$

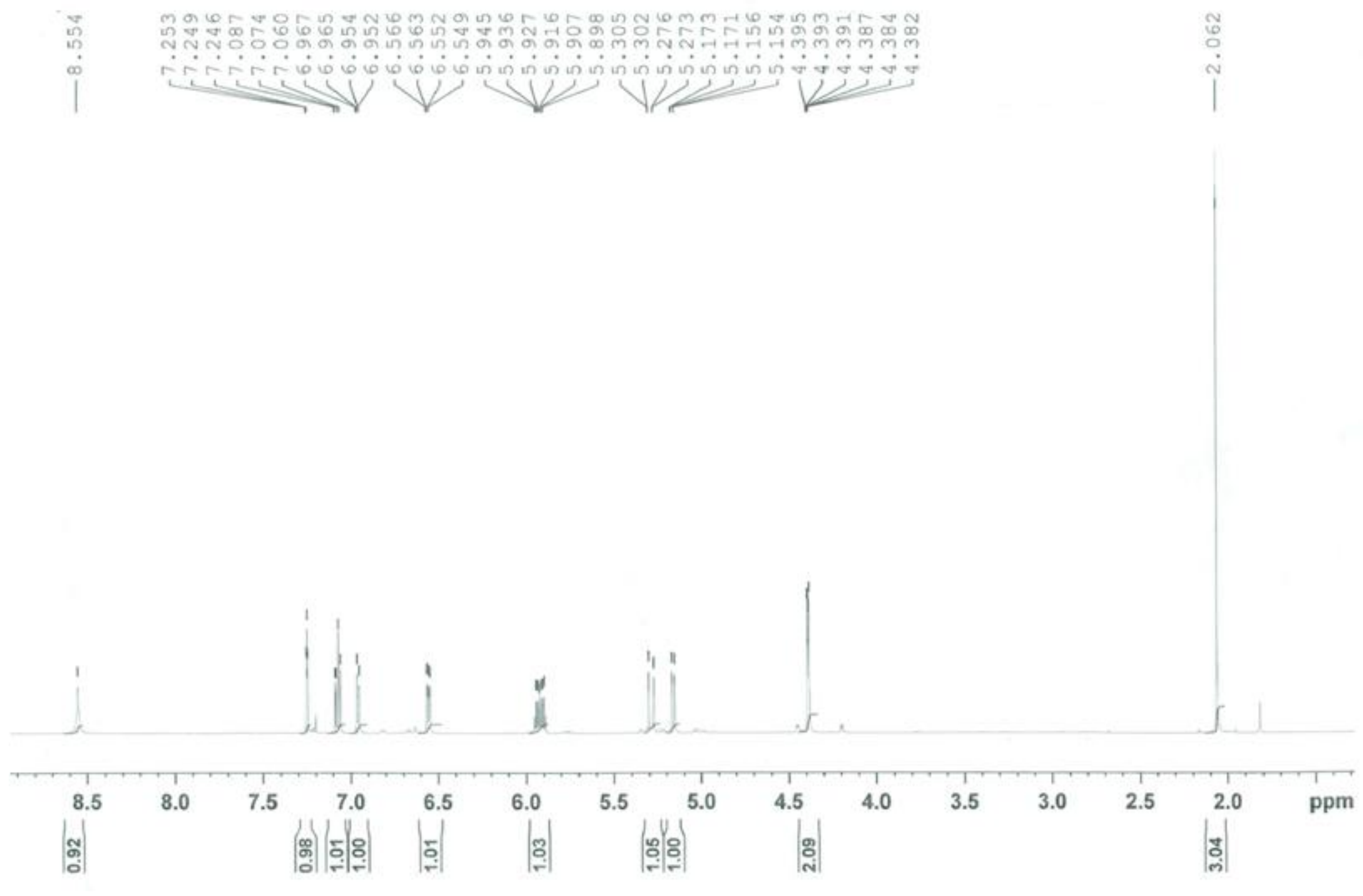


$^{1}$H NMR spectrum of 3-(allyloxy)acetanilide **2e** in $CDCl_3$

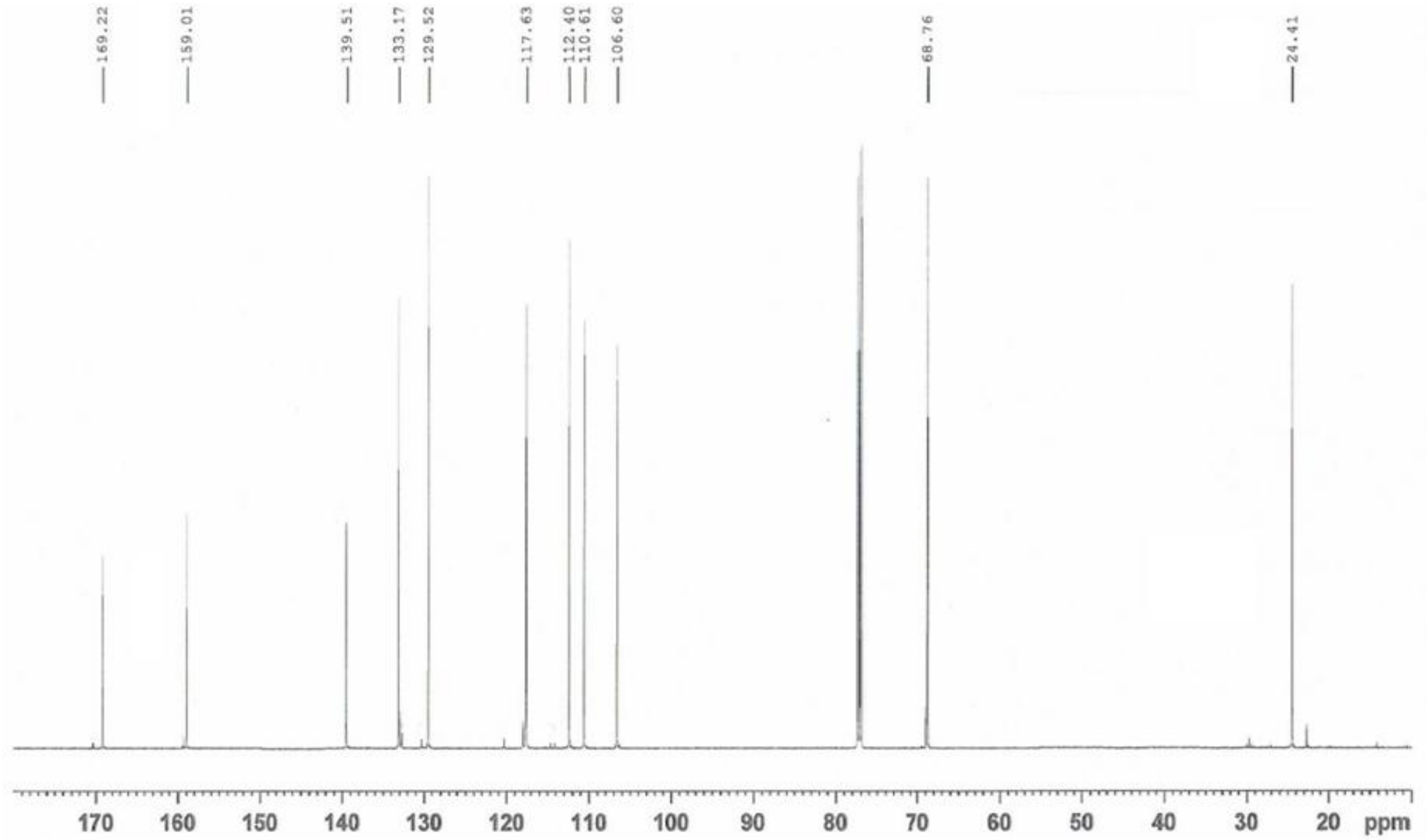


$^{13}$C NMR spectrum of 3-(allyloxy)acetanilide **2e** in $CDCl_3$

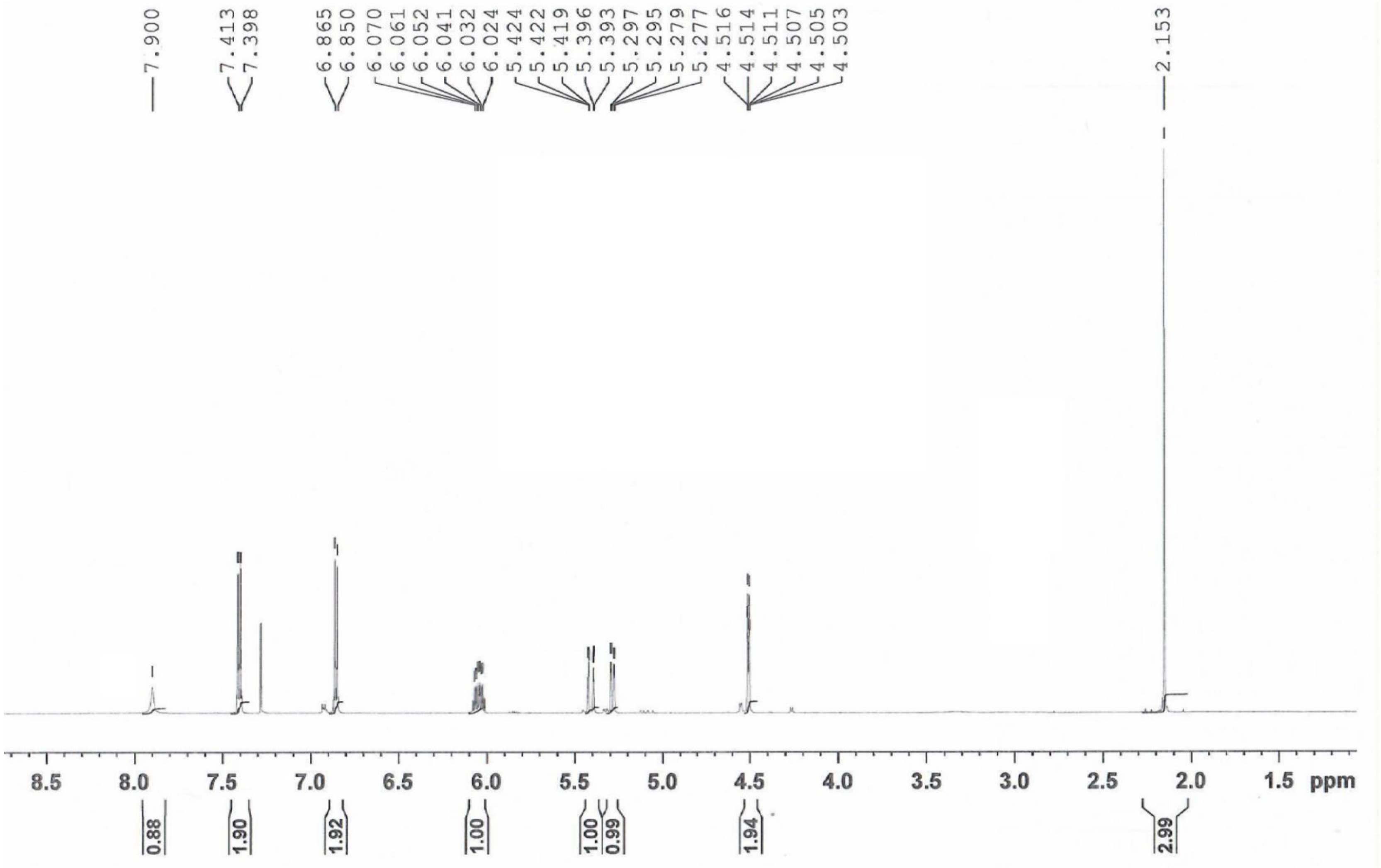


$^{1}H$ NMR spectrum of 4-(allyloxy)acetanilide **2f** in $CDCl_3$

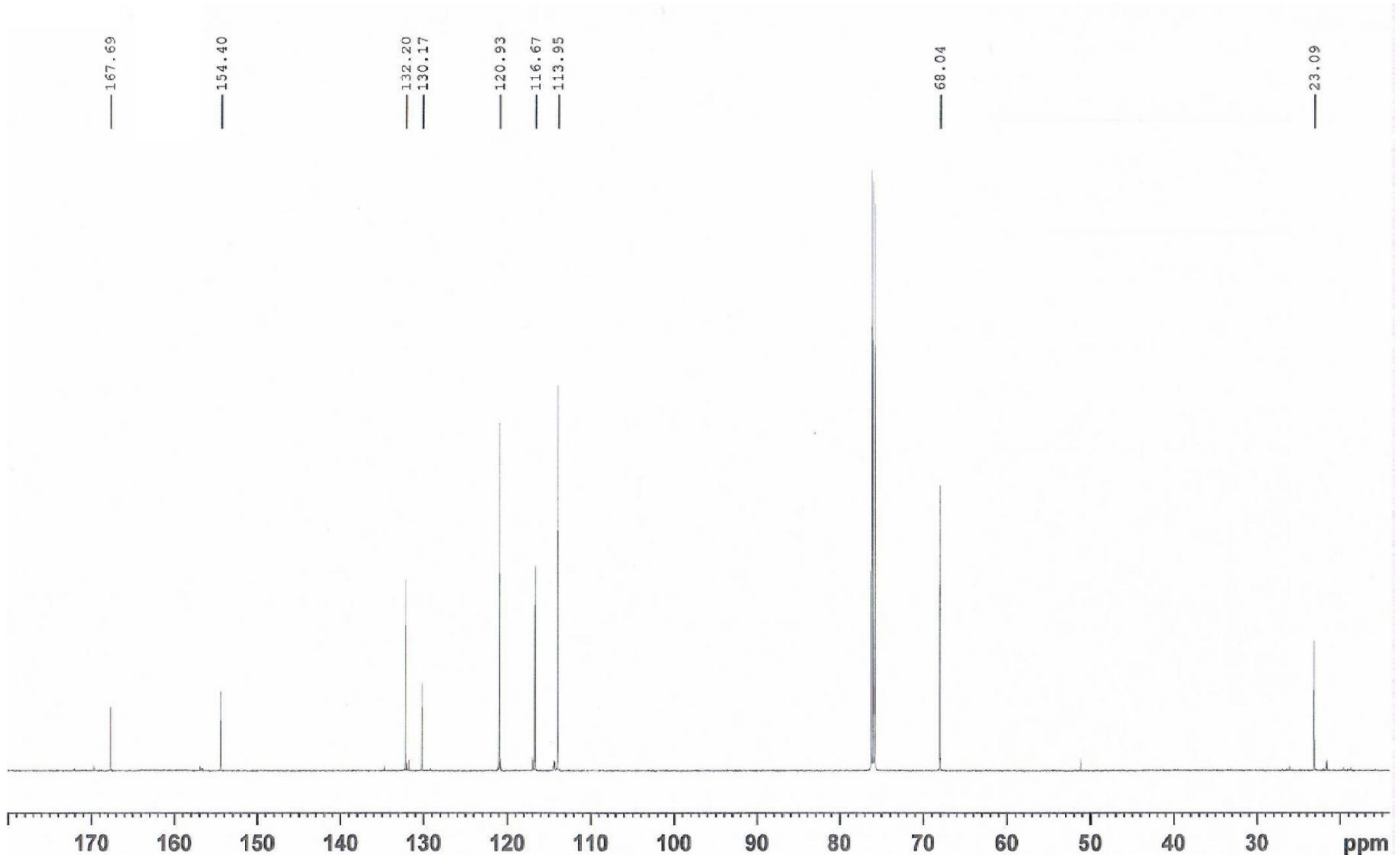


$^{13}C$ NMR spectrum of 4-(allyloxy)acetanilide **2f** in $CDCl_3$

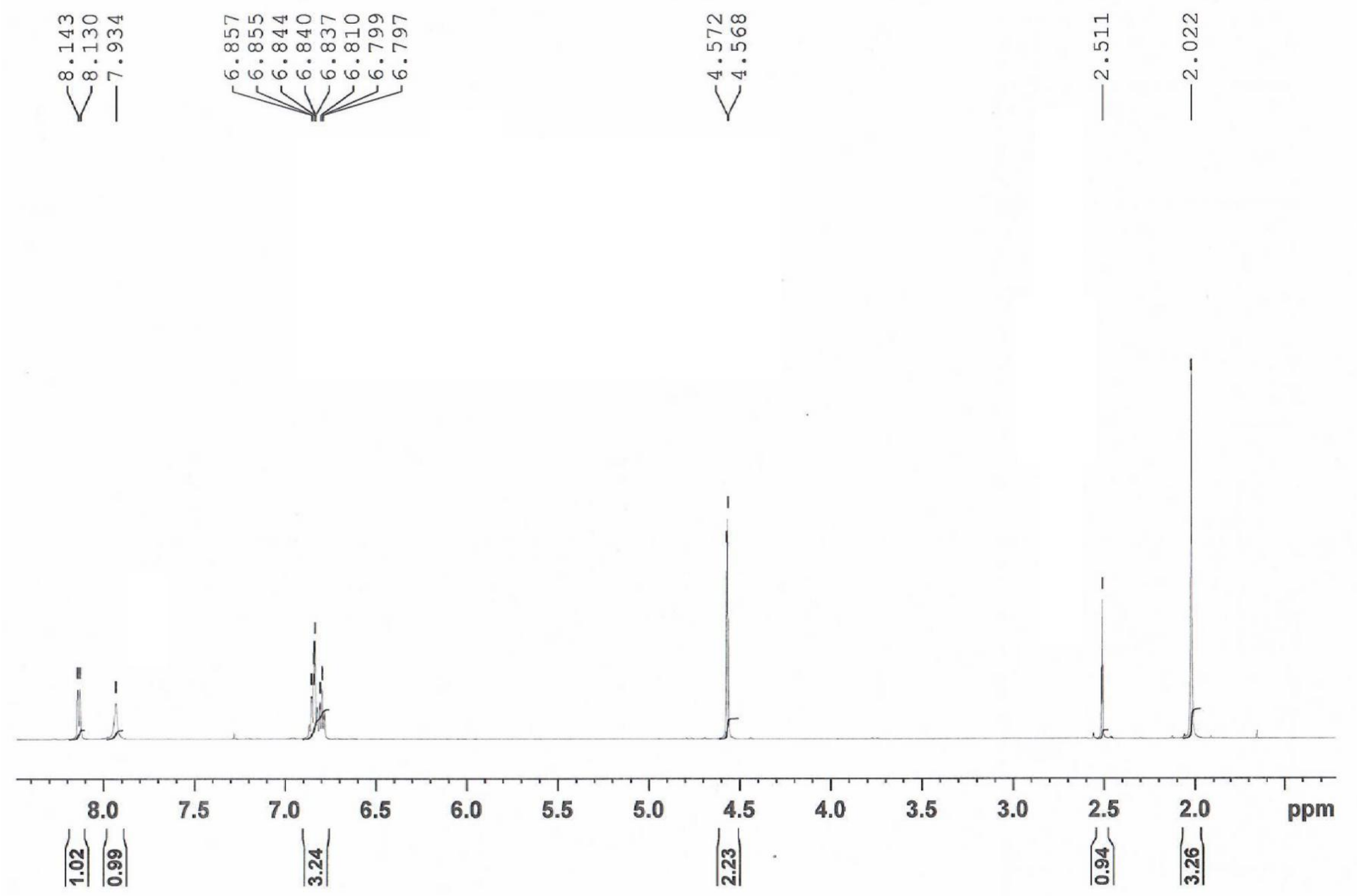


$^{1}$H NMR spectrum of 2-(propargyloxy)acetanilide **2g** in $CDCl_3$

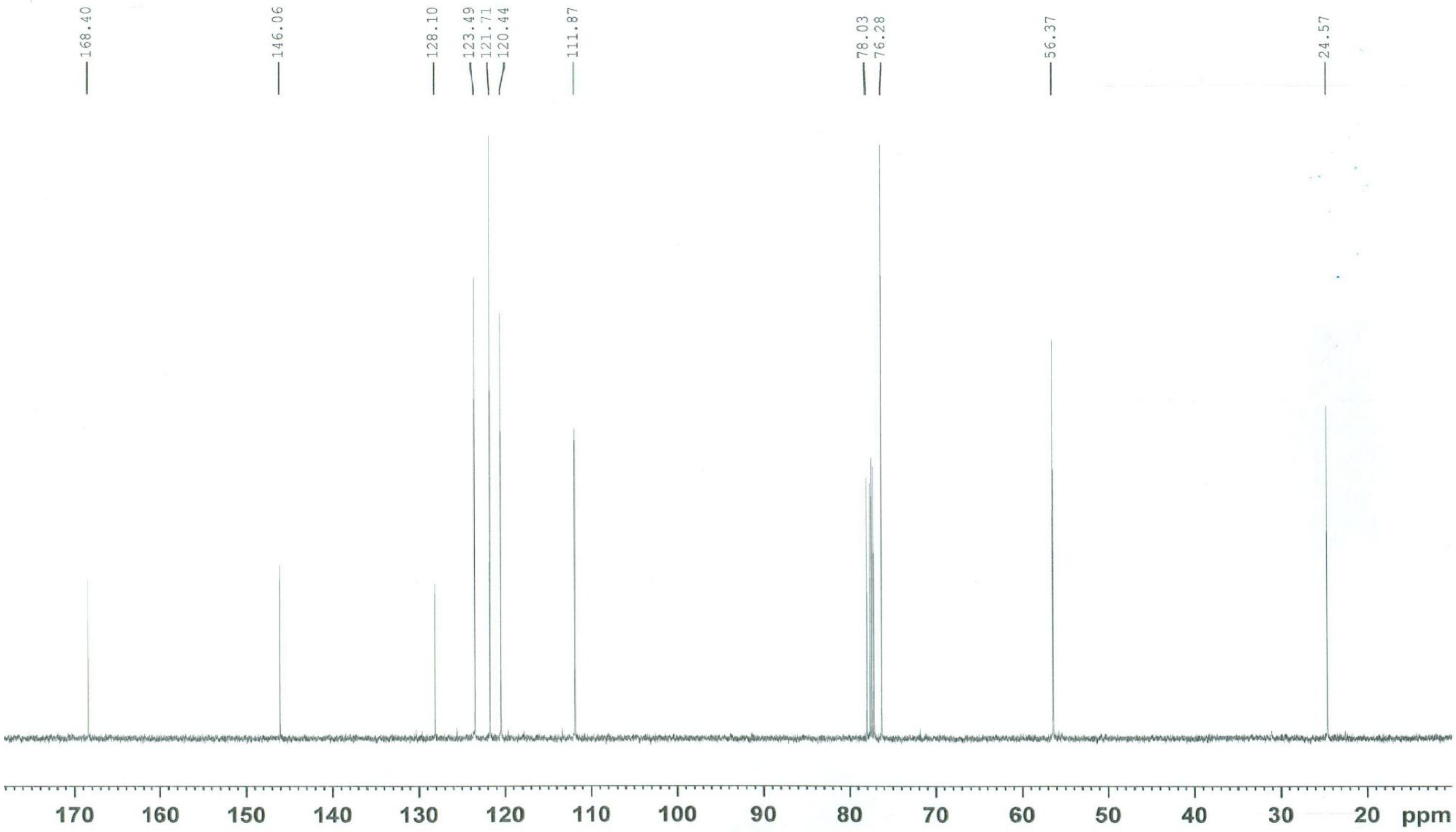


$^{13}C$ NMR spectrum of 2-(propargyloxy)acetanilide **2g** in $CDCl_3$

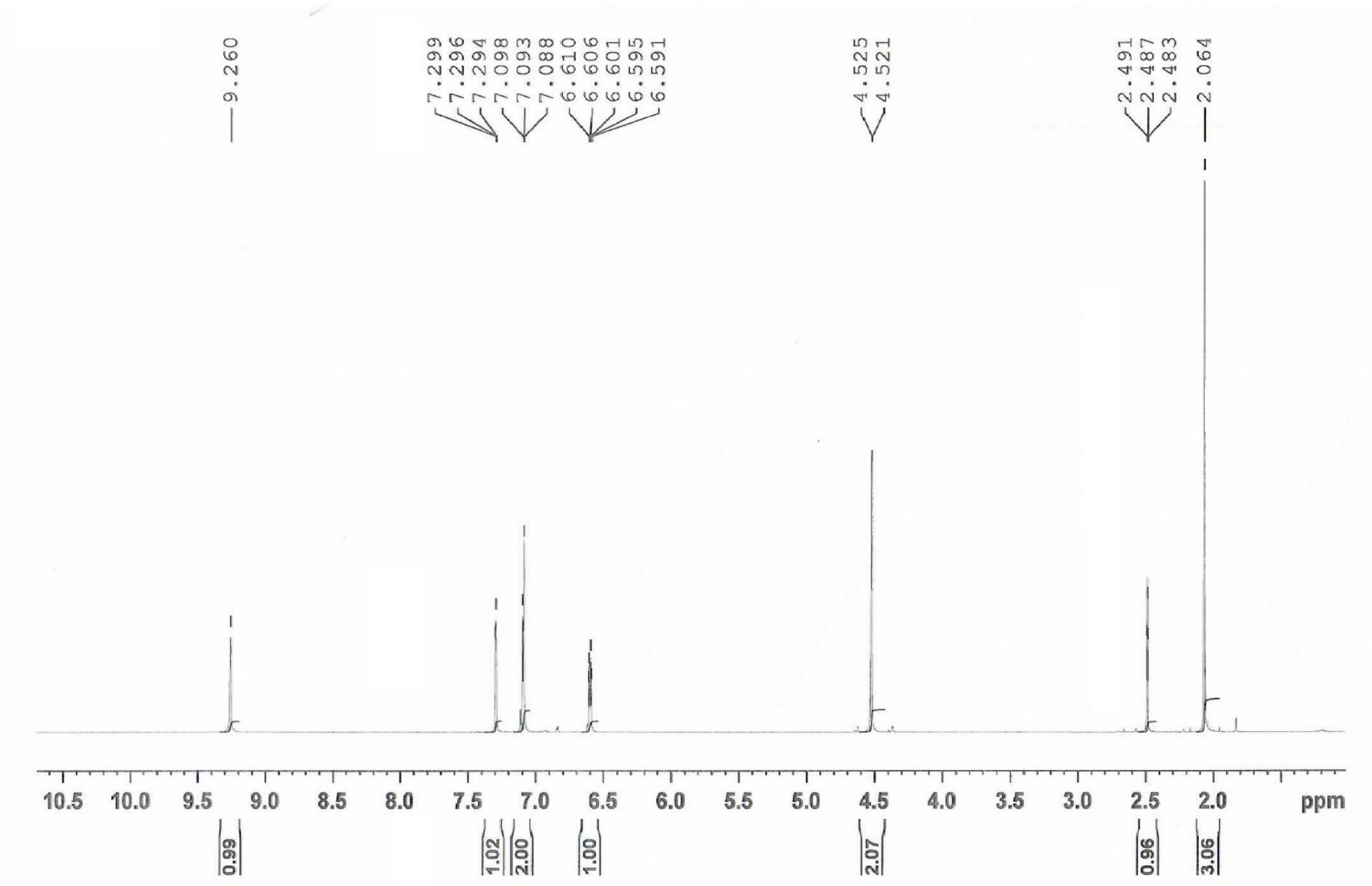


$^{1}H$ NMR spectrum of 3-(propargyloxy)acetanilide **2h** in $CDCl_3$

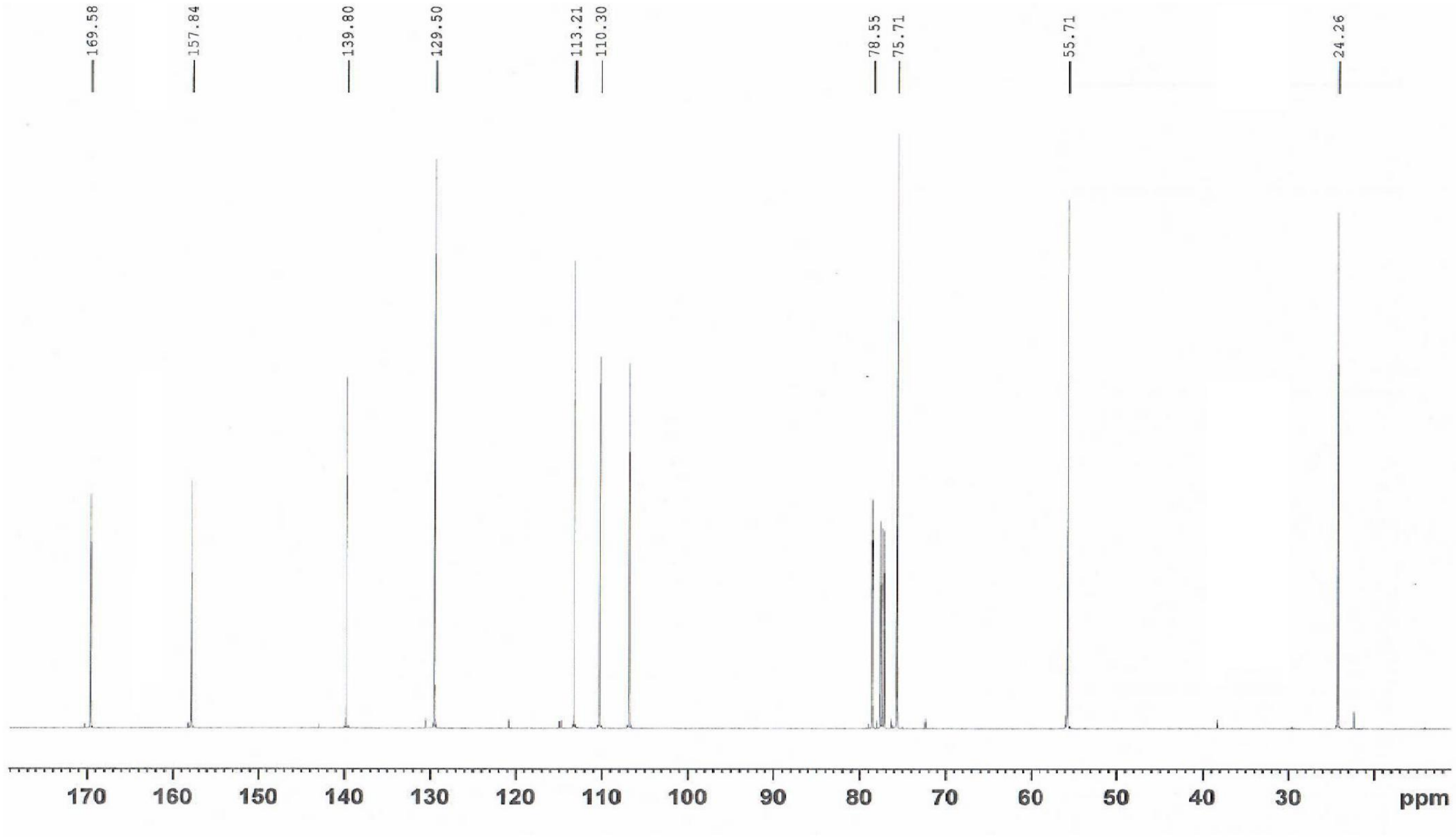


$^{13}C$ NMR spectrum of 3-(propargyloxy)acetanilide **2h** in $CDCl_3$

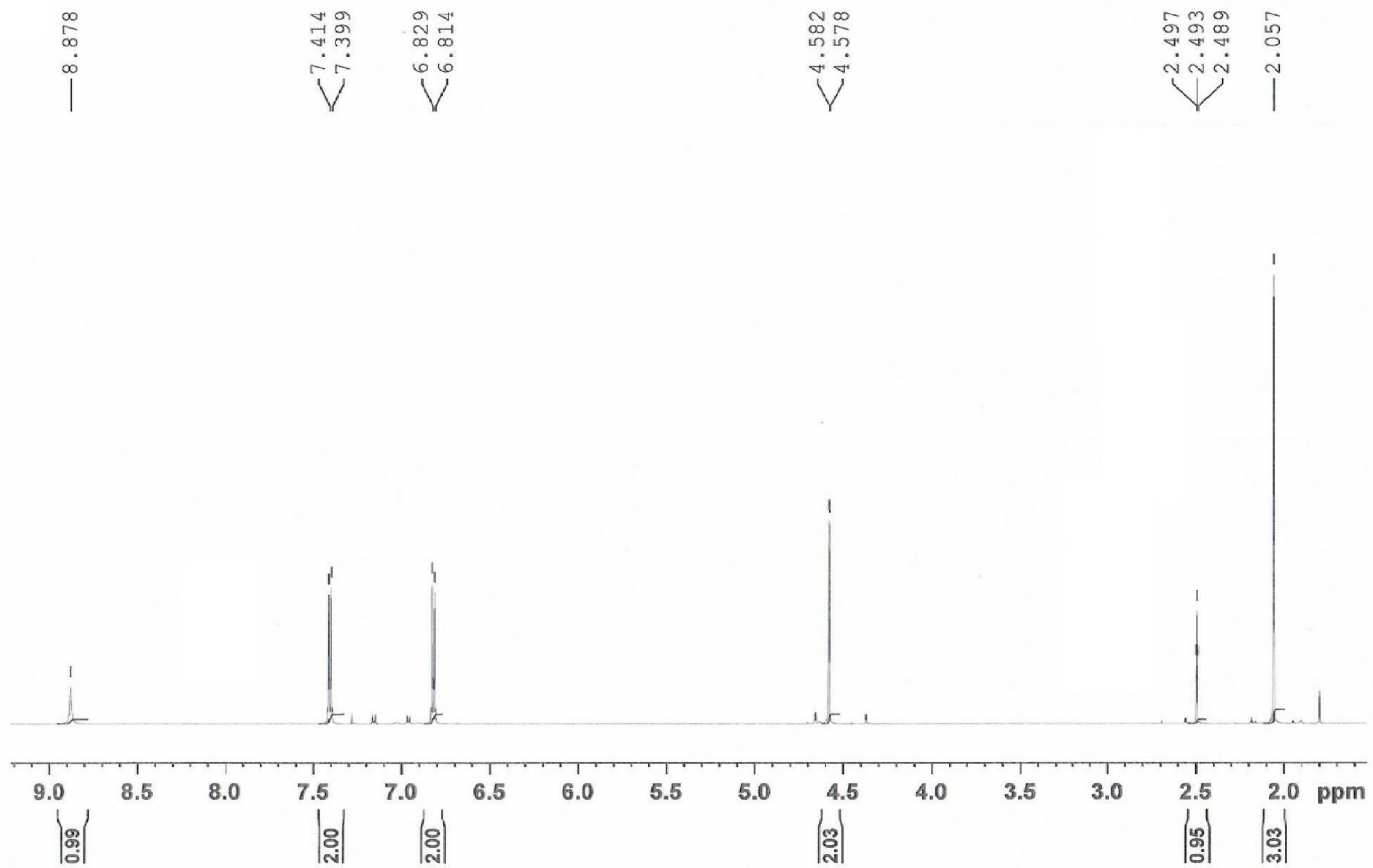


$^{1}$H NMR spectrum of 4-(propargyloxy)acetanilide **2i** in $CDCl_3$

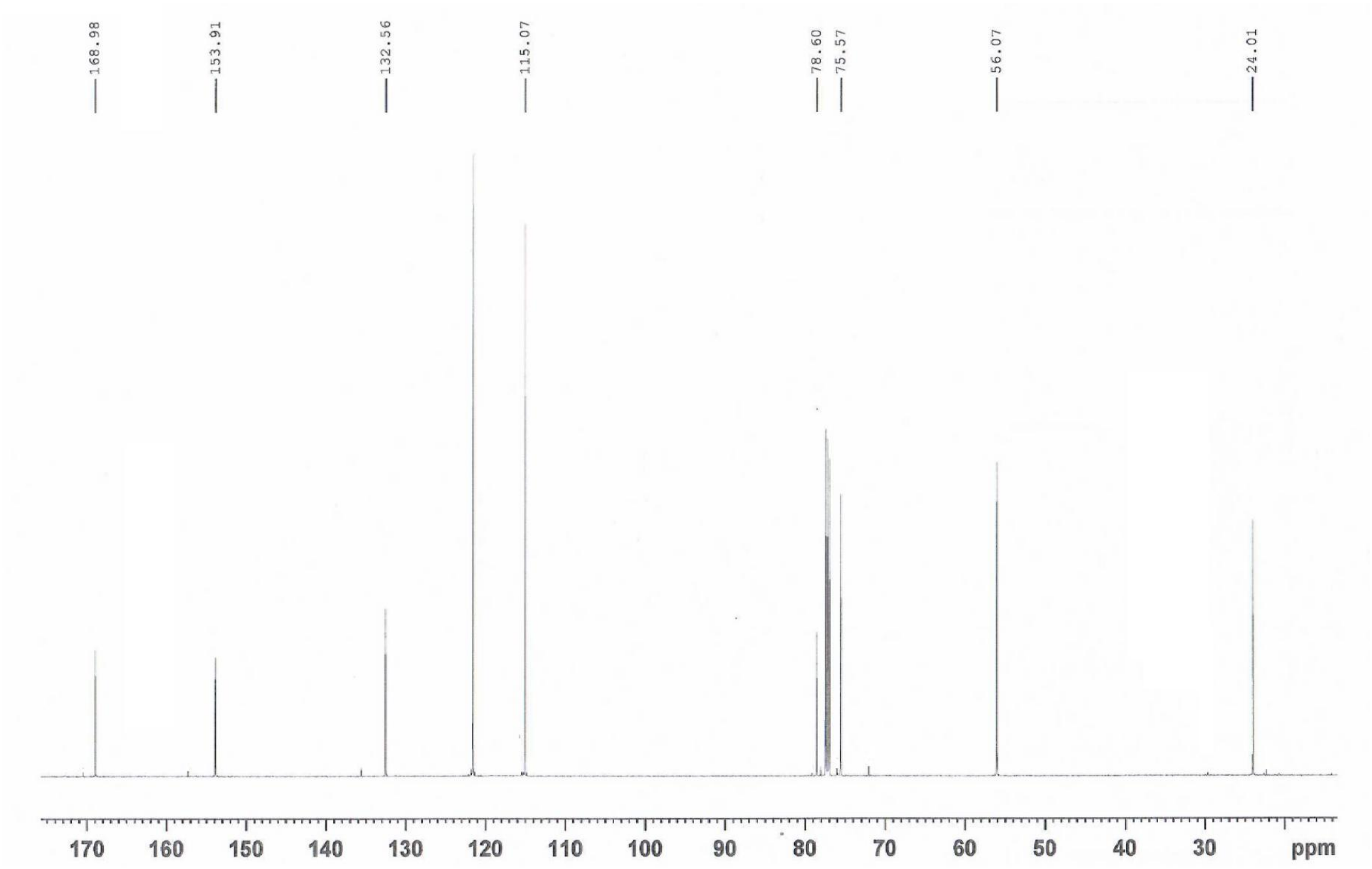


$^{13}C$ NMR spectrum of 4-(propargyloxy)acetanilide **2i** in $CDCl_3$

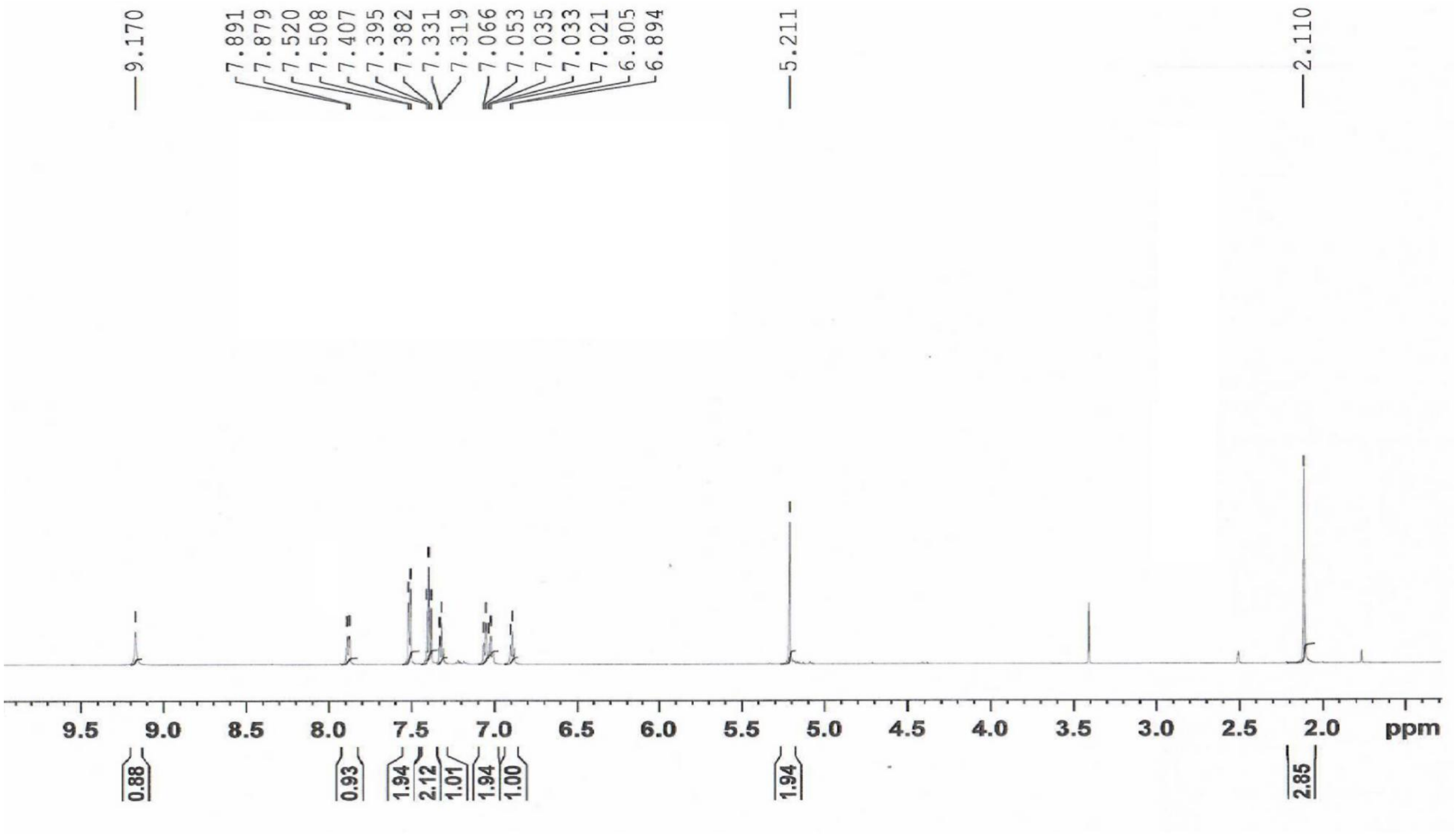


$^{1}$H NMR spectrum of 2-(benzyloxy)acetanilide **2j** in DMSO

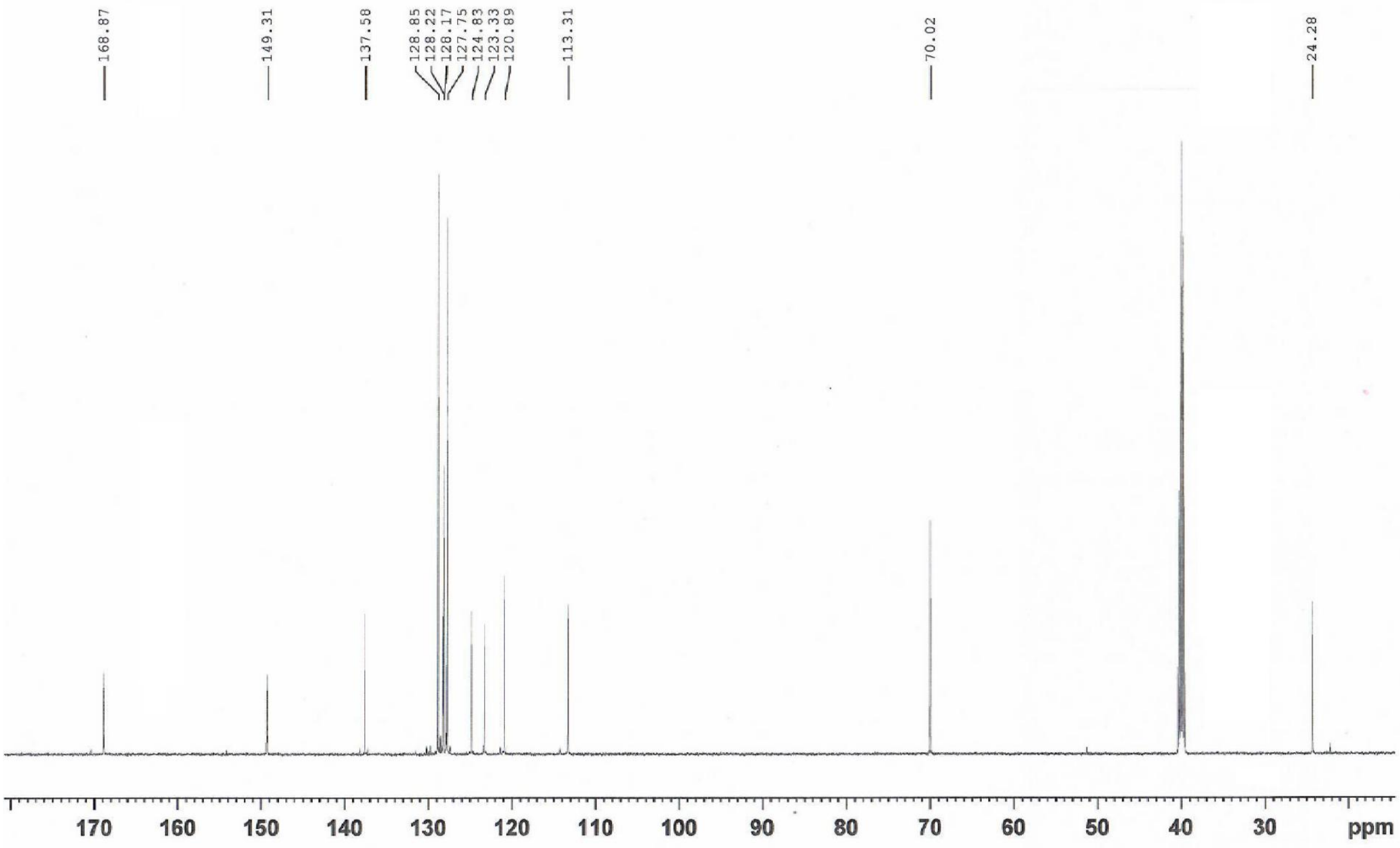


$^{13}$C NMR spectrum of 2-(benzyloxy)acetanilide **2j** in DMSO

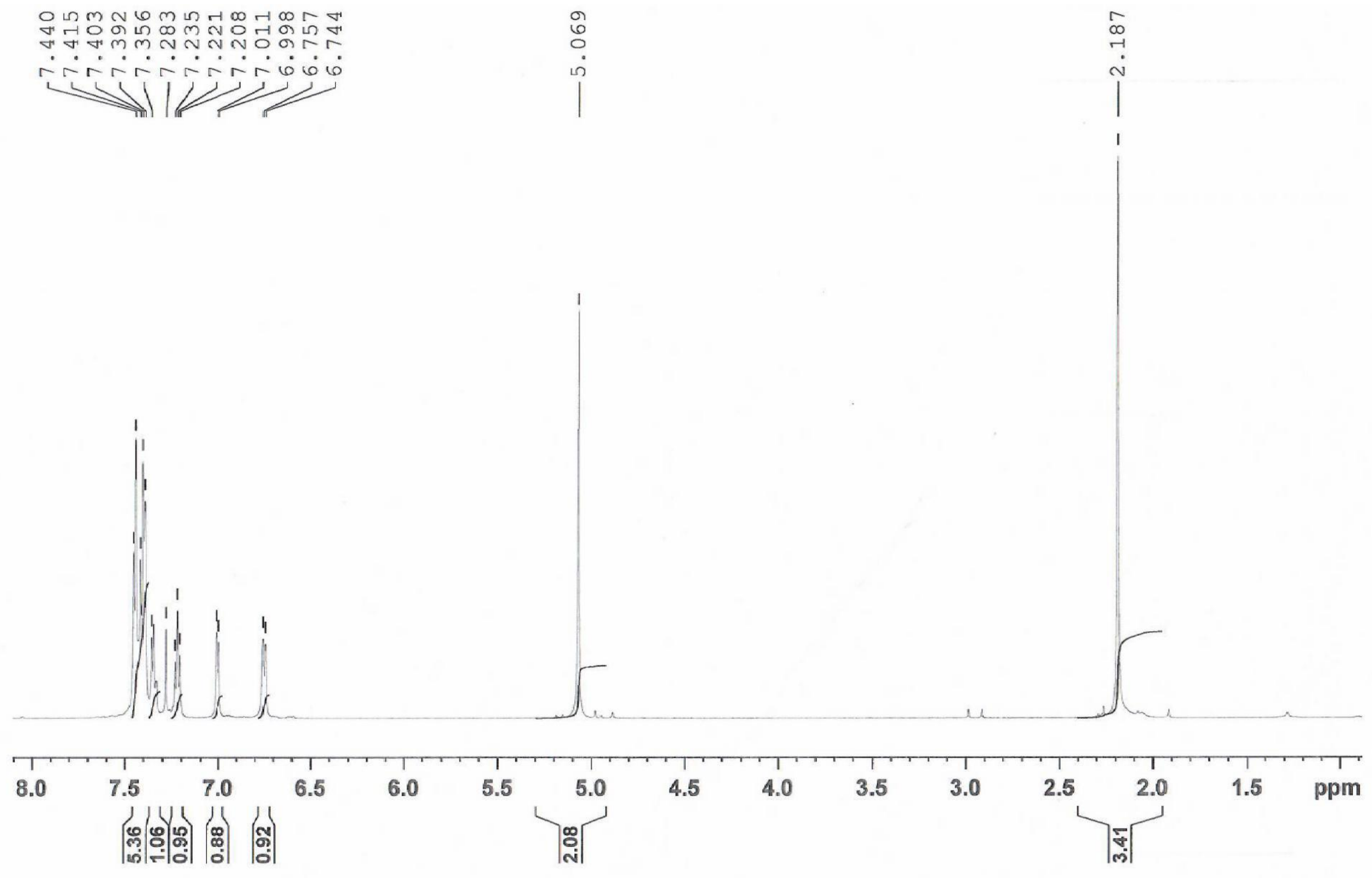


$^{1}$H NMR spectrum of 3-(benzyloxy)acetanilide **2k** in $CDCl_3$

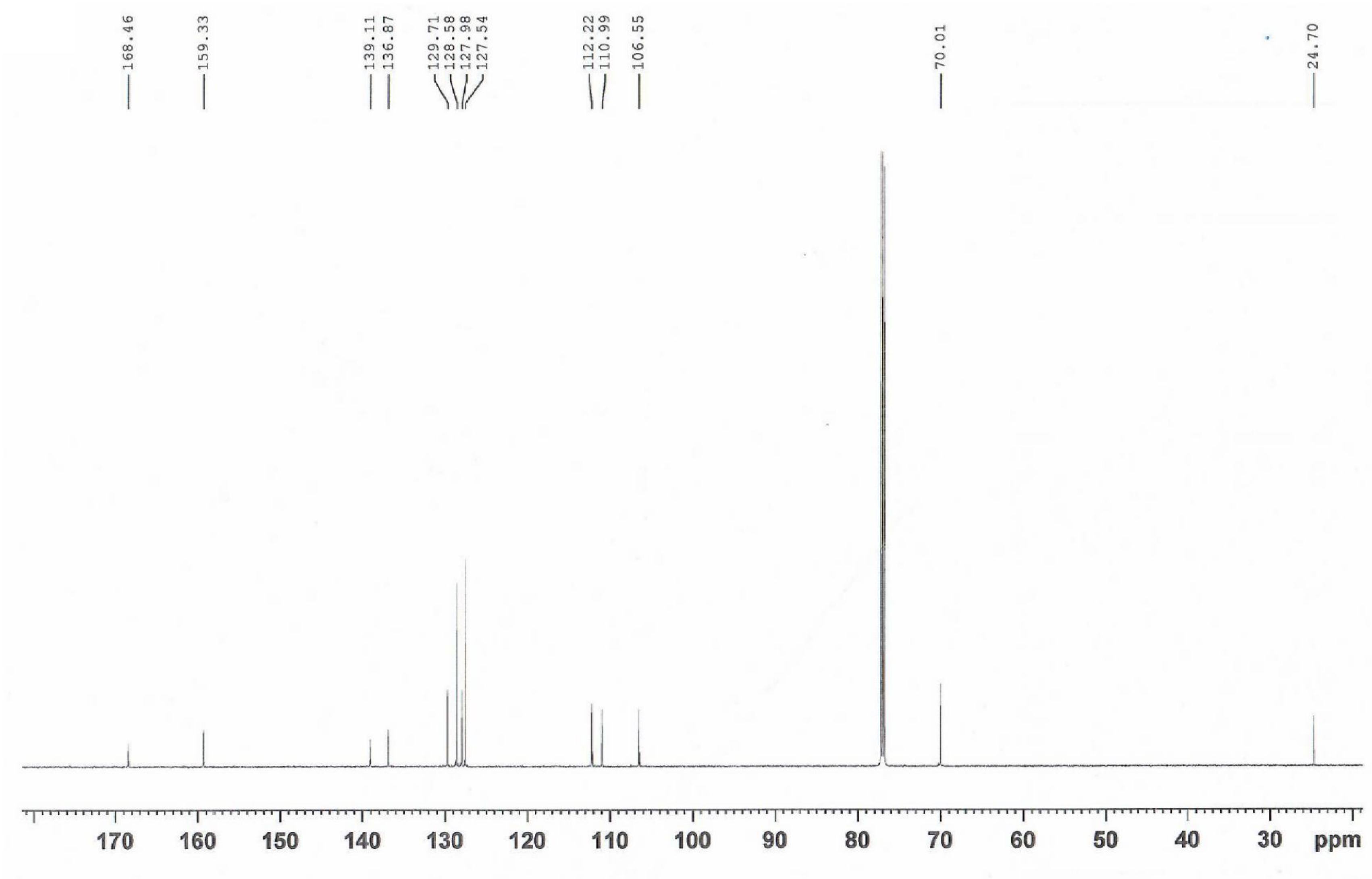


$^{13}C$ NMR spectrum of 3-(benzyloxy)acetanilide **2k** in $CDCl_3$

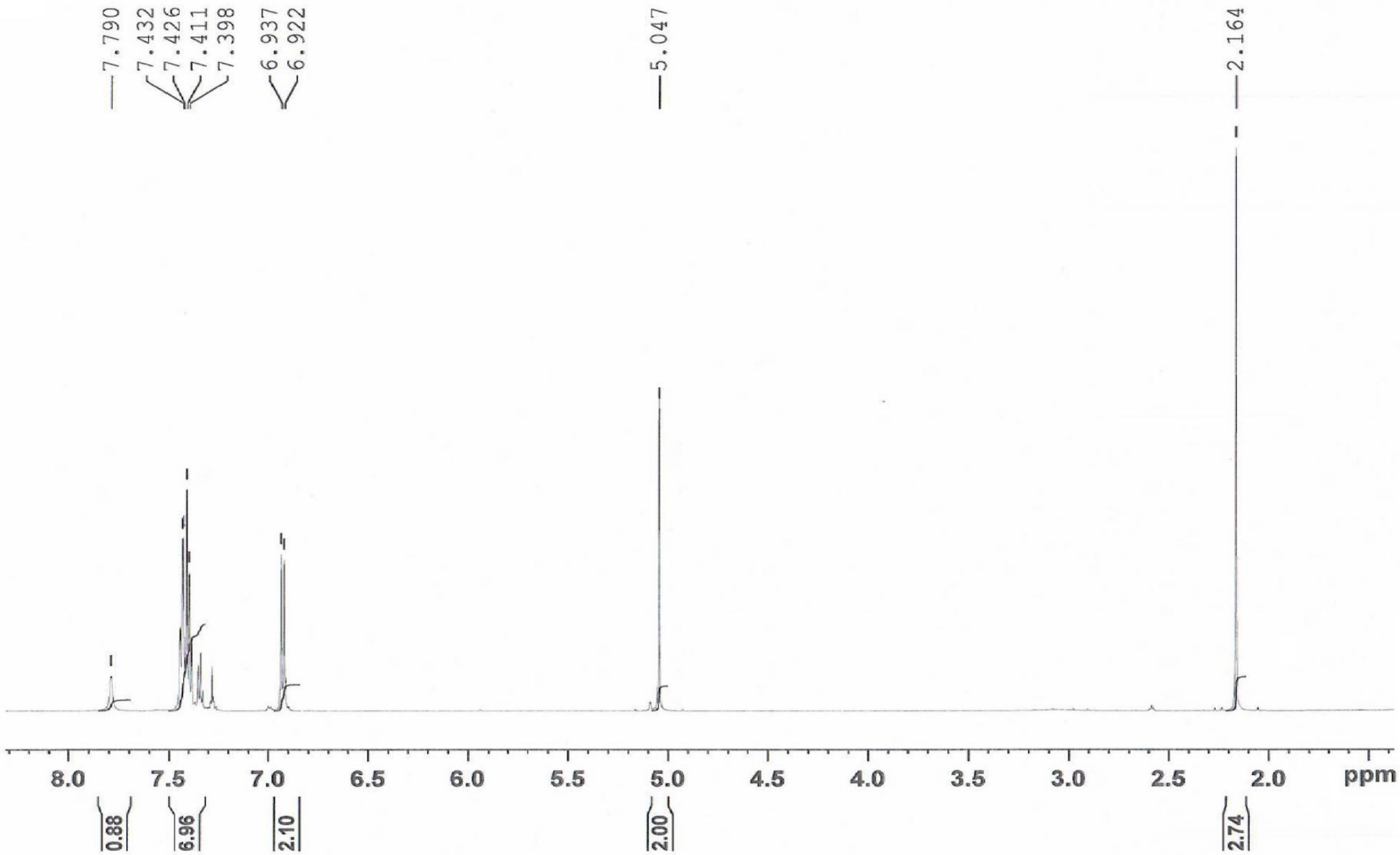


$^{1}$H NMR spectrum of 4-(benzyloxy)acetanilide **2l** in $CDCl_3$

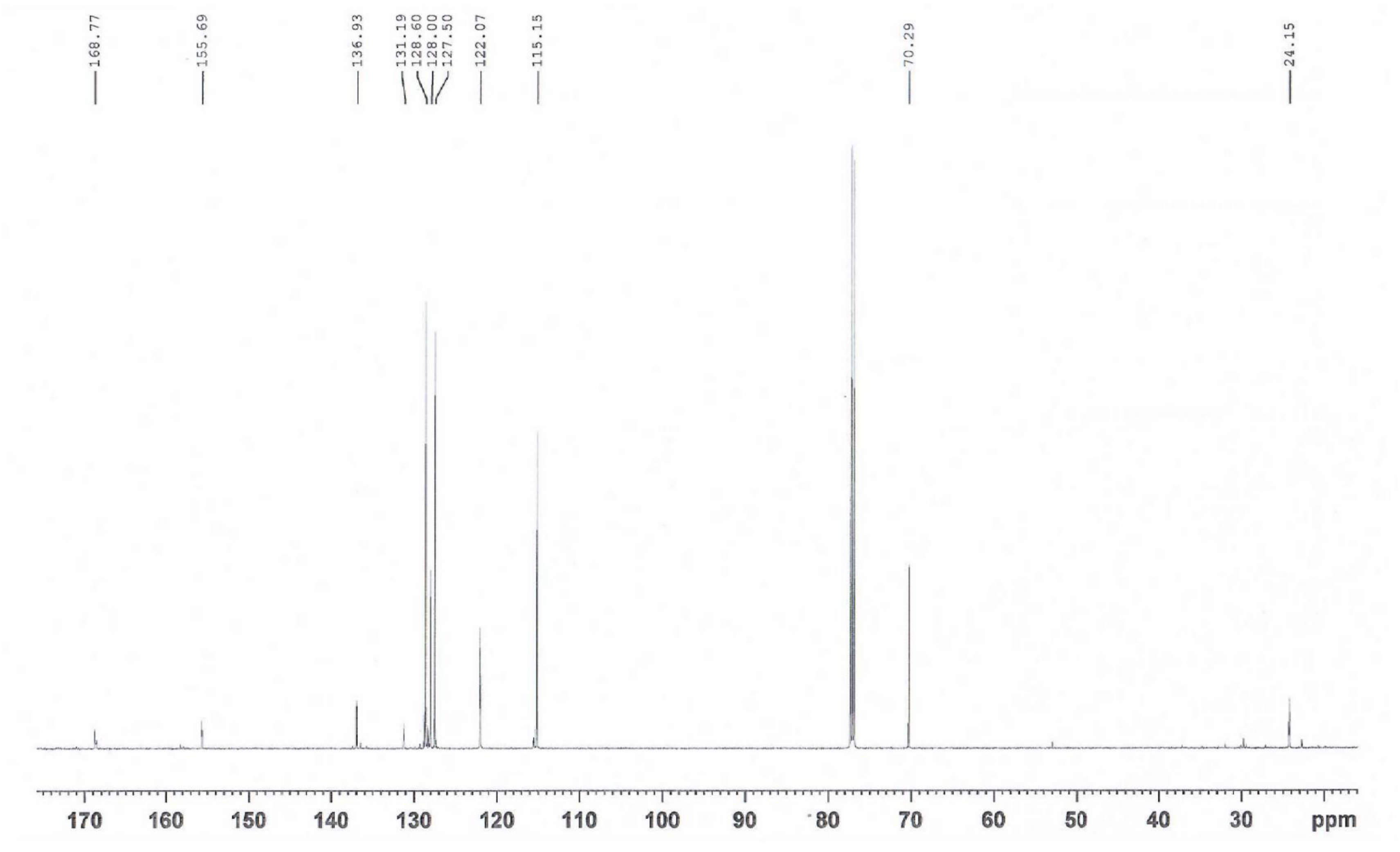


$^{13}C$ NMR spectrum of 4-(benzyloxy)acetanilide **2l** in $CDCl_3$

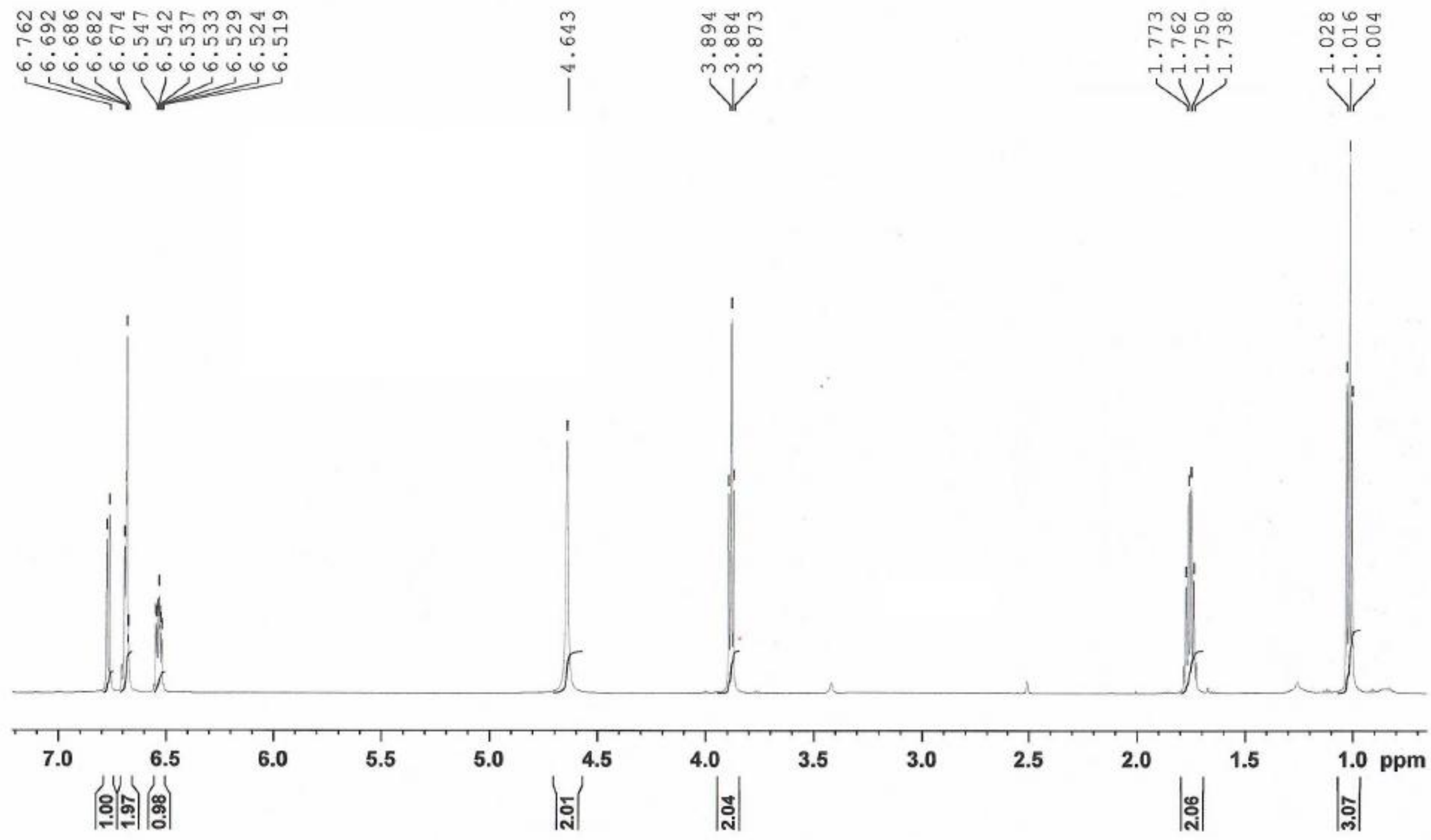


$^{1}$H NMR spectrum of 2-(propoxy)aniline **3a** in $DMSO_{-d6}$

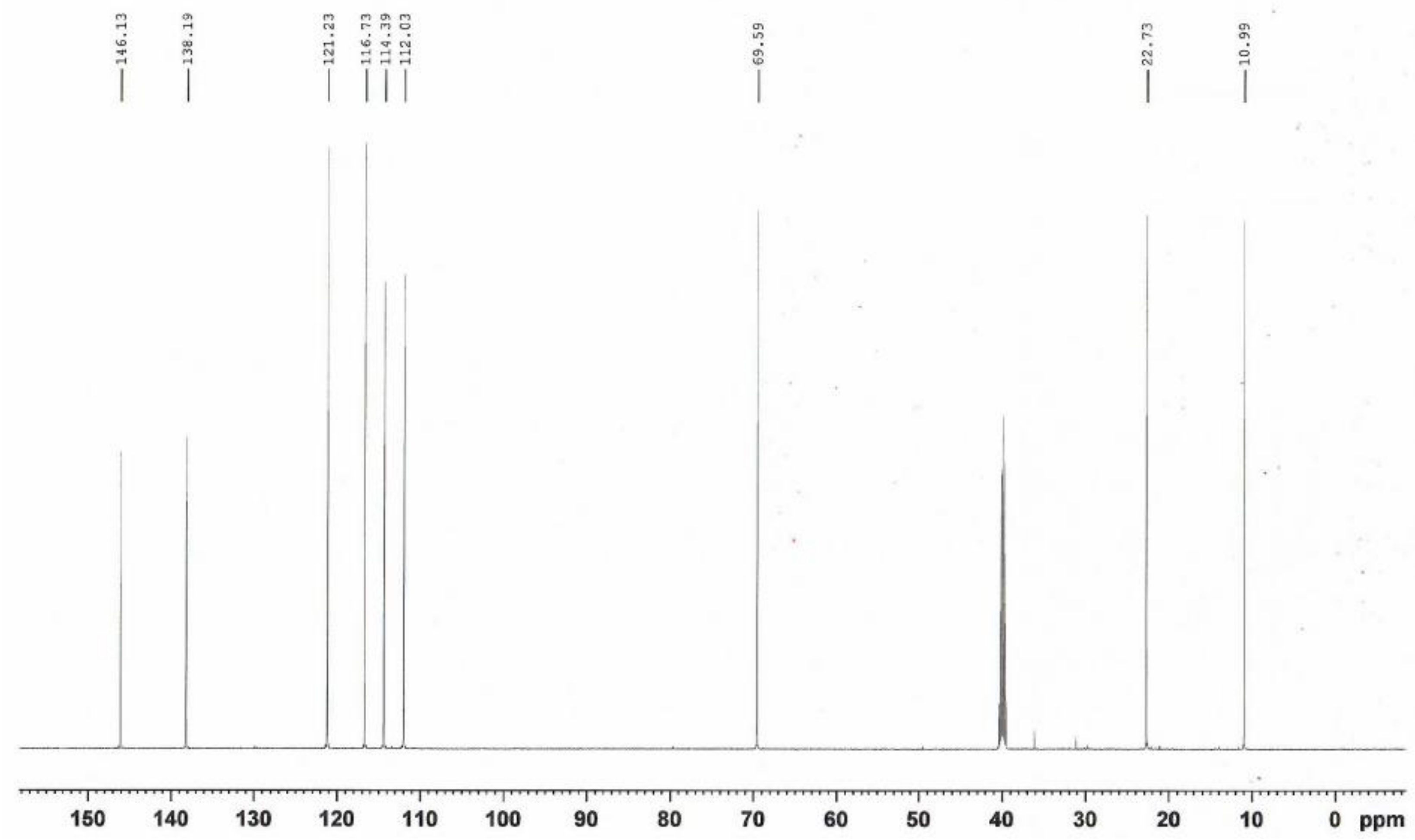


$^{13}C$ NMR spectrum of 2-(propoxy)aniline **3a** in $DMSO_{-d6}$

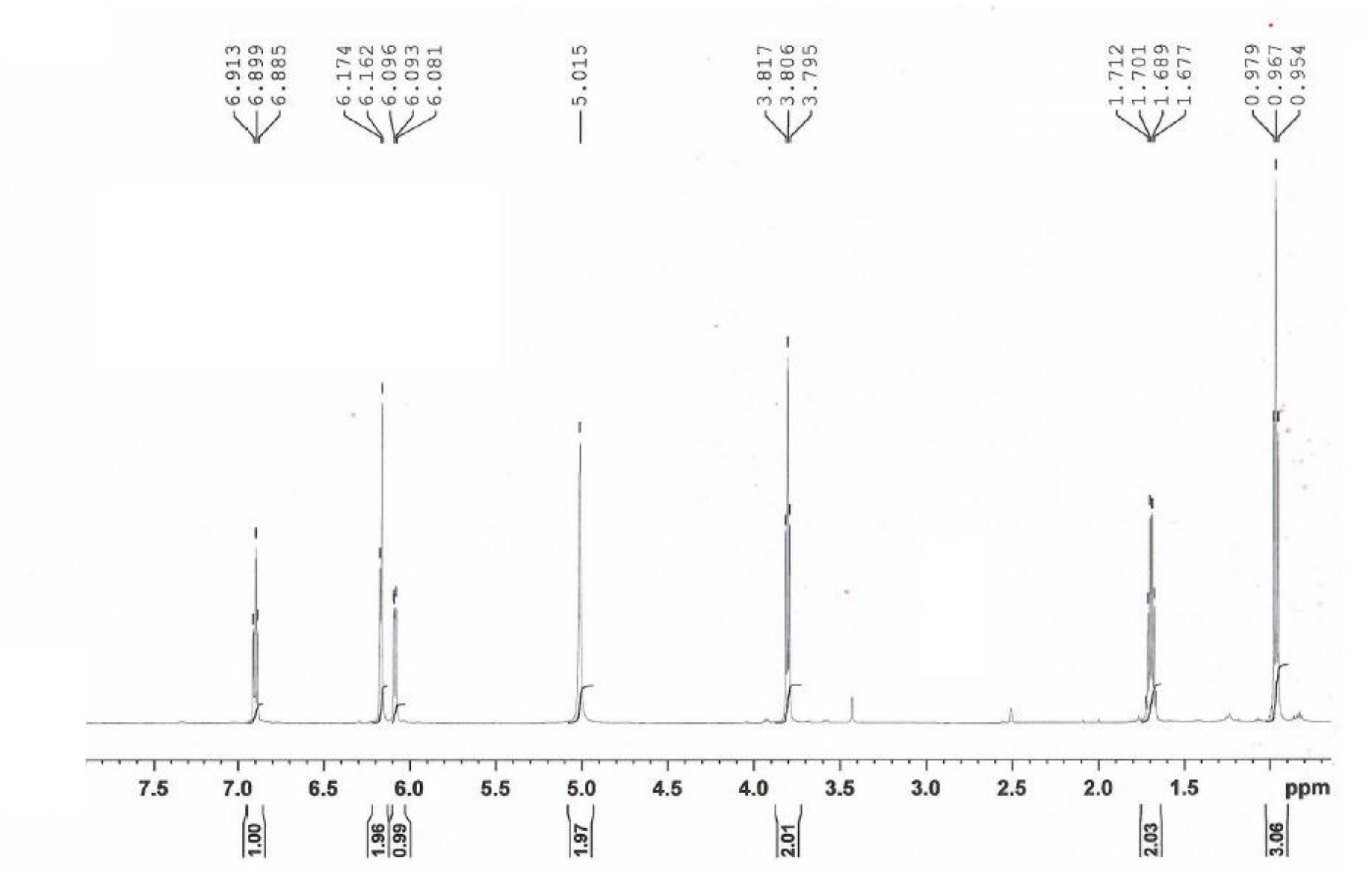


$^{1}$H NMR spectrum of 3-(propoxy)aniline **3b** in DMSO-d6

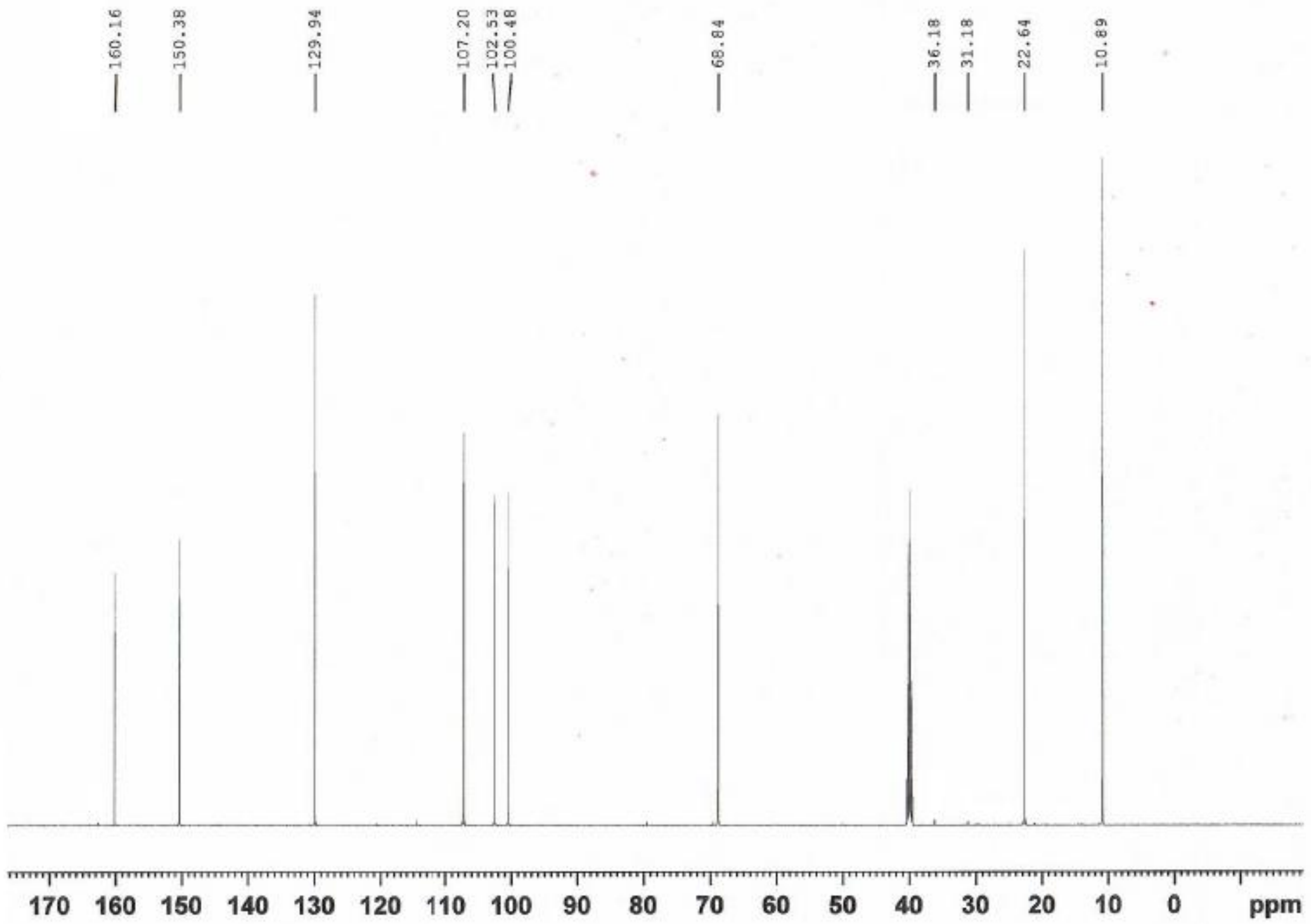


$^{13}C$ NMR spectrum of 3-(propoxy)aniline **3b** in DMSO$_{\text{-d6}}$

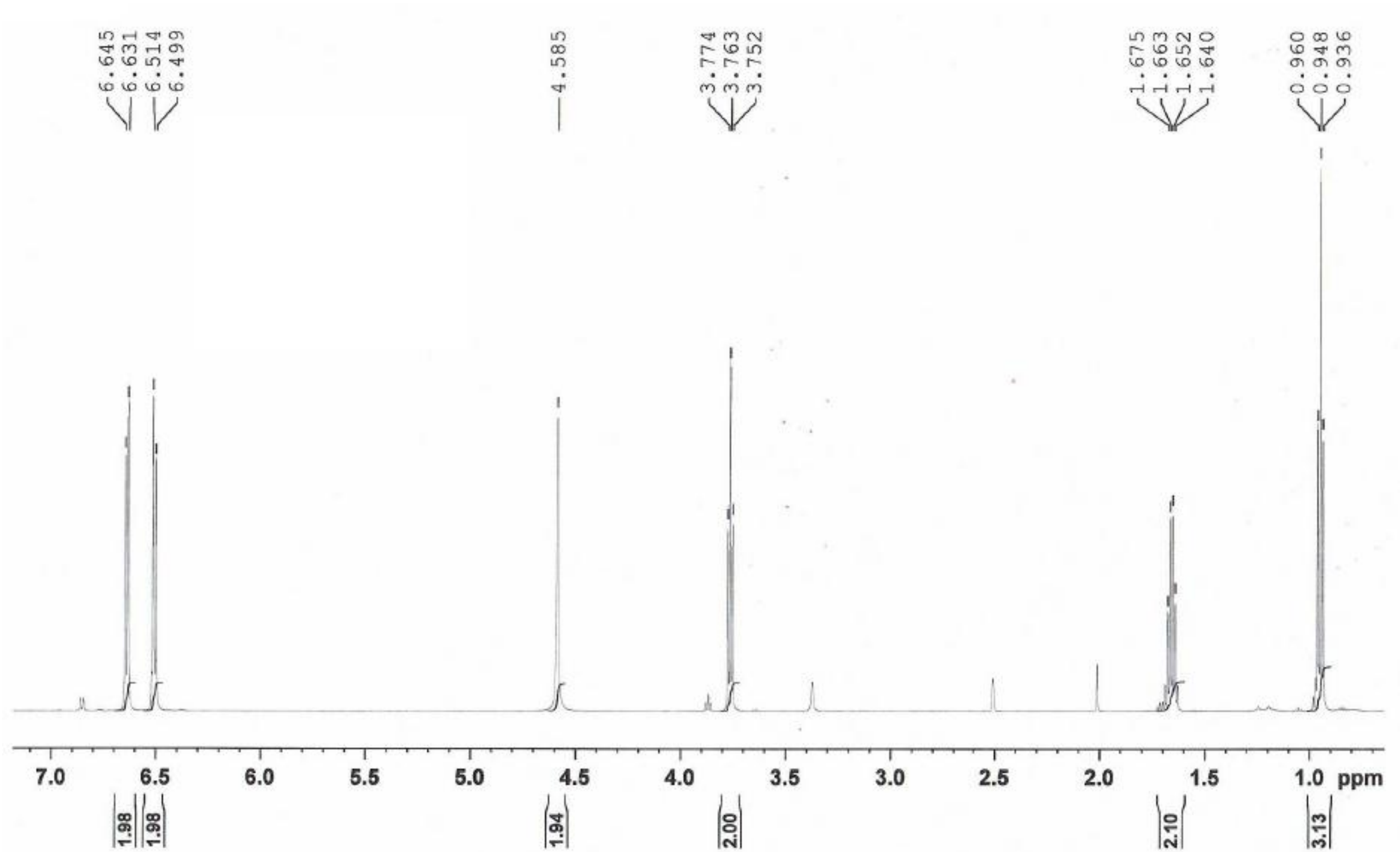


$^{1}$H NMR spectrum of 4-(propoxy)aniline **3c** in DMSO$_{-d6}$

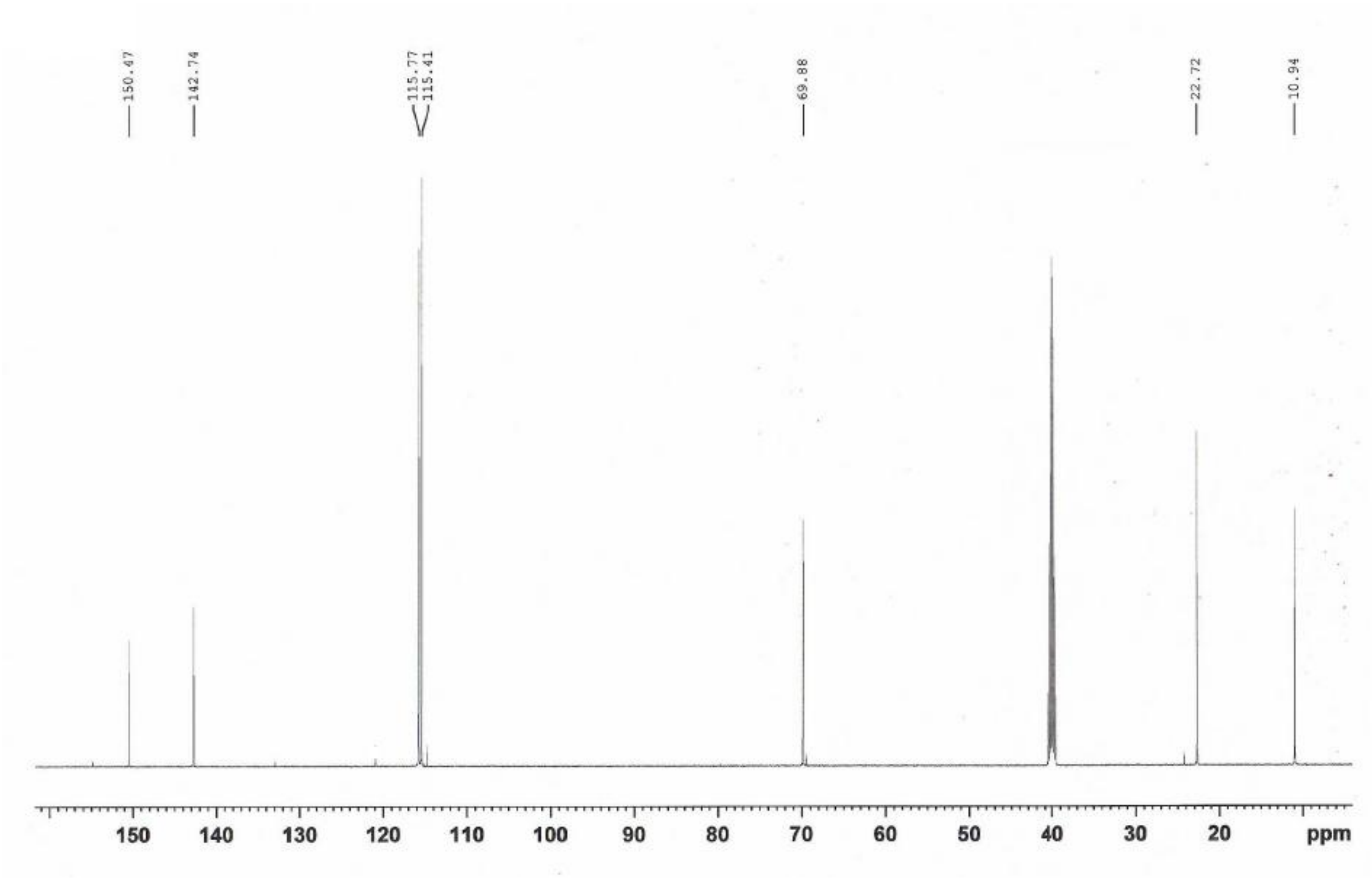


$^{13}C$ NMR spectrum of 4-(propoxy)aniline **3c** in DMSO$_{-d6}$

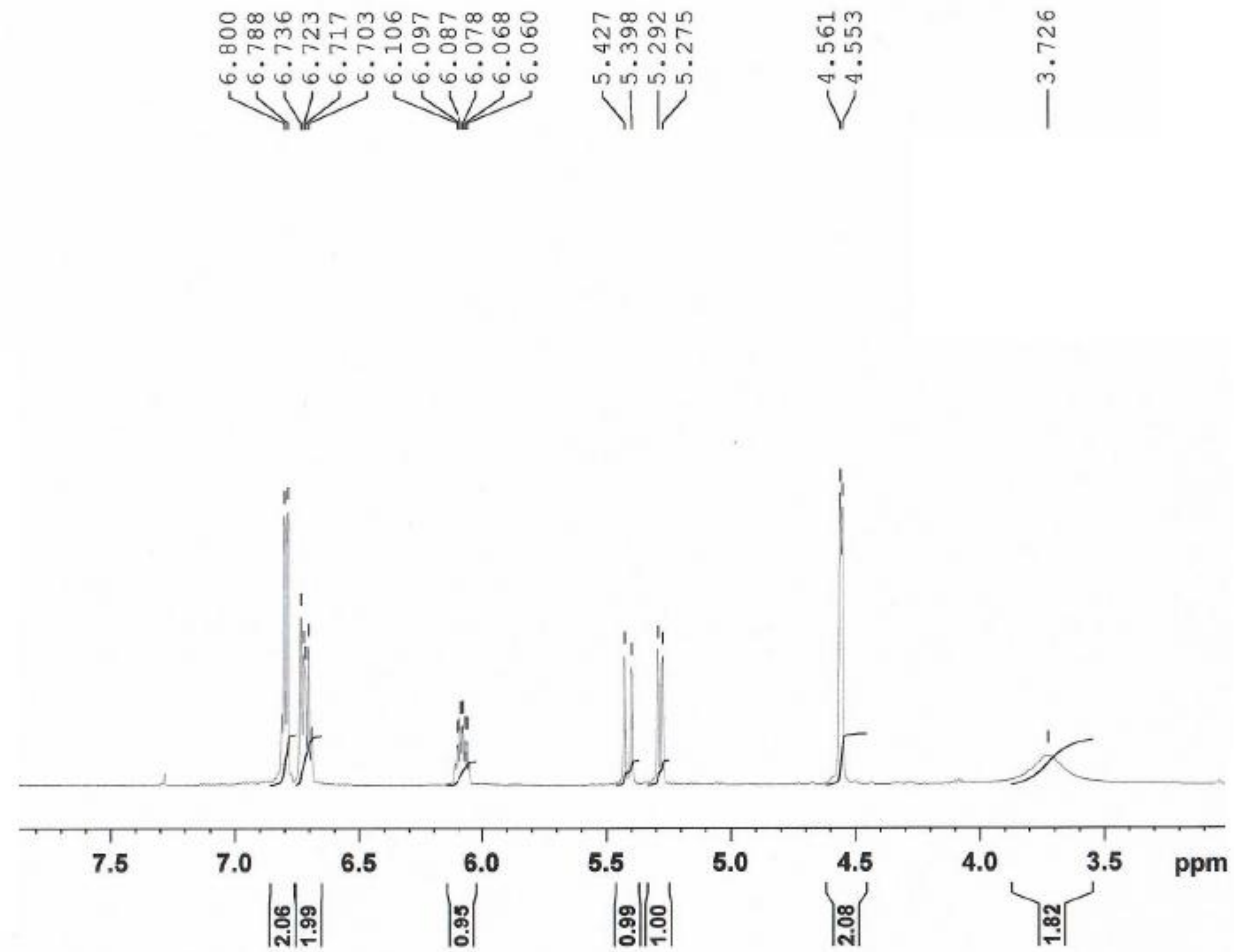


$^{1}$H NMR spectrum of 2-(allyloxy)aniline **3d** in $CDCl_3$

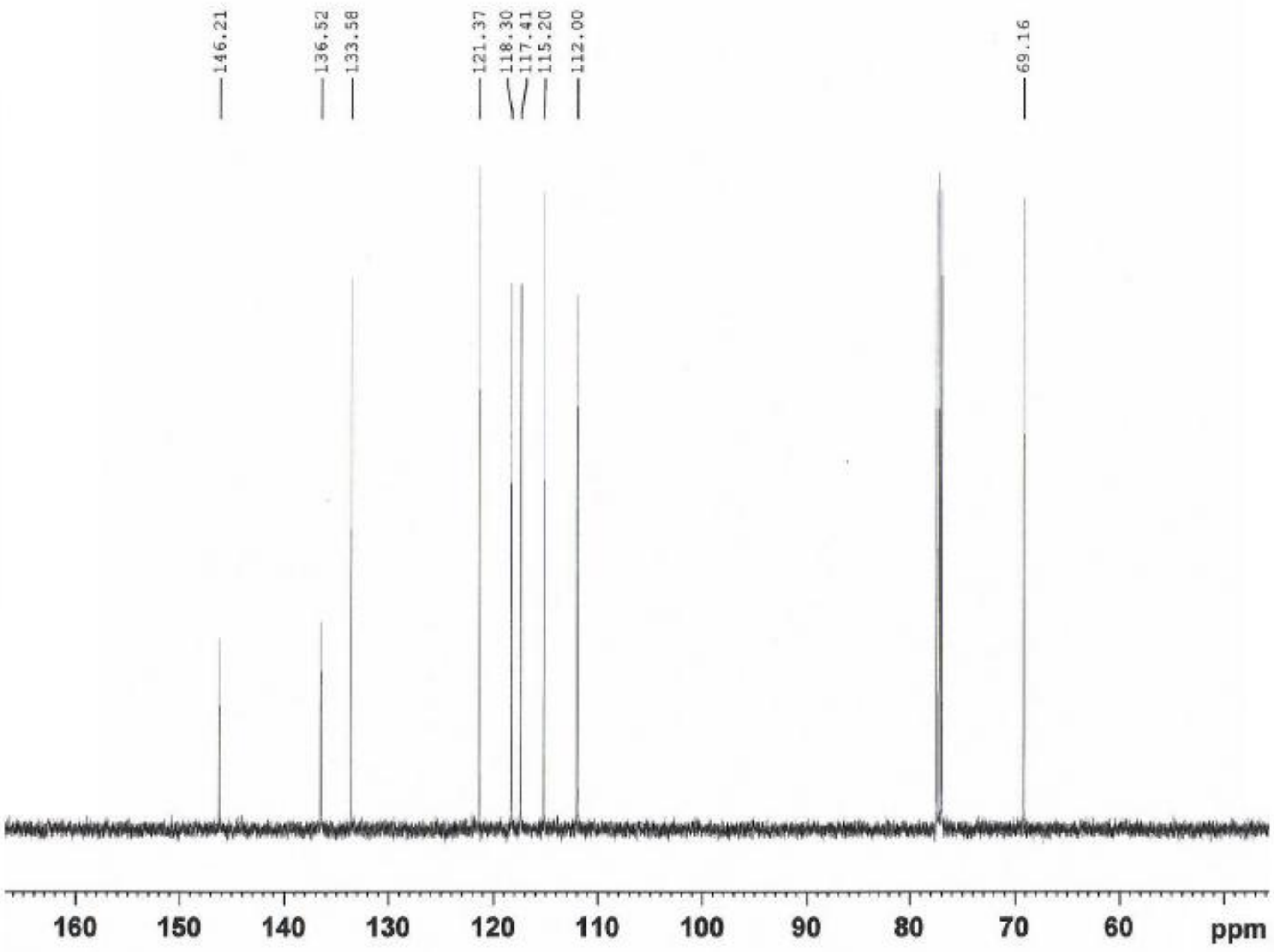


$^{13}$C NMR spectrum of 2-(allyloxy)aniline **3d** in $CDCl_3$

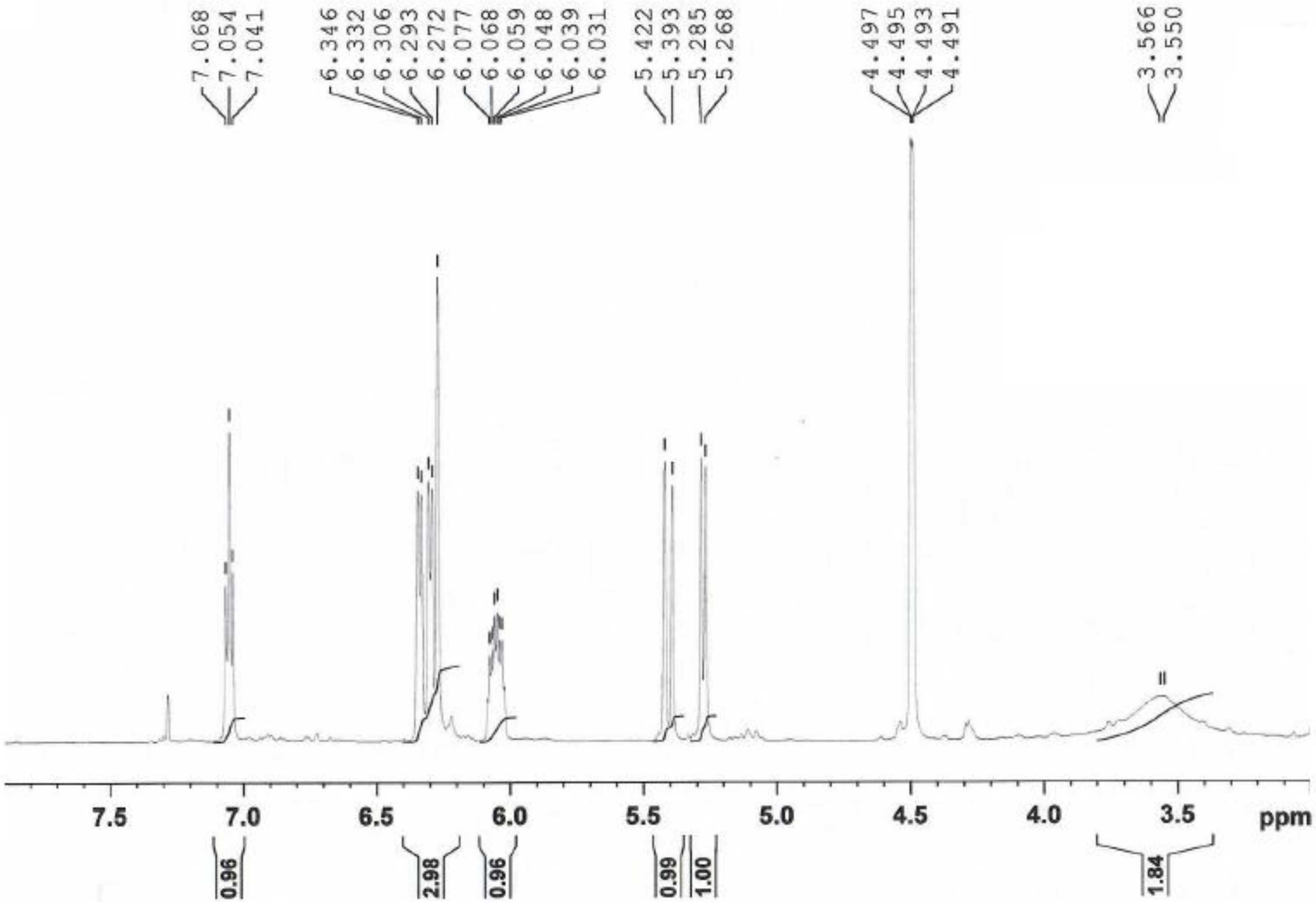


$^{1}$H NMR spectrum of 3-(allyloxy)aniline **3e** in $CDCl_3$

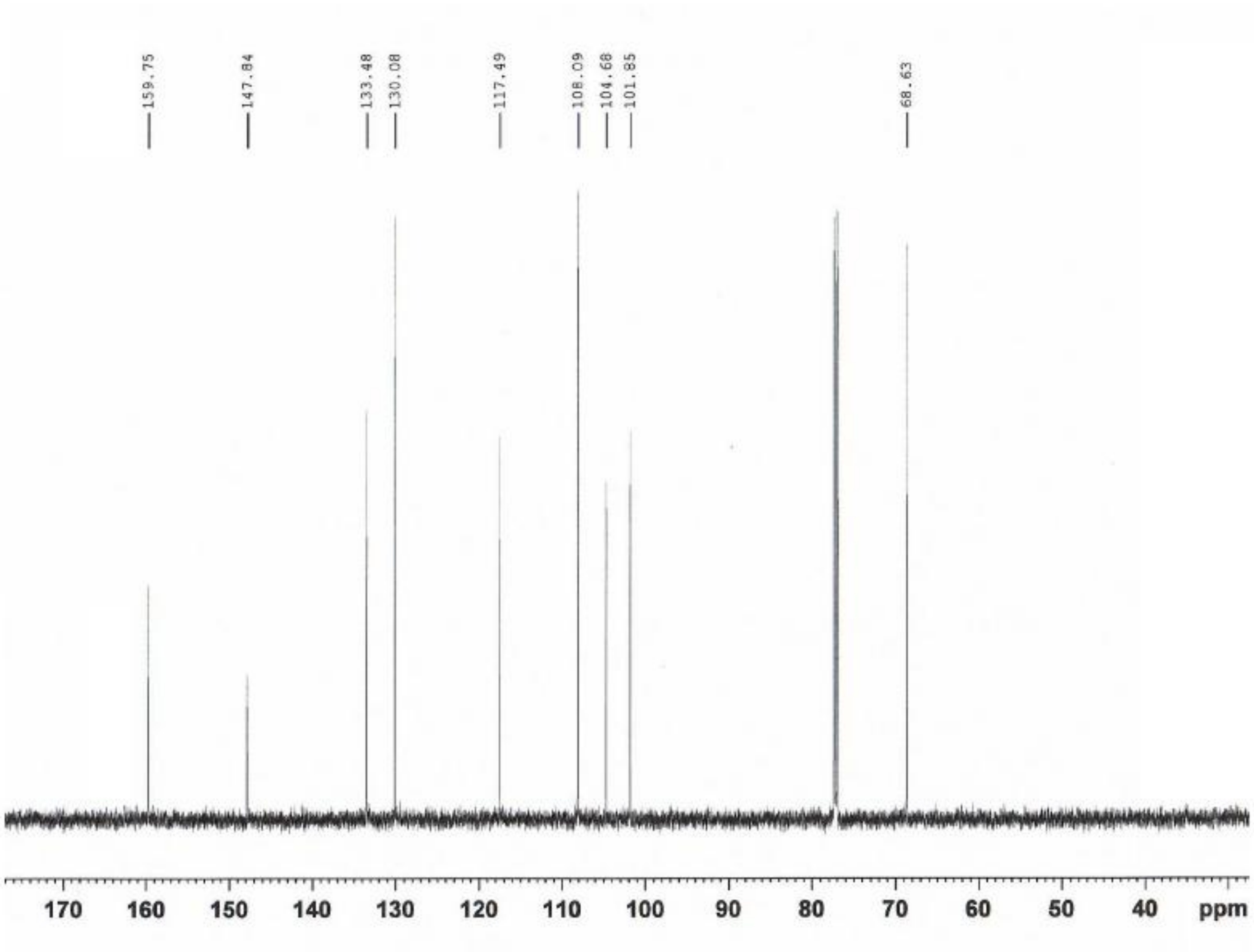


$^{13}$C NMR spectrum of 3-(allyloxy)aniline **3e** in $CDCl_3$

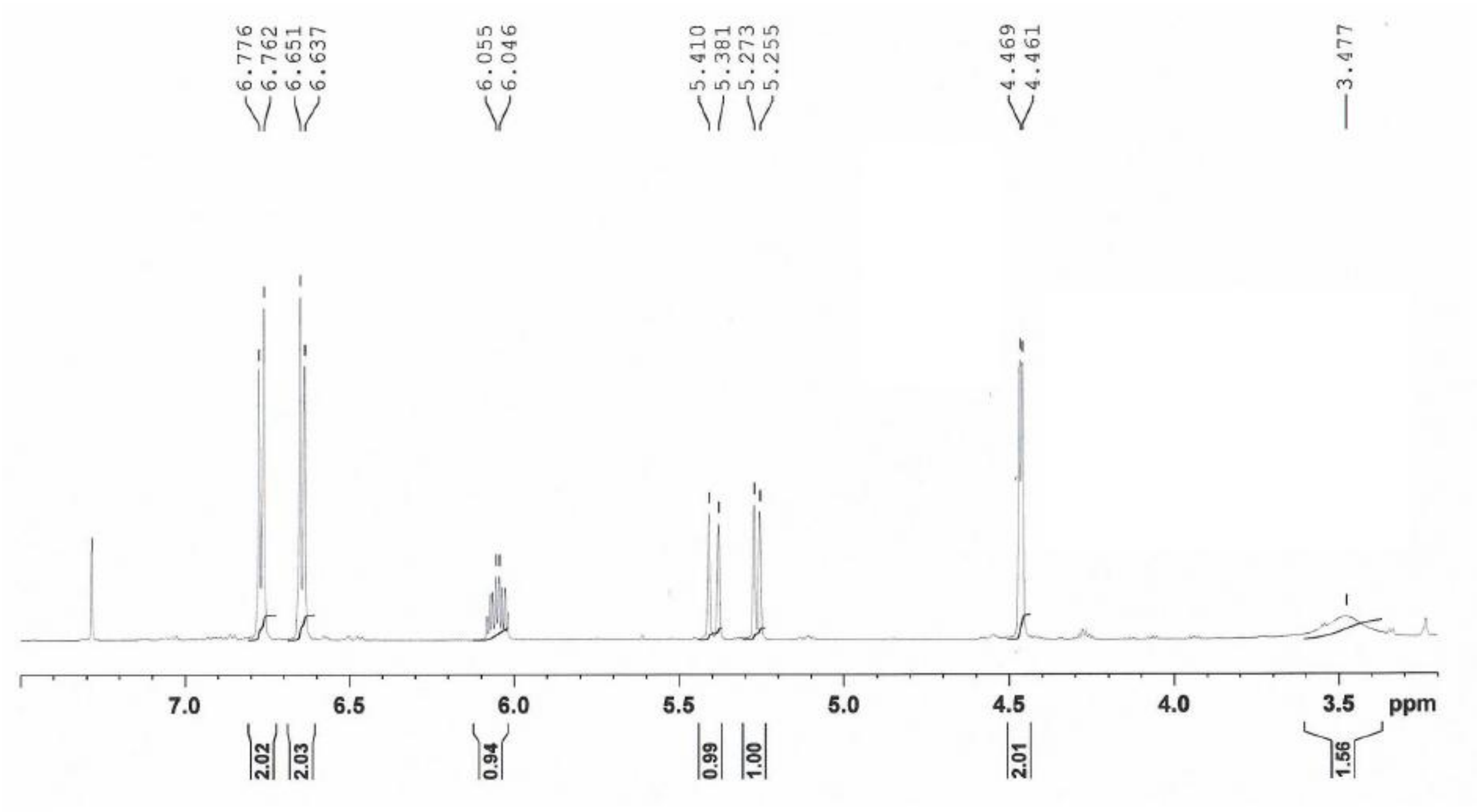


$^{1}$H NMR spectrum of 4-(allyloxy)aniline **3f** in $CDCl_3$

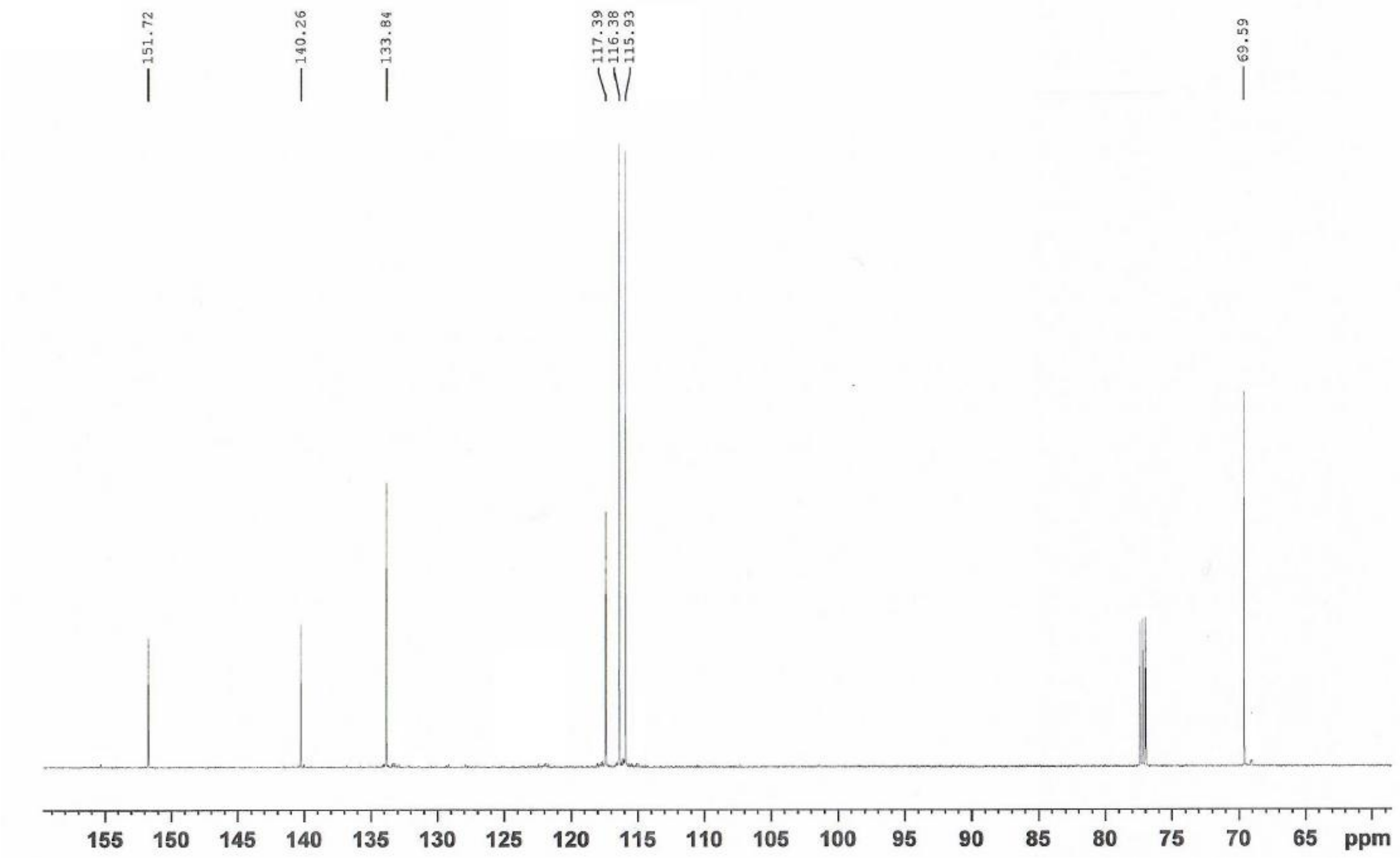


$^{13}$C NMR spectrum of 4-(allyloxy)aniline **3f** in $CDCl_3$

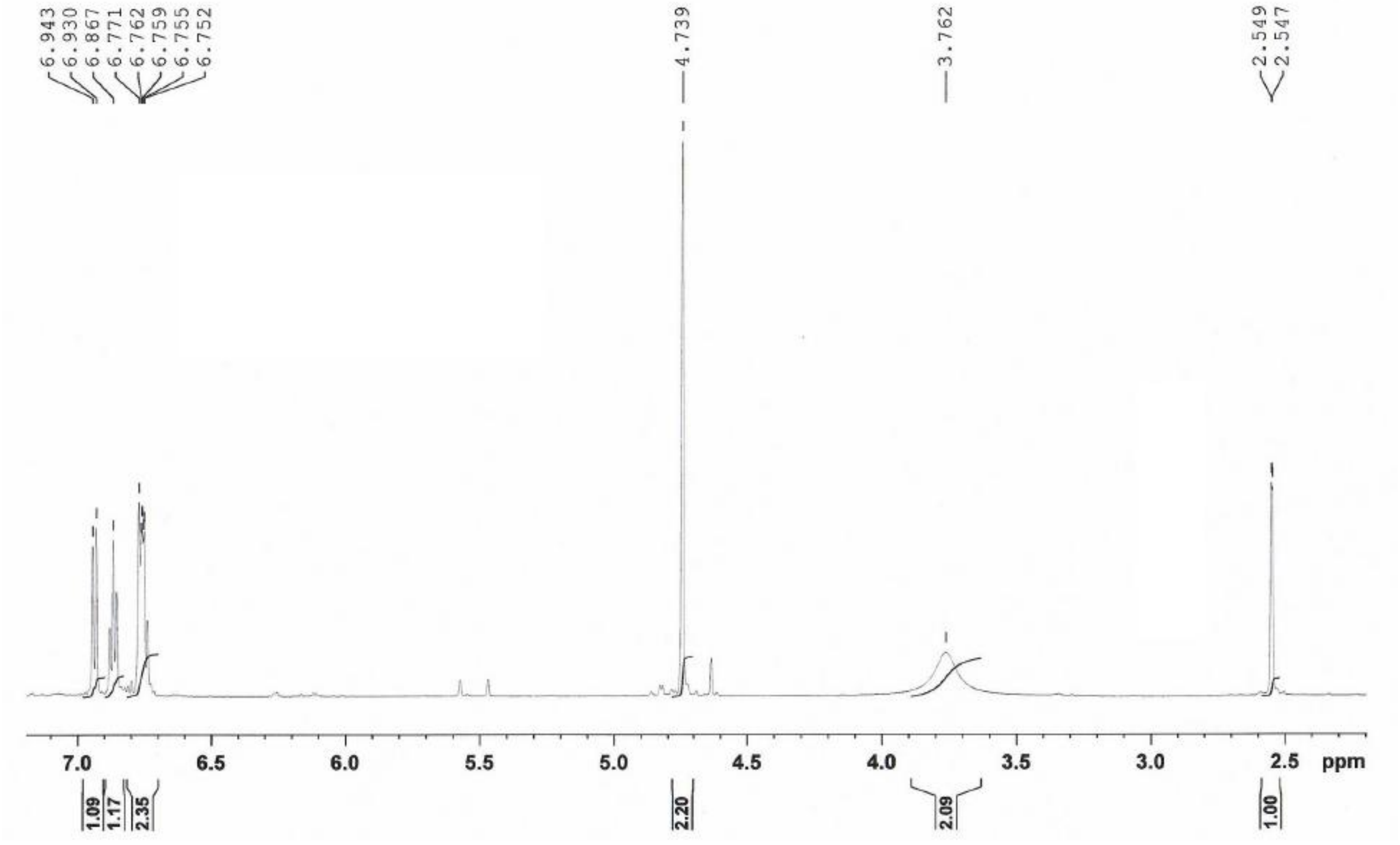


1H NMR spectrum of 2-(propargyloxy)aniline **3g** in $CDCl_3$

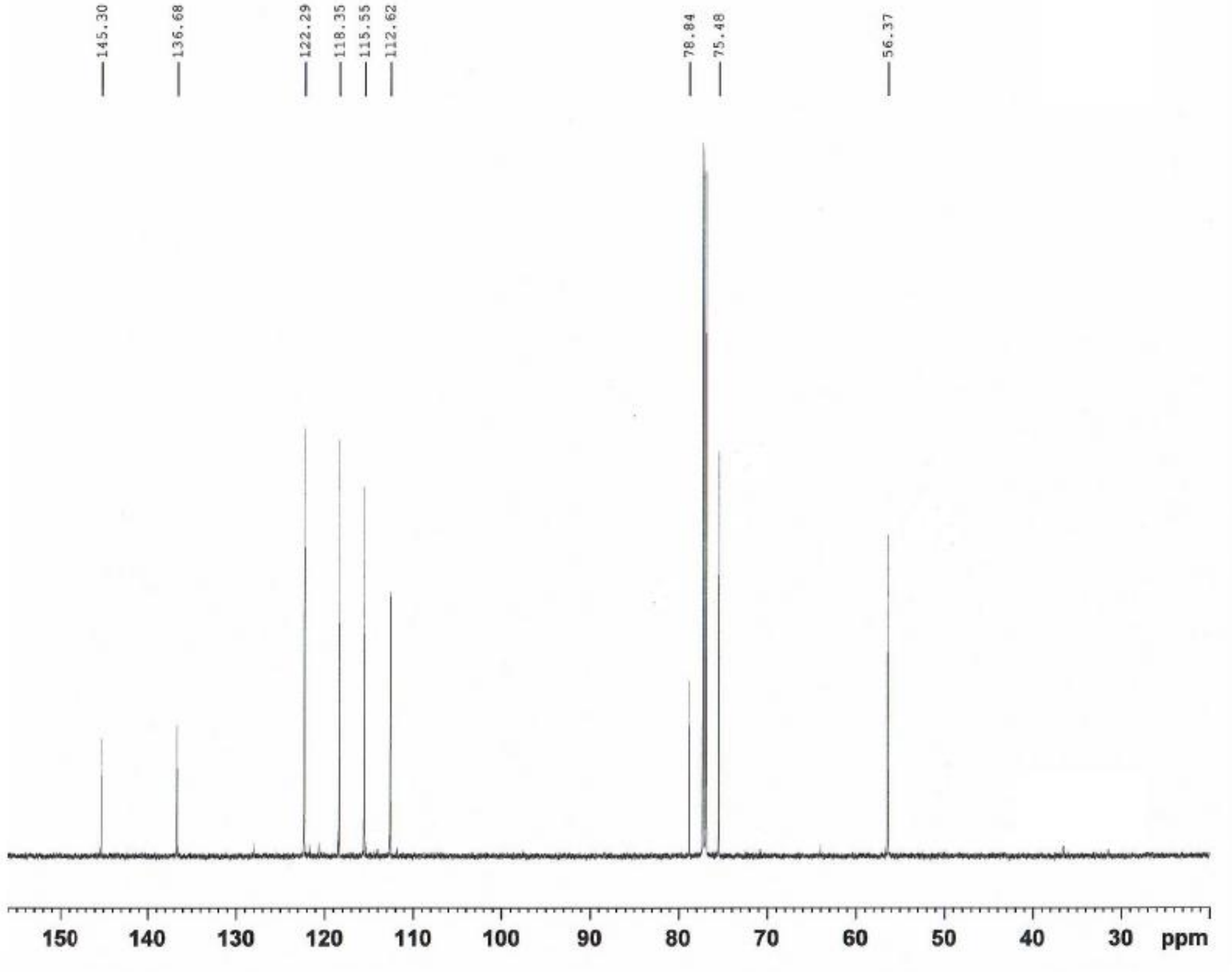


13C NMR spectrum of 2-(propargyloxy)aniline **3g** in $CDCl_3$

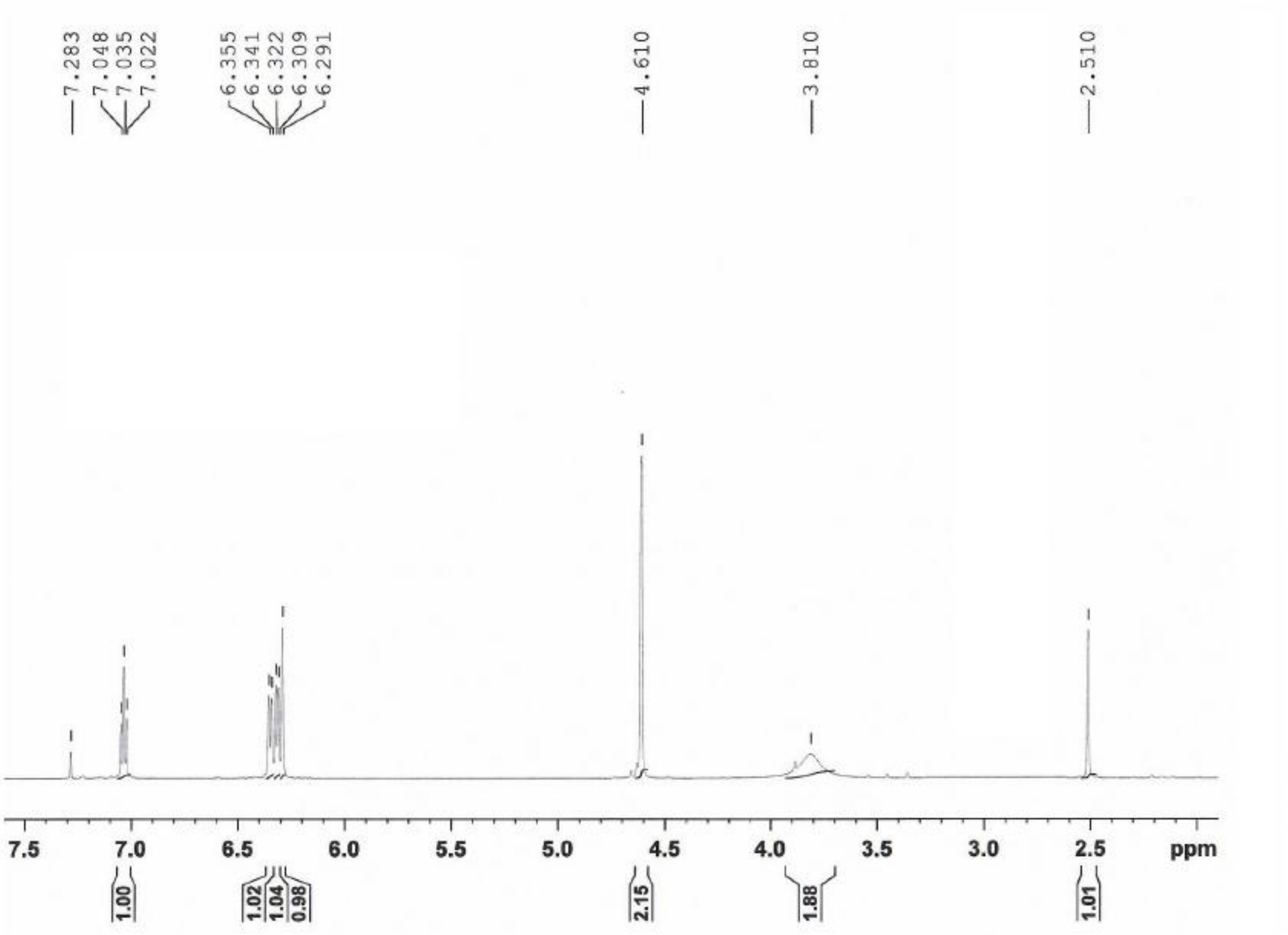


1H NMR spectrum of 3-(propargyloxy)aniline **3h** in $CDCl_3$

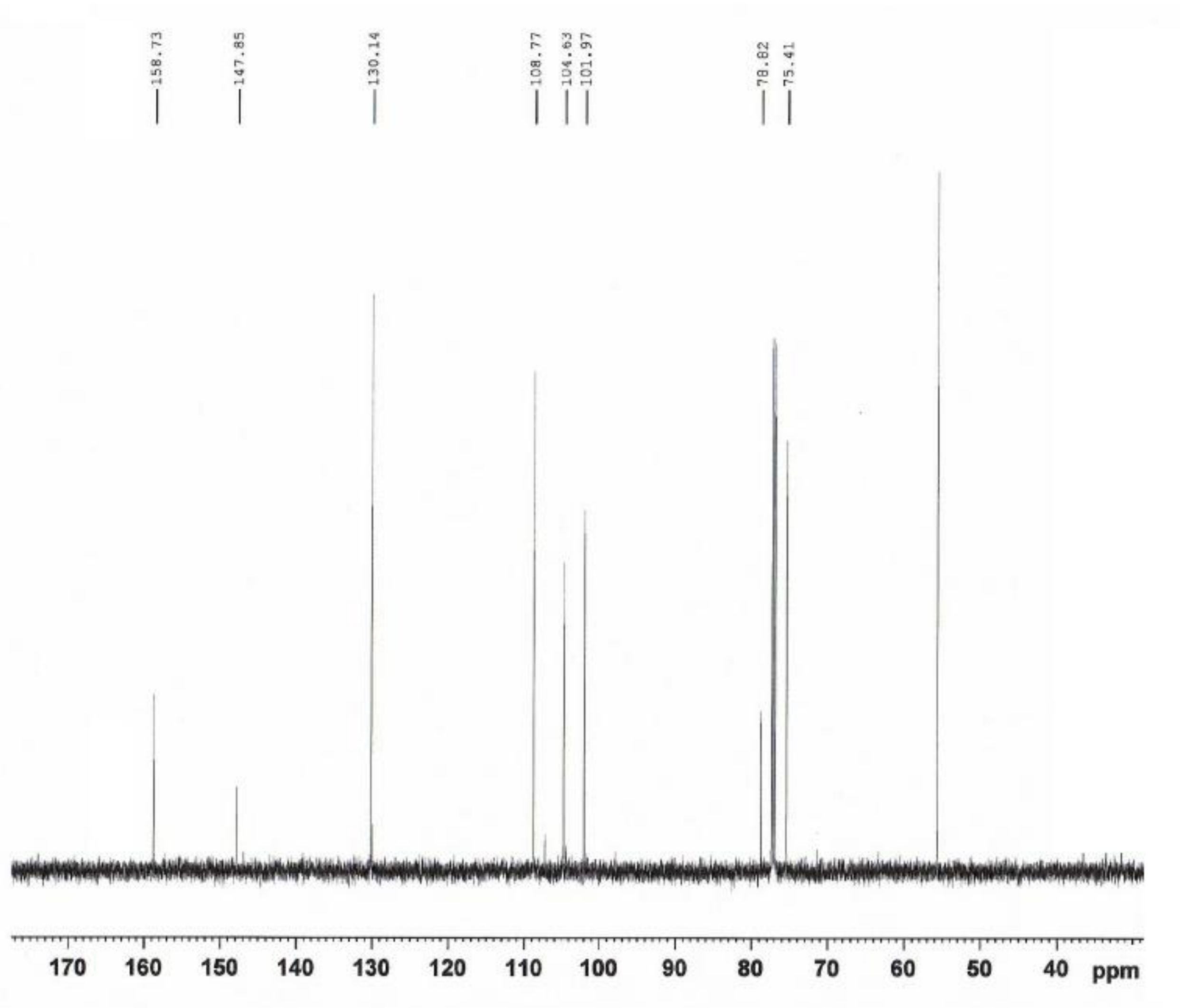


$^{13}C$ NMR spectrum of 3-(propargyloxy)aniline **3h** in $CDCl_3$

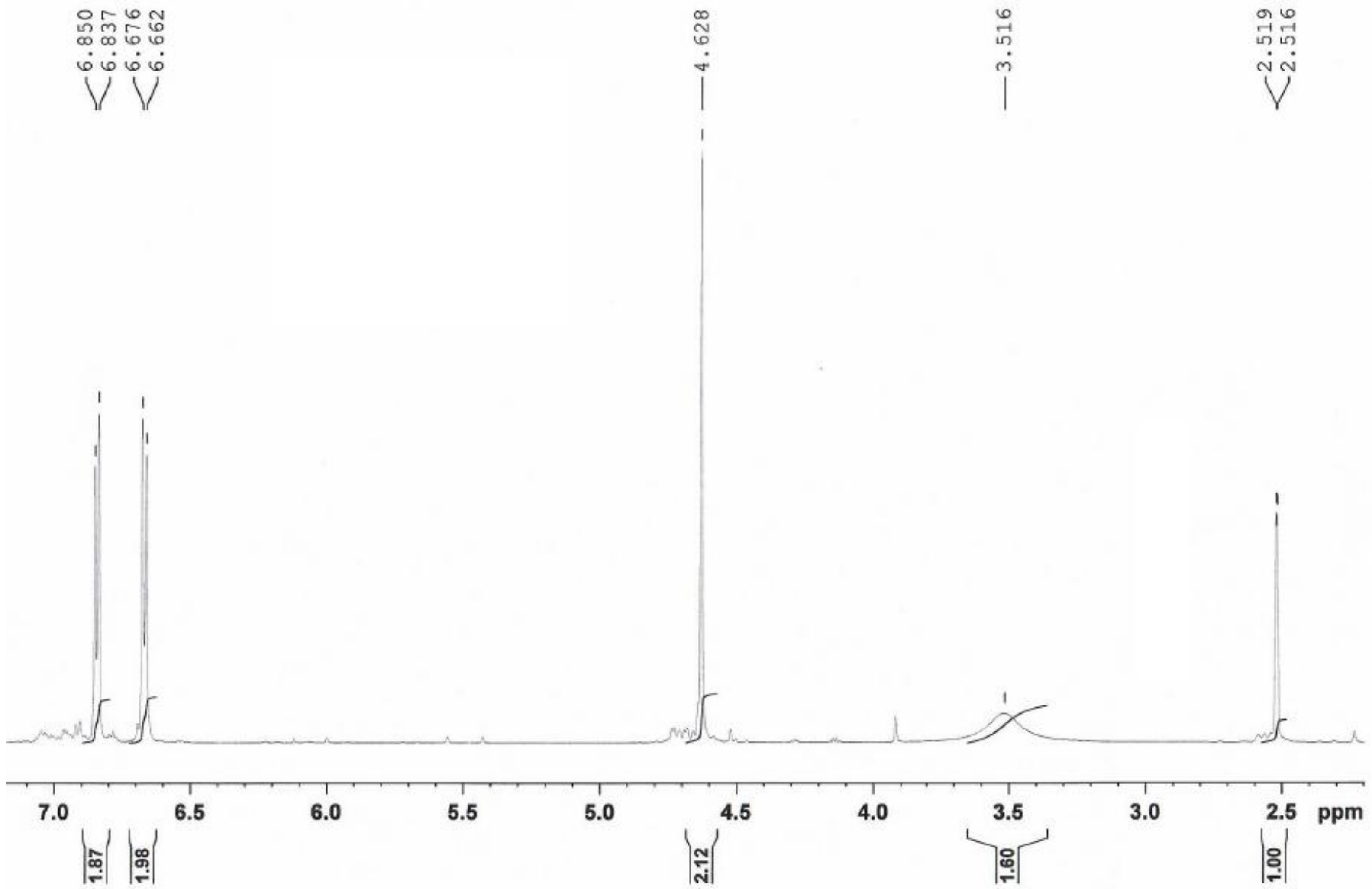


$^{1}$H NMR spectrum of 4-(propargyloxy)aniline **3i** in $CDCl_3$

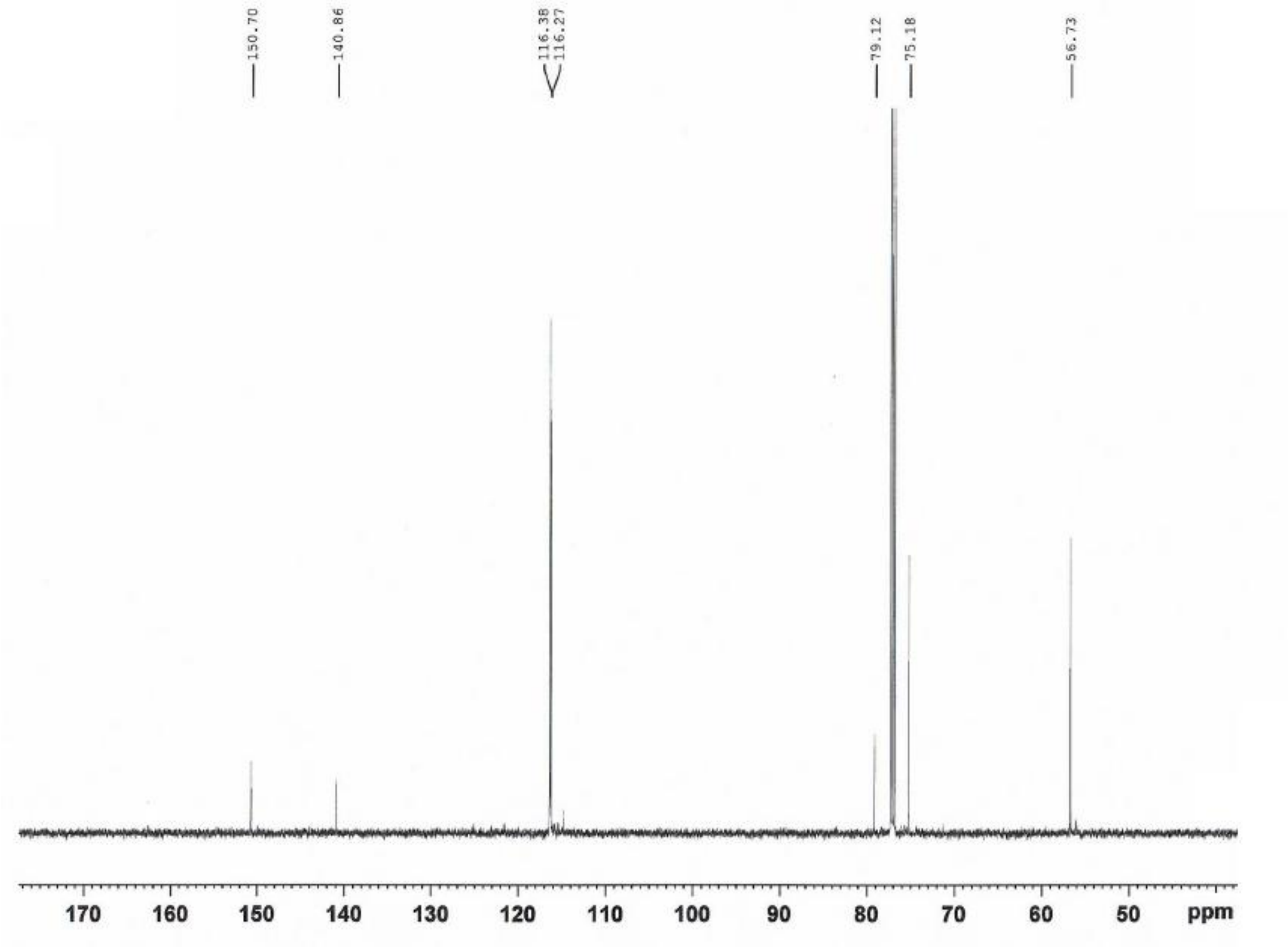


$^{13}C$ NMR spectrum of 4-(propargyloxy)aniline **3i** in $CDCl_3$

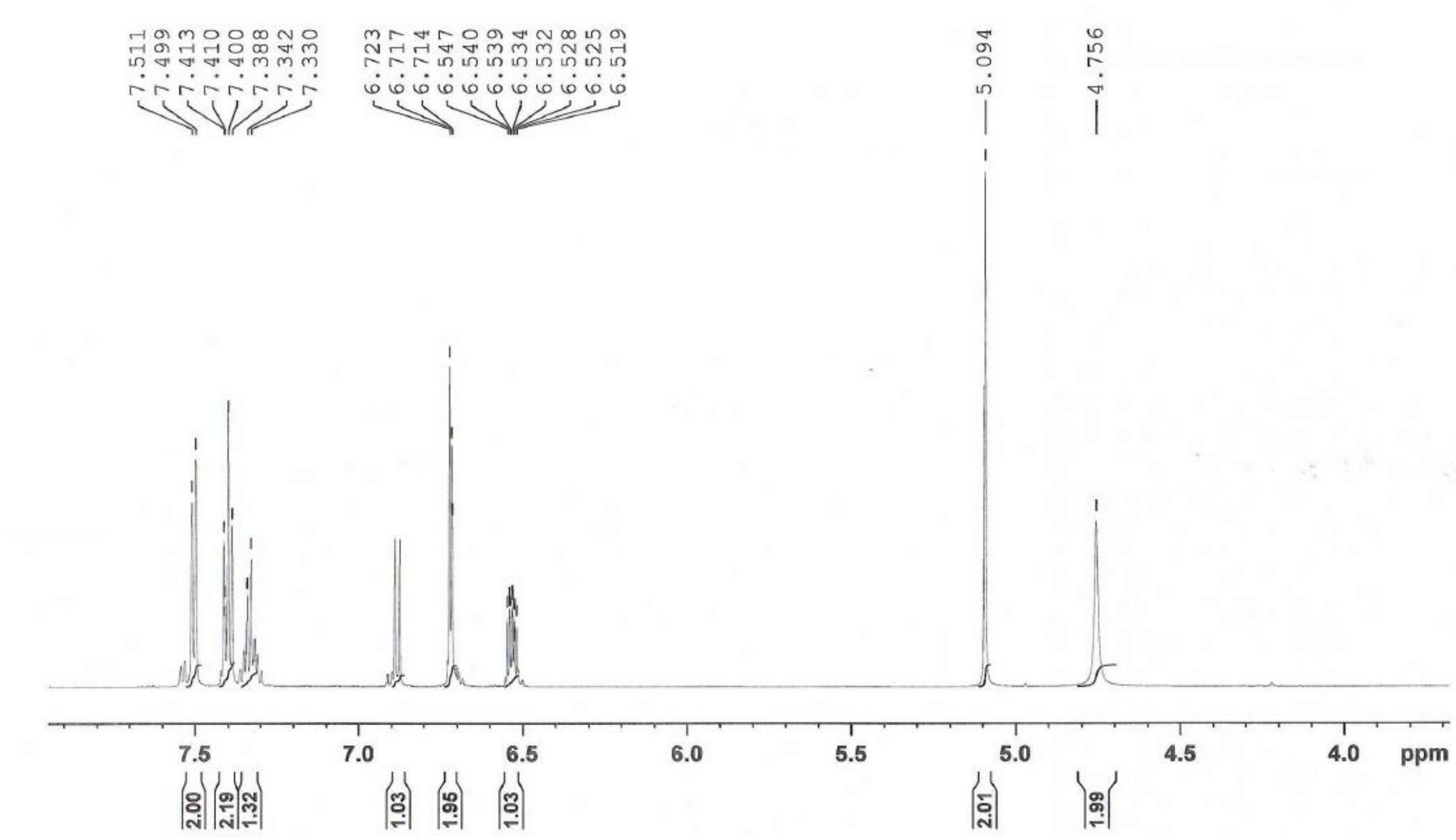


$^{1}$H NMR spectrum of 2-(benzyloxy)aniline **3j** in DMSO

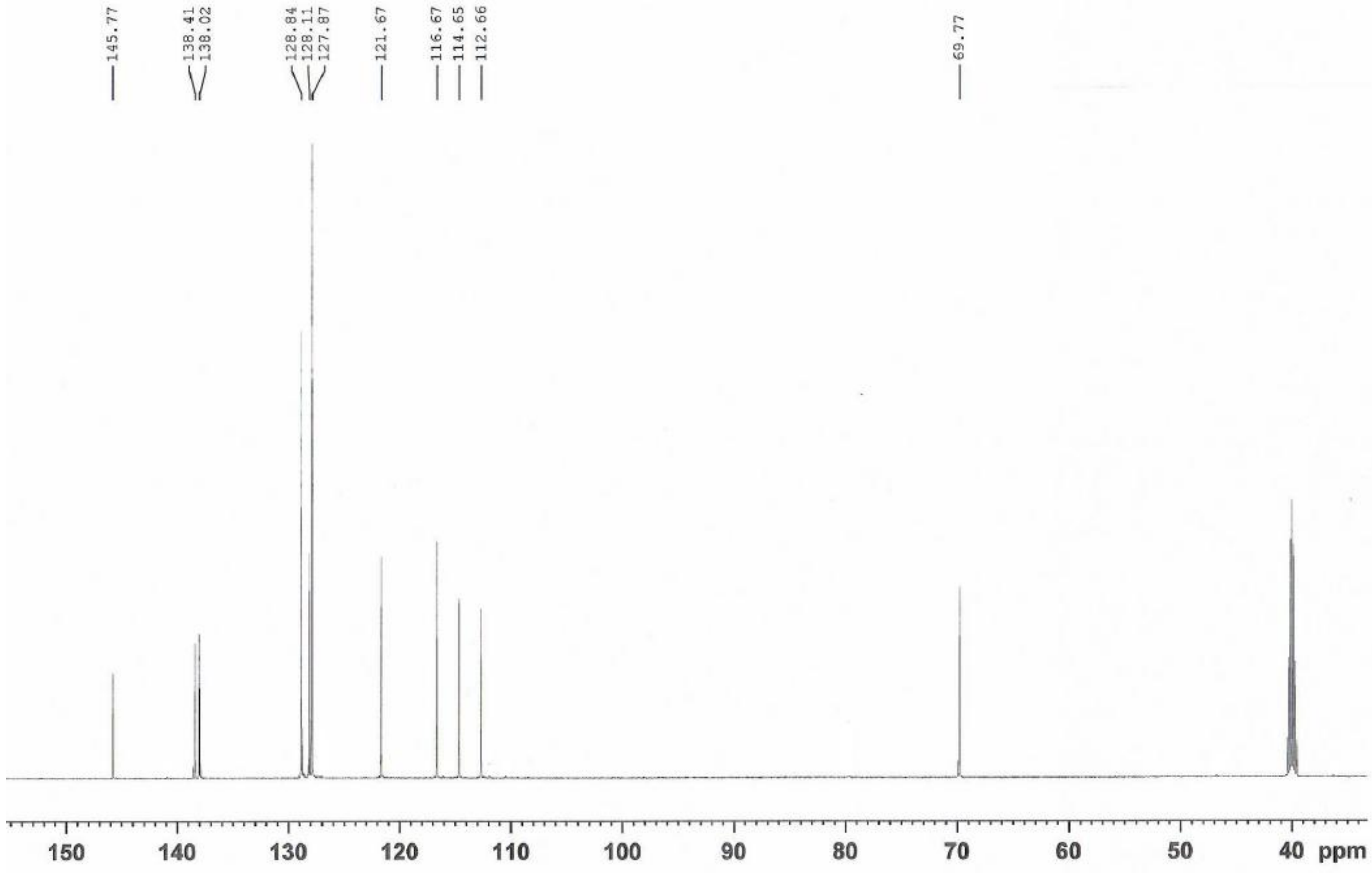


$^{13}$C NMR spectrum of 2-(benzyloxy)aniline **3j** in DMSO

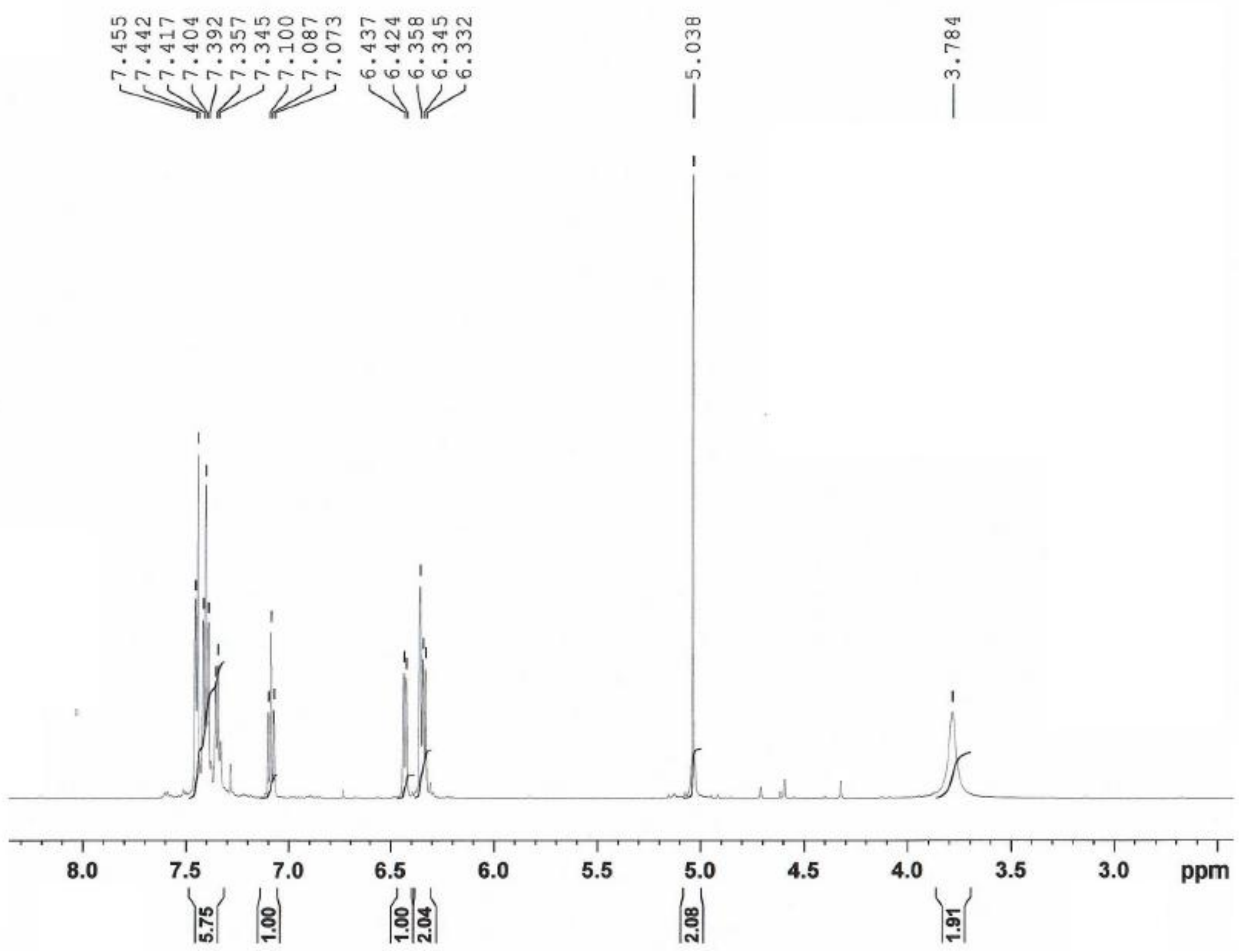


$^{1}$H NMR spectrum of 3-(benzyloxy)aniline **3k** in $CDCl_3$

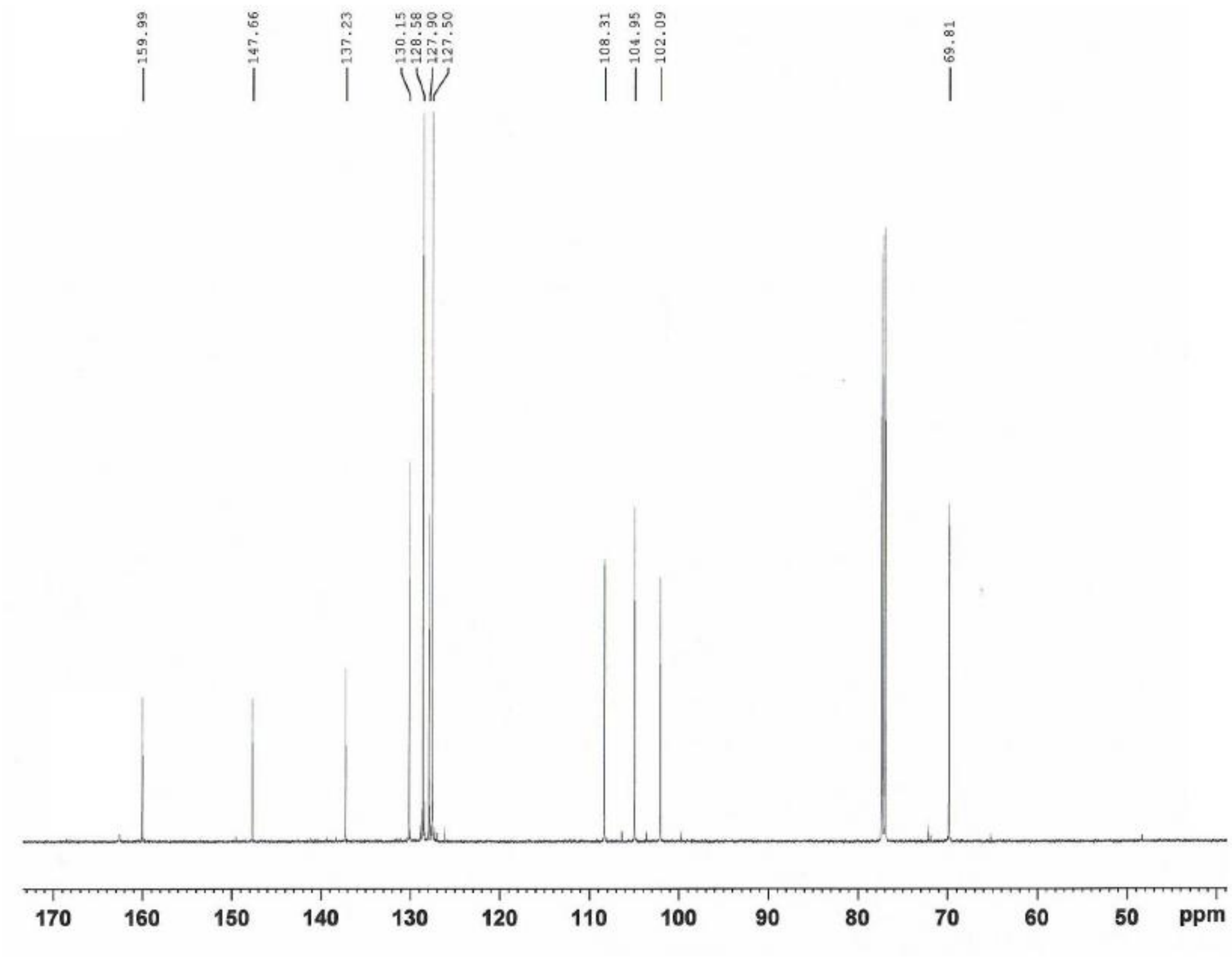


$^{13}$C NMR spectrum of 3-(benzyloxy)aniline **3k** in $CDCl_3$

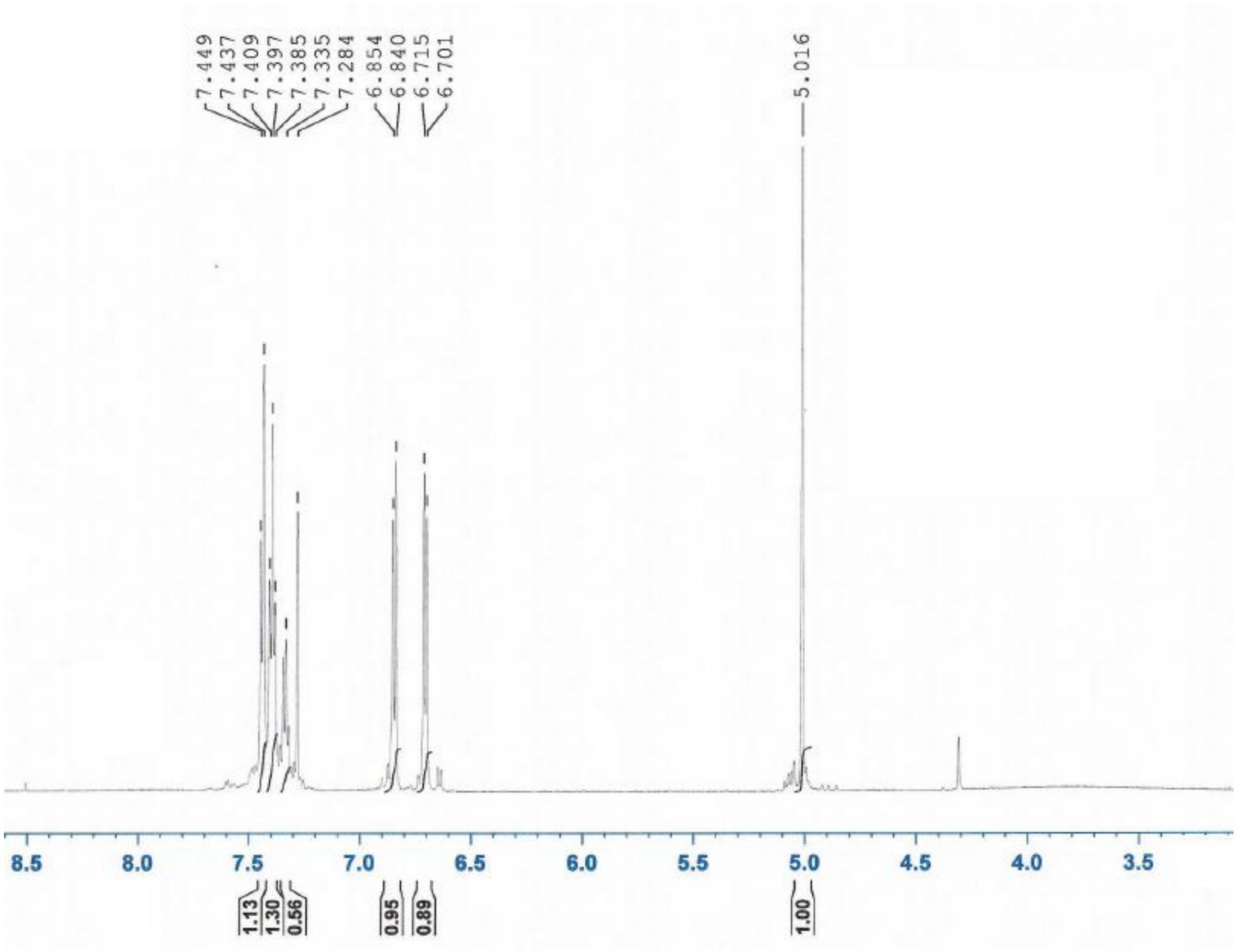


$^{1}$H NMR spectrum of 4-(benzyloxy)aniline **3l** in $CDCl_3$

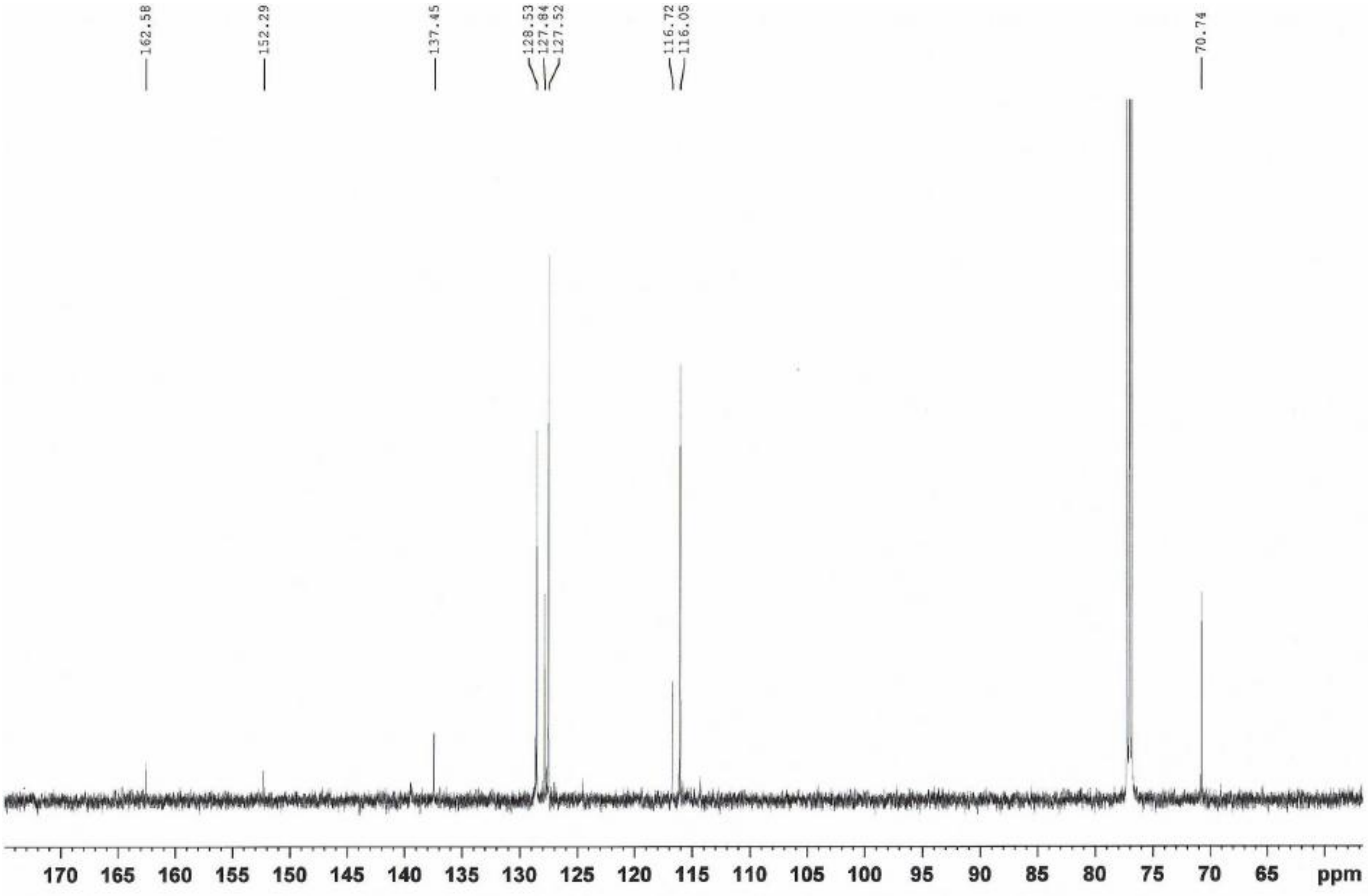


$^{13}C$ NMR spectrum of 4-(benzyloxy)aniline **3l** in $CDCl_3$

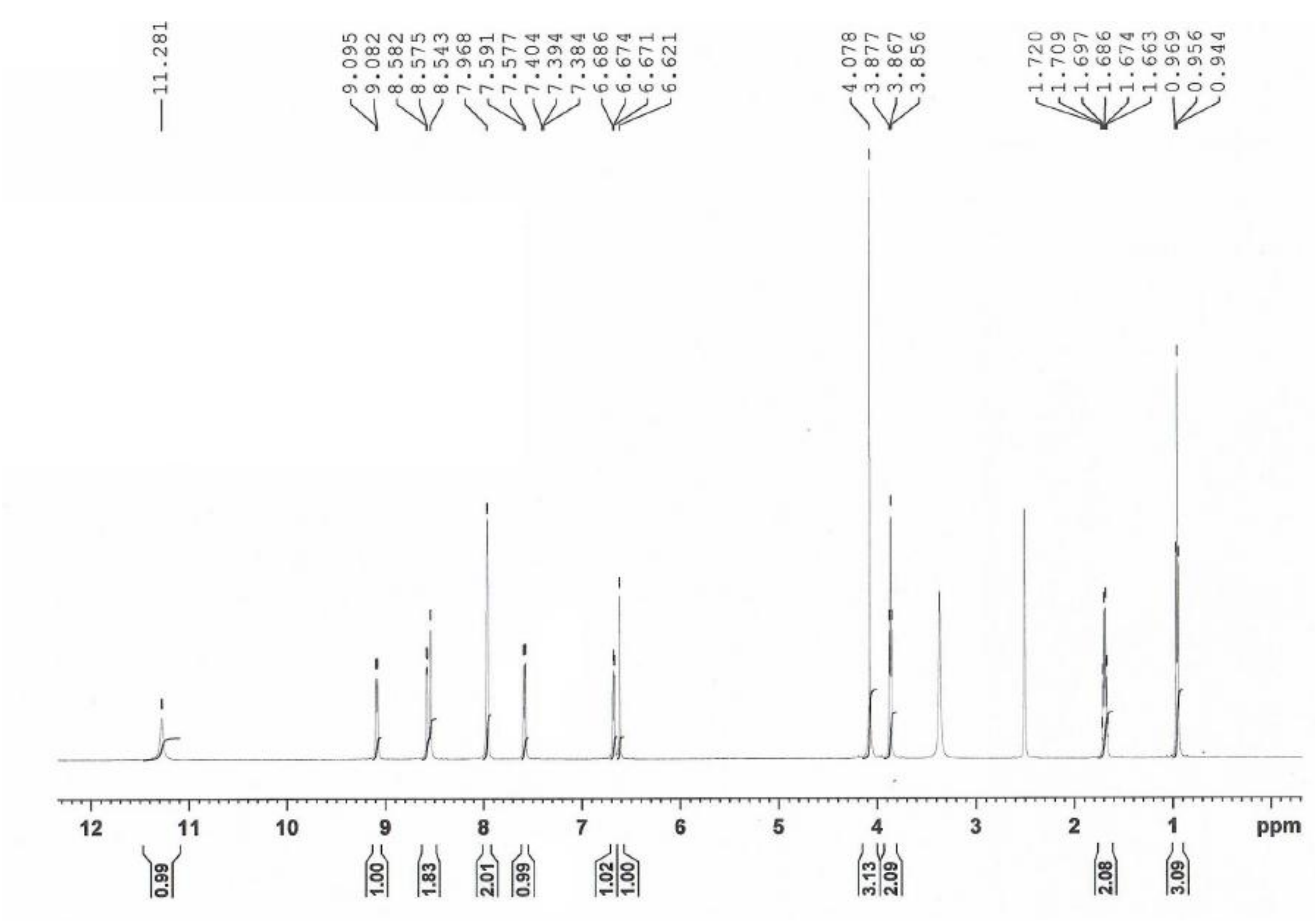


$^1$H NMR spectrum of 9-propoxy-5-methyl-12*H*-quino[3,4-*b*][1,4]benzothiazinium chloride **6a** in DMSO$_{-d6}$

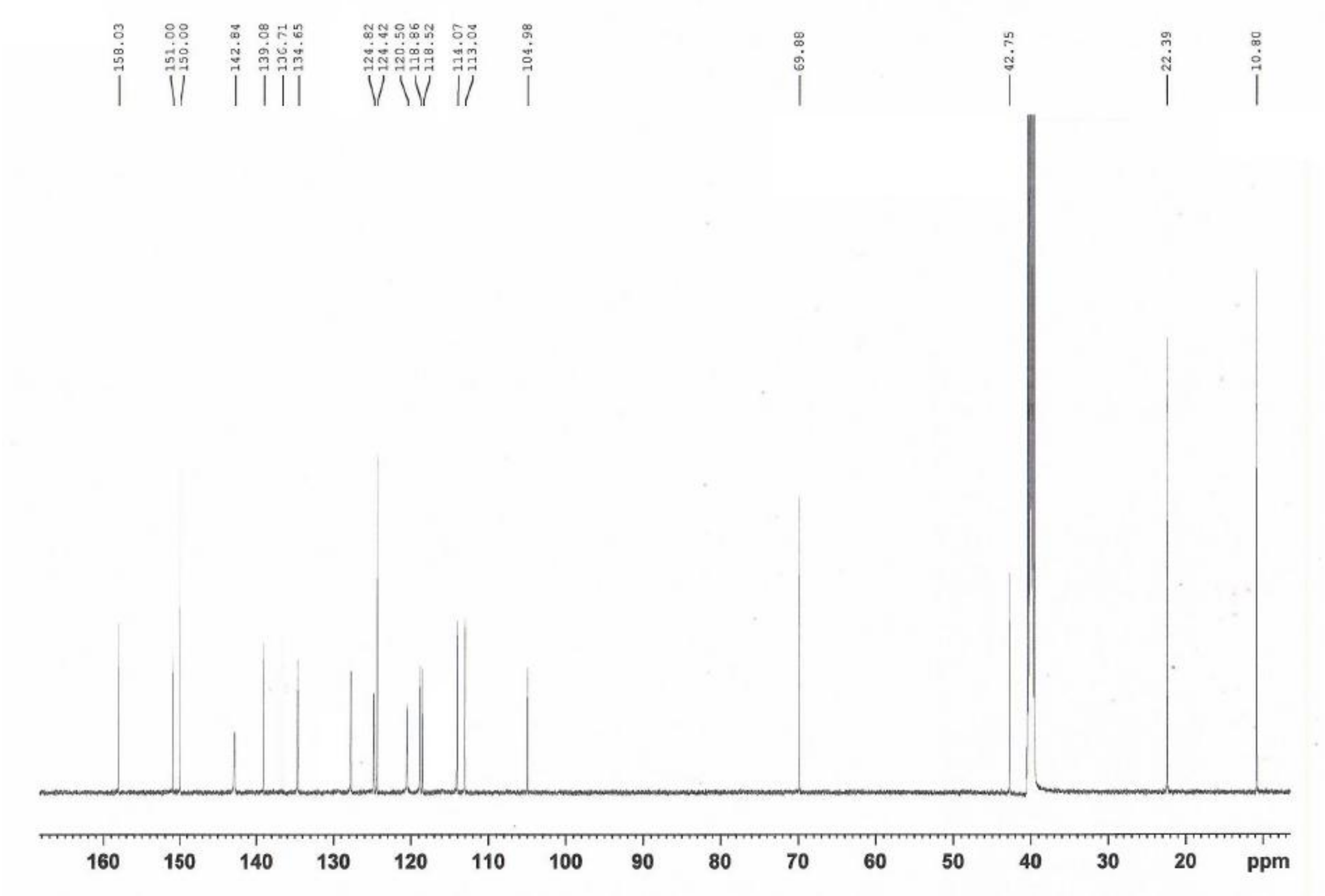


$^{13}$C NMR spectrum of 9-propyloxy-5-methyl-12*H*-quino[3,4-*b*][1,4]benzothiazinium chloride **6a** in DMSO$_{\text{-d6}}$

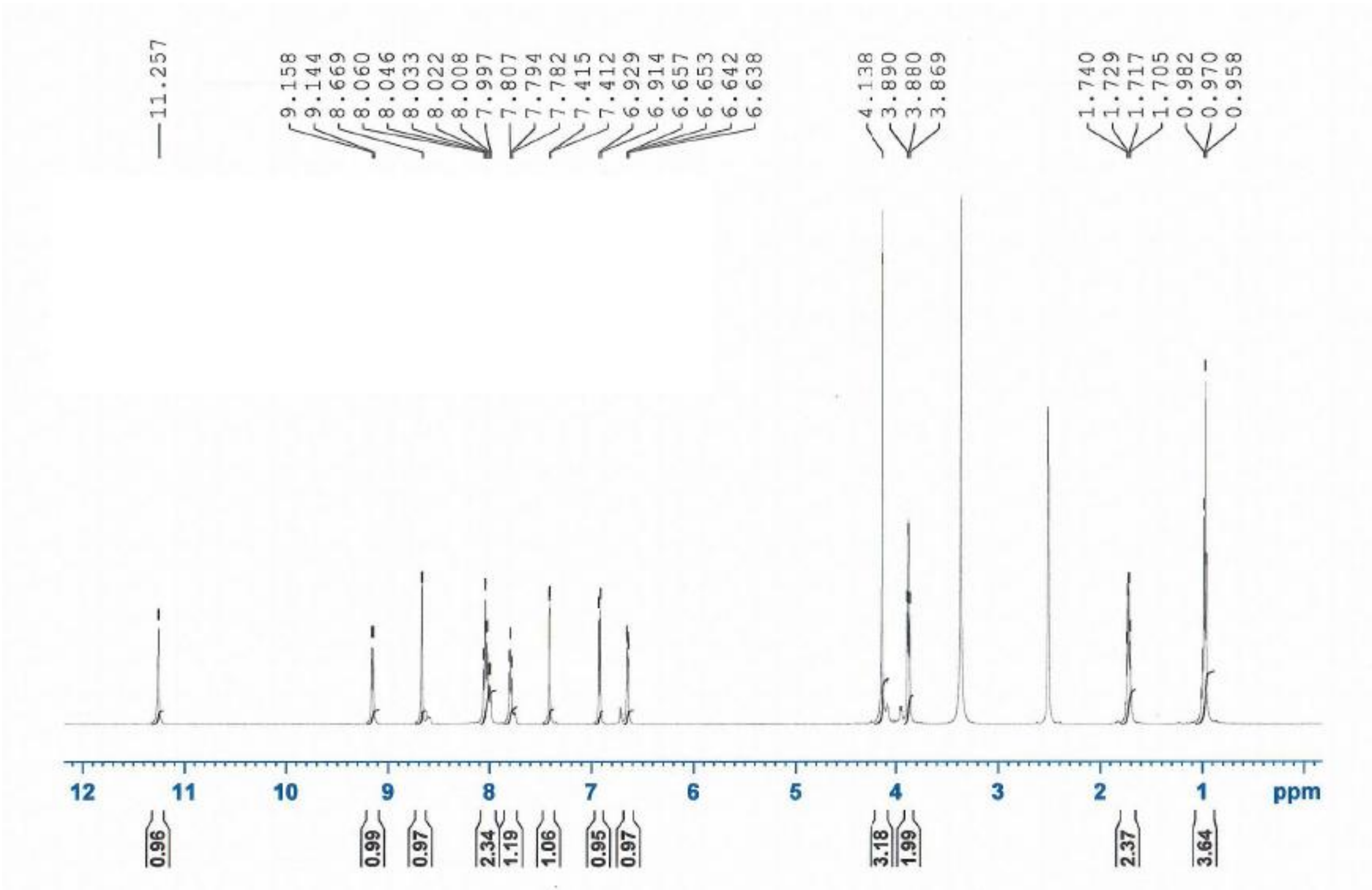


$^1$H NMR spectrum of 10-propoxy-5-methyl-12*H*-quino[3,4-*b*][1,4]benzothiazinium chloride **6b** in DMSO$_{\text{-d6}}$

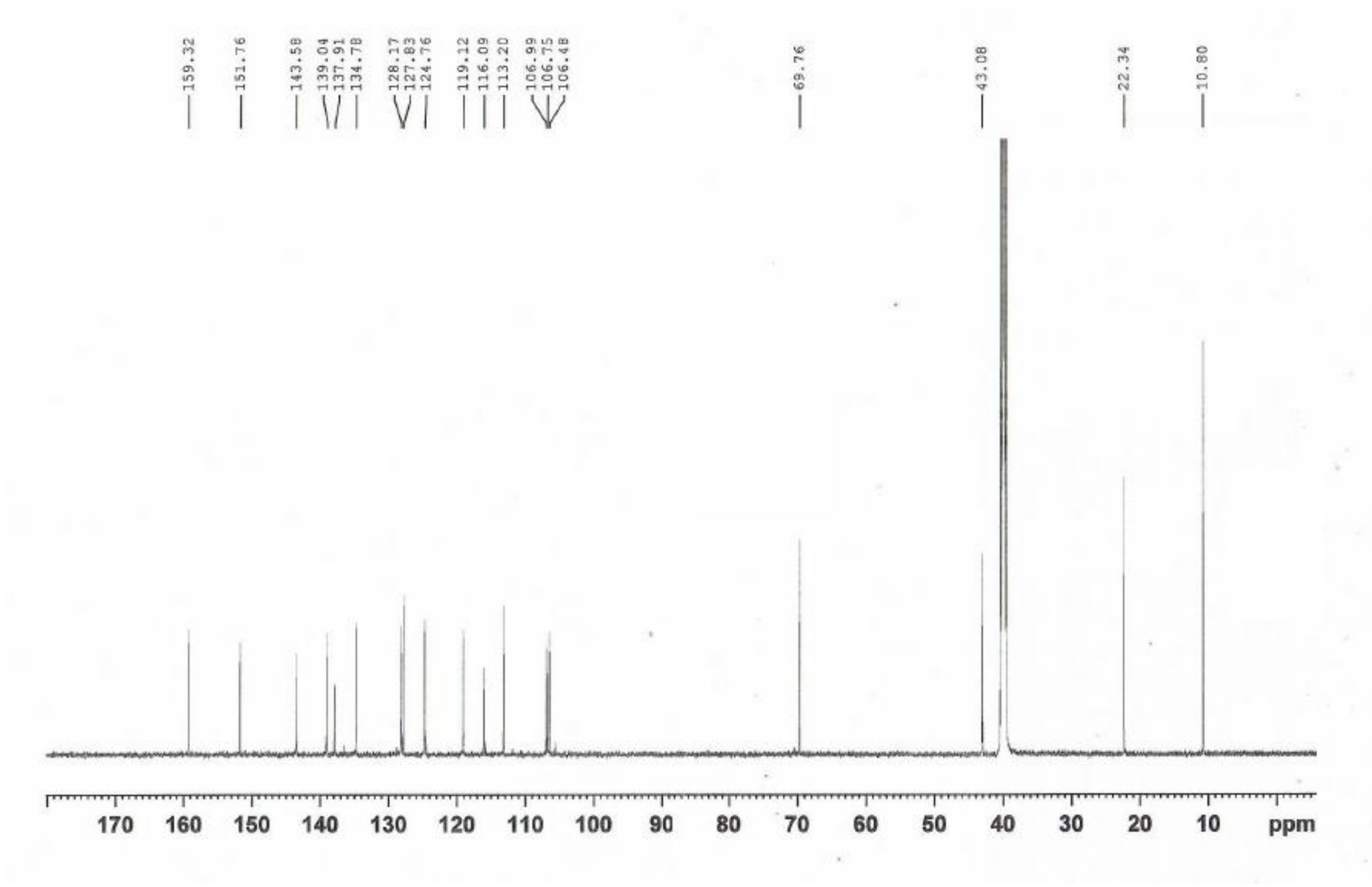


$^{13}$C NMR spectrum of 10-propoxy-5-methyl-12*H*-quino[3,4-*b*][1,4]benzothiazinium chloride **6b** in DMSO$_{-d6}$

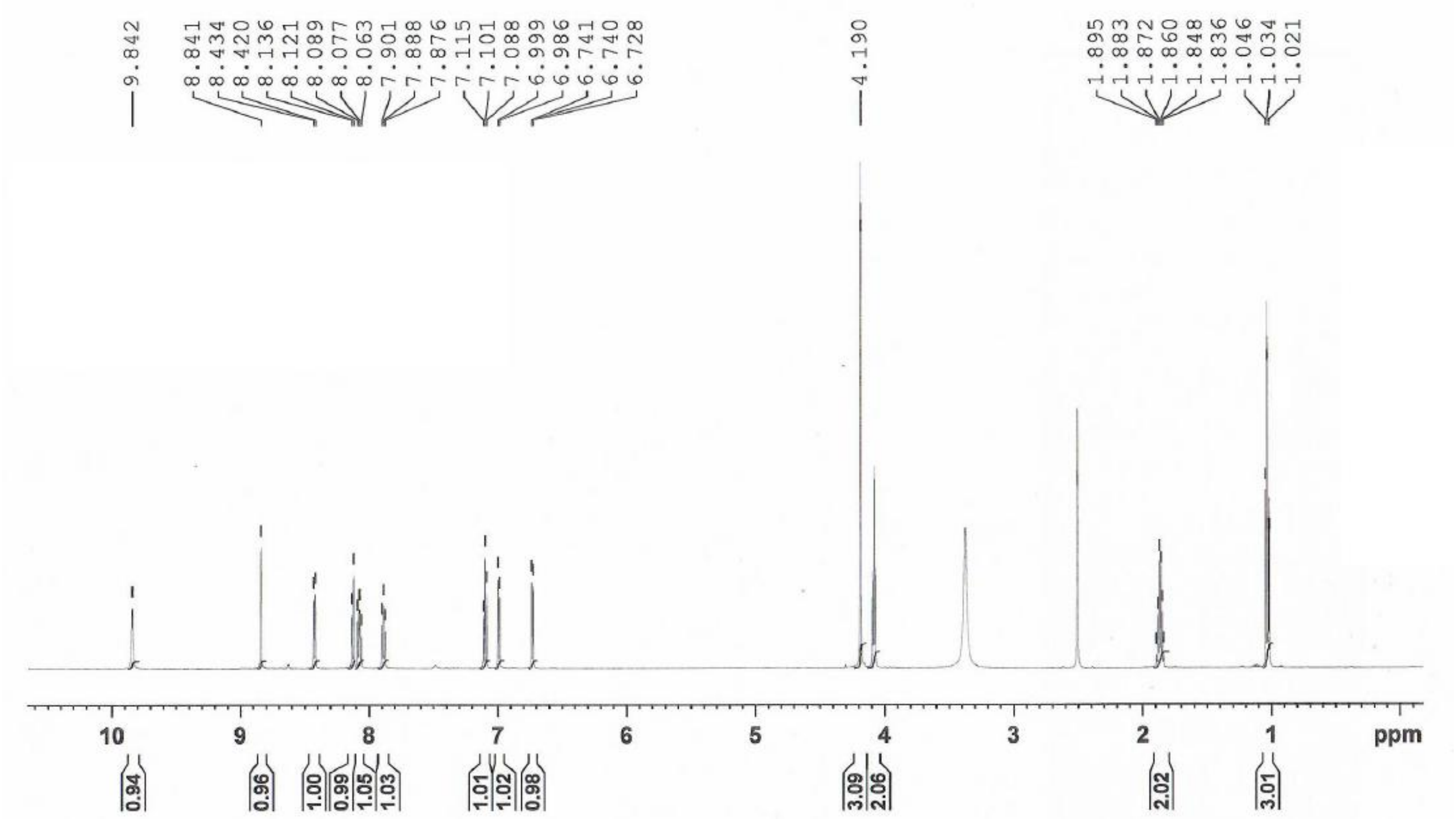

$^{1}$H NMR spectrum of 11-propoxy-5-methyl-12*H*-quino[3,4-*b*][1,4]benzothiazinium chloride **6c** in DMSO$_{\text{-d6}}$

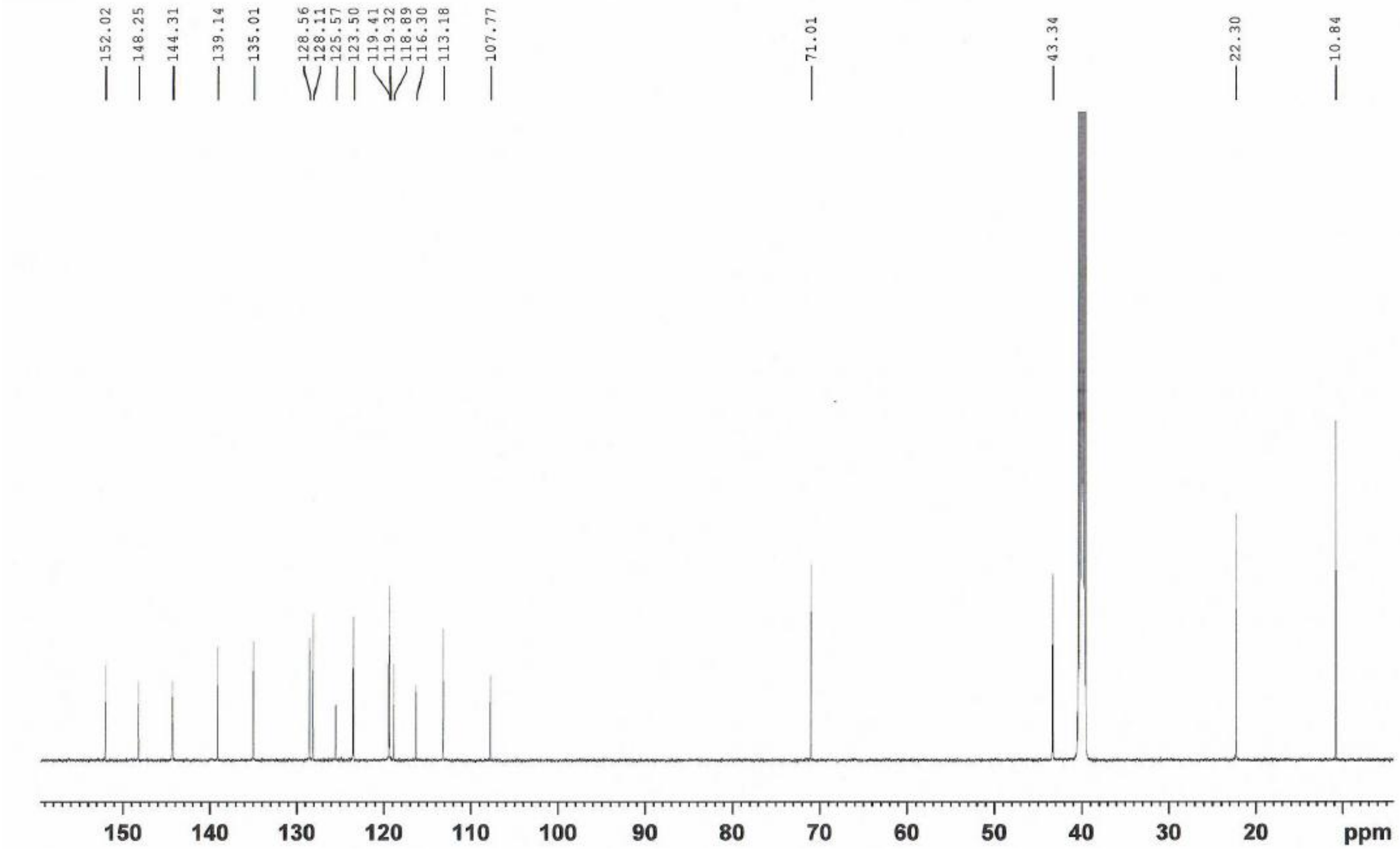


$^{13}C$ NMR spectrum of 11-propoxy-5-methyl-12*H*-quino[3,4-*b*][1,4]benzothiazinium chloride **6c** in $DMSO_{-d6}$

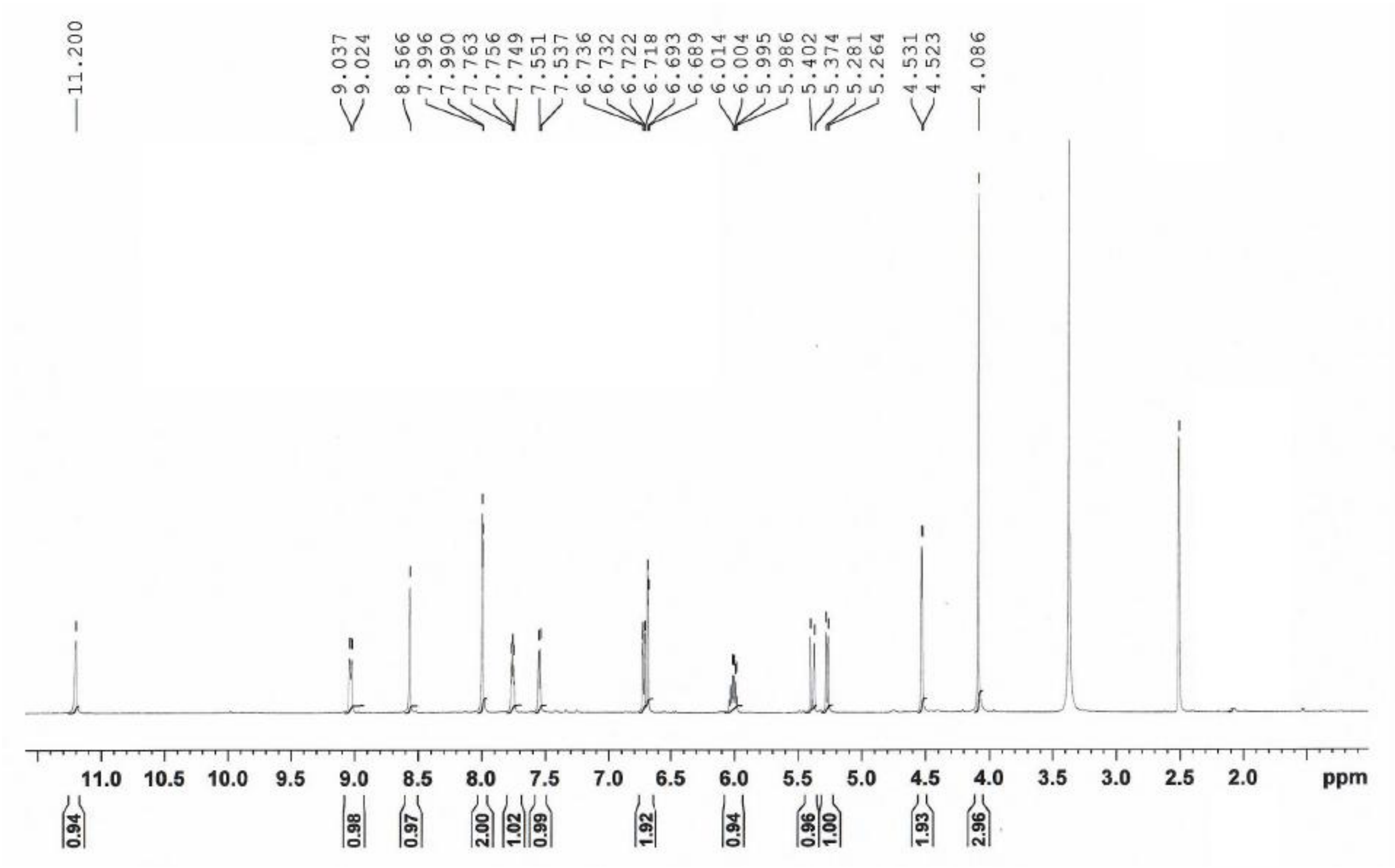


$^{1}$H NMR spectrum of 9-allyloxy-5-methyl-12*H*-quino[3,4-*b*][1,4]benzothiazinium chloride **6d** in DMSO$_{-d6}$

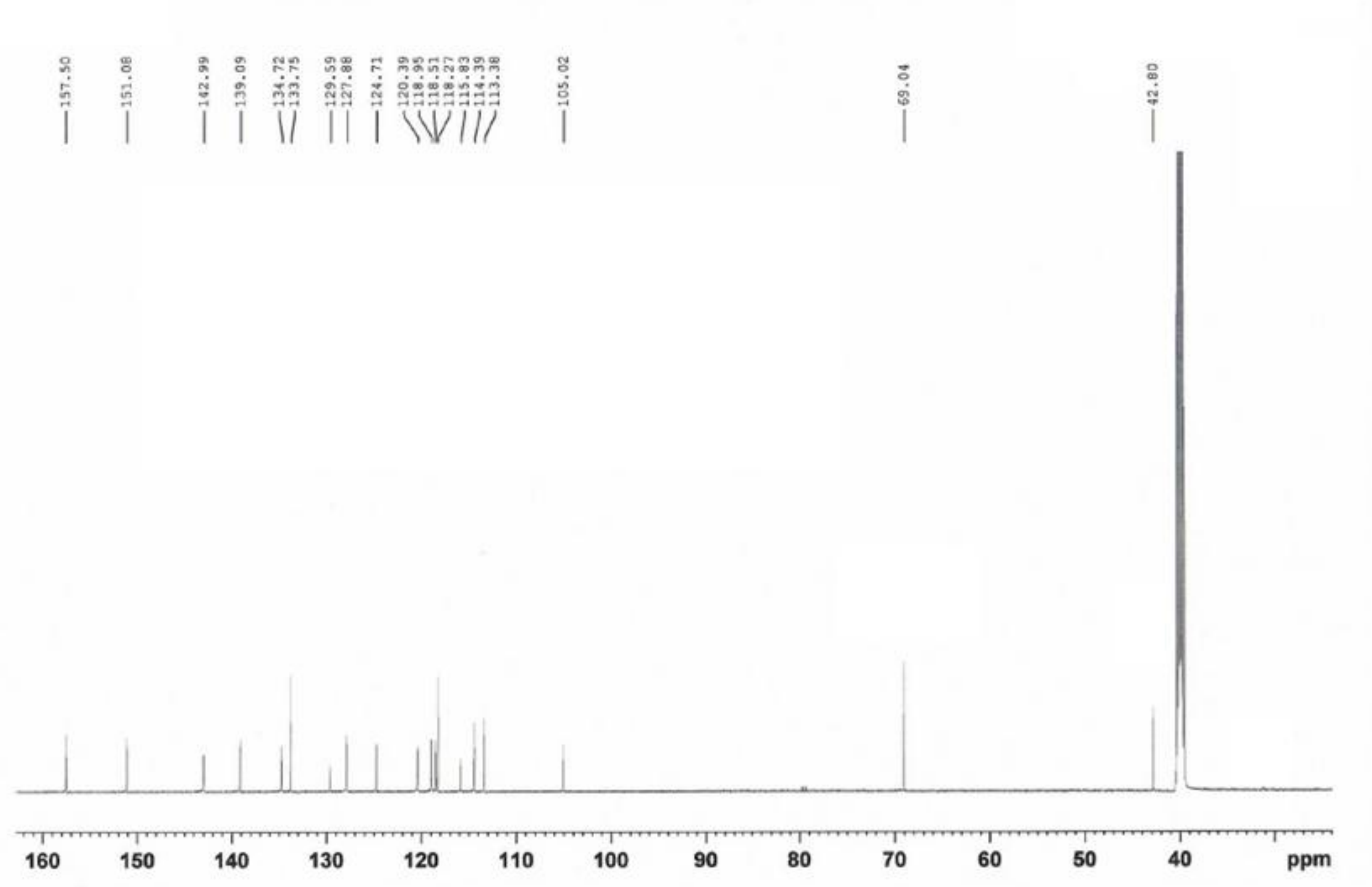


$^{13}C$ NMR spectrum of 9-allyloxy-5-methyl-12*H*-quino[3,4-*b*][1,4]benzothiazinium chloride **6d** in $DMSO_{-d6}$

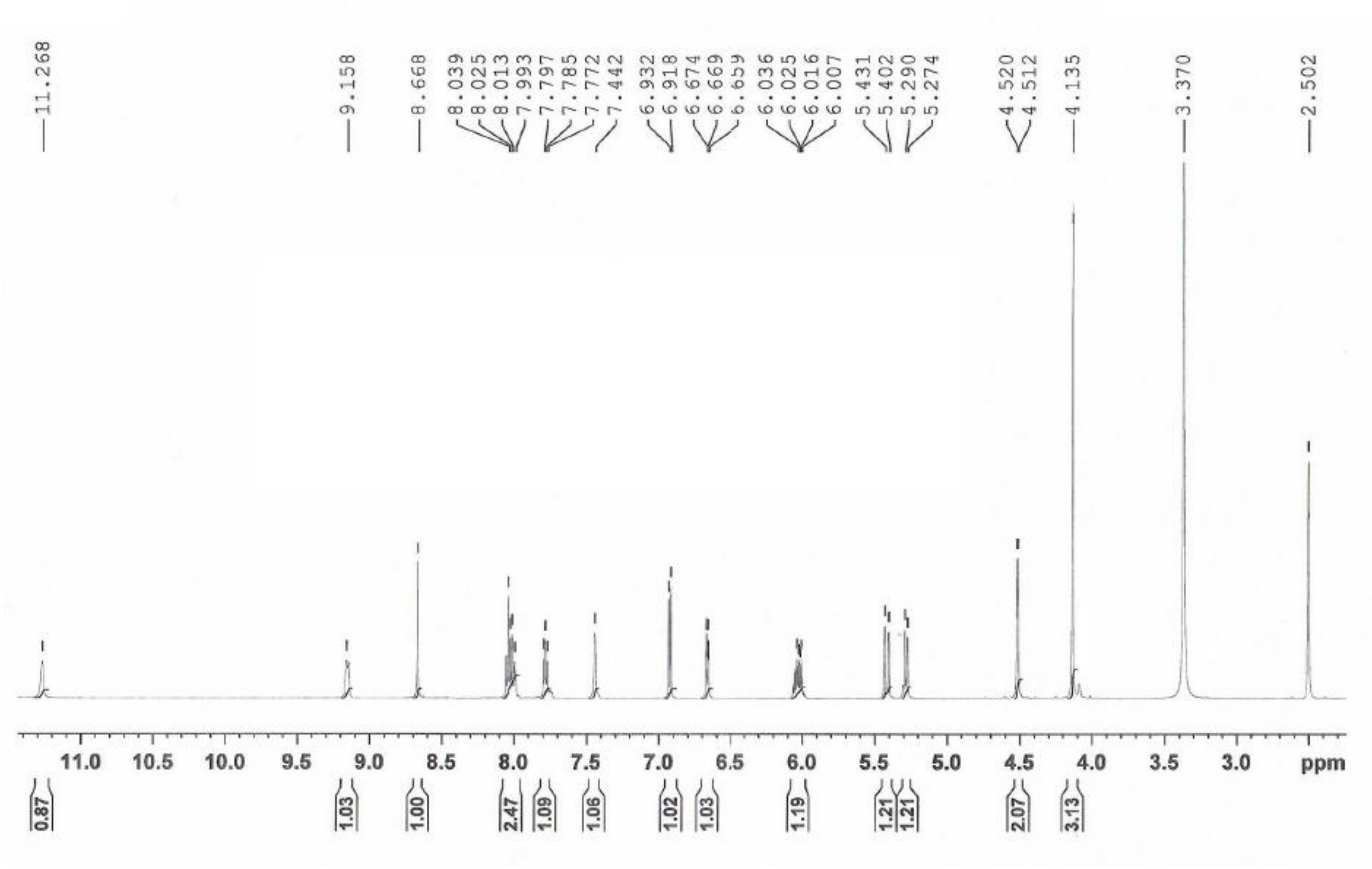


$^{1}$H NMR spectrum of 10-allyloxy-5-methyl-12*H*-quino[3,4-*b*][1,4]benzothiazinium chloride **6e** in $DMSO_{-d6}$

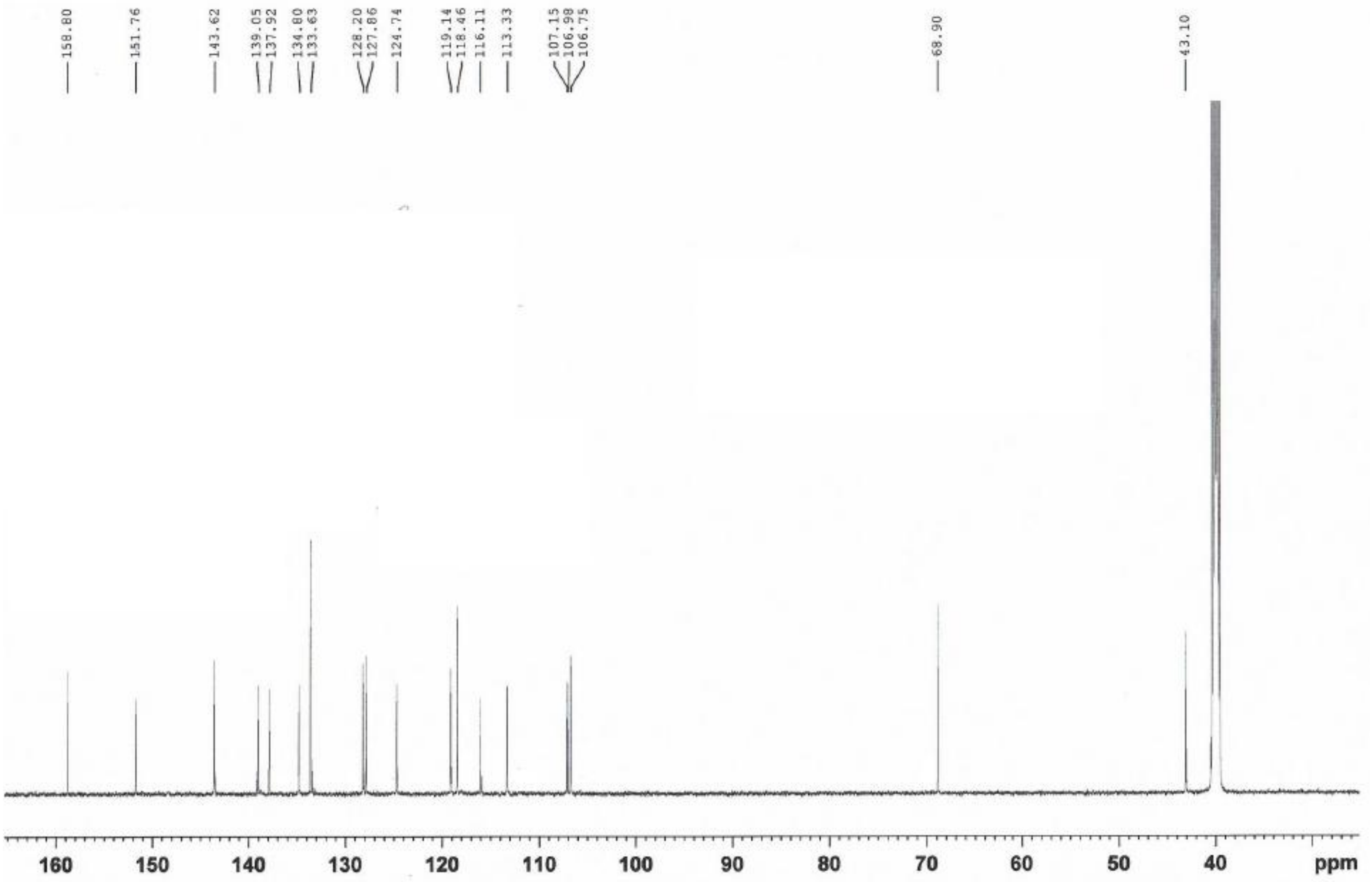


$^{13}C$ NMR spectrum of 10-allyloxy-5-methyl-12*H*-quino[3,4-*b*][1,4]benzothiazinium chloride **6e** in DMSO$_{-d6}$

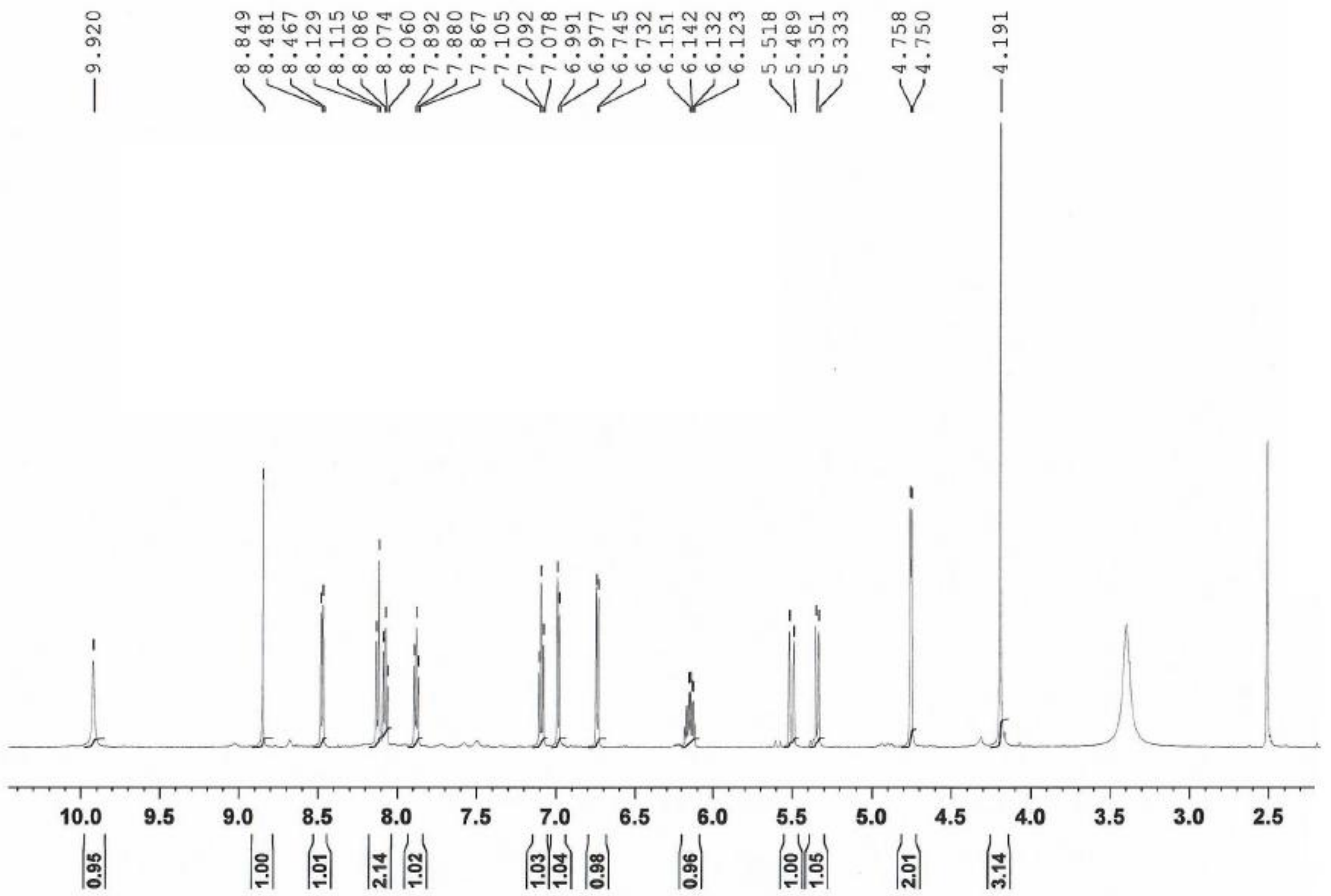


$^{1}$H NMR spectrum of 11-allyloxy-5-methyl-12*H*-quino[3,4-*b*][1,4]benzothiazinium chloride **6f** in DMSO$_{-d6}$

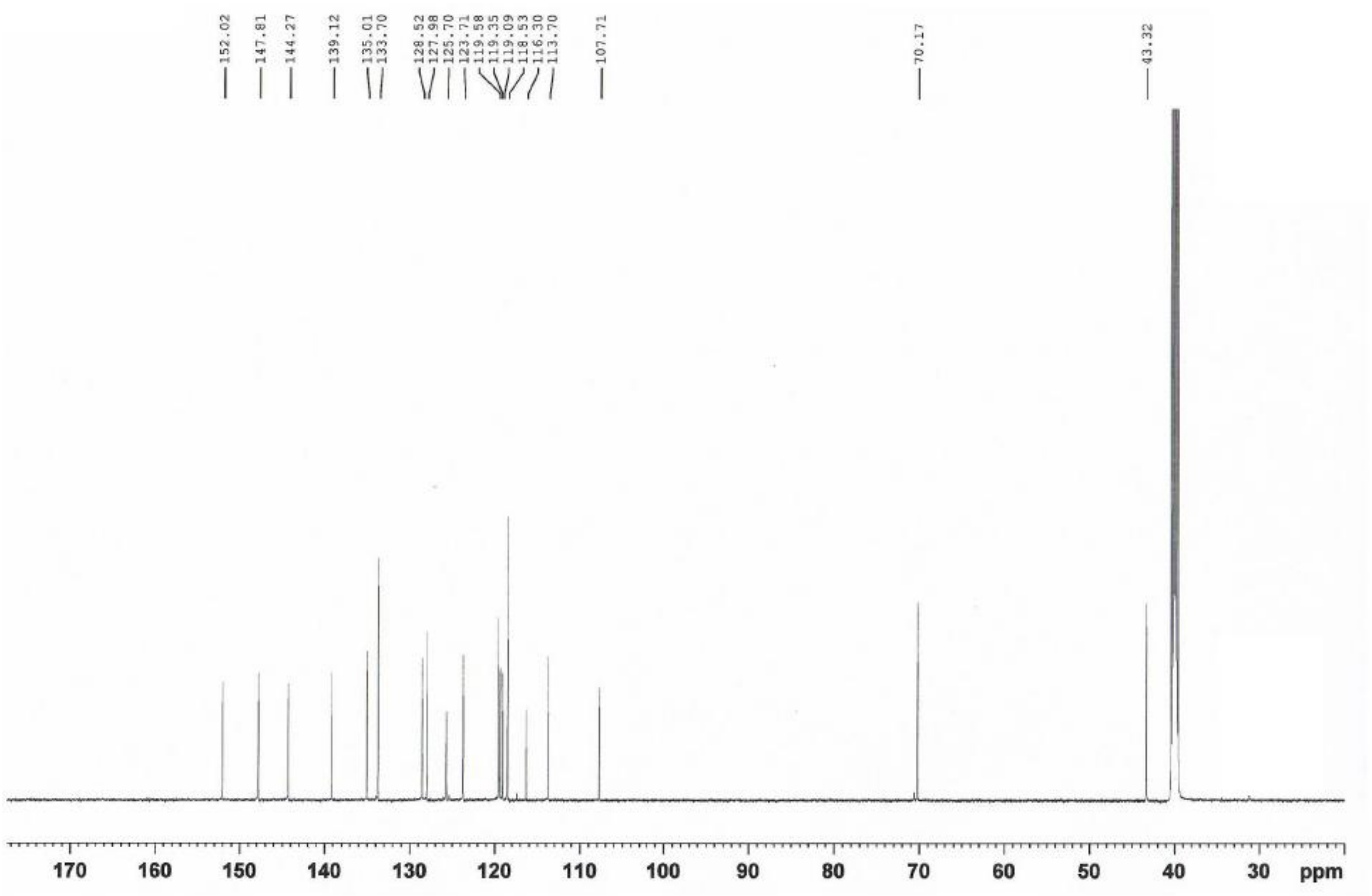


$^{13}$C NMR spectrum of 11-allyloxy-5-methyl-12*H*-quino[3,4-*b*][1,4]benzothiazinium chloride **6f** in $DMSO_{-d6}$

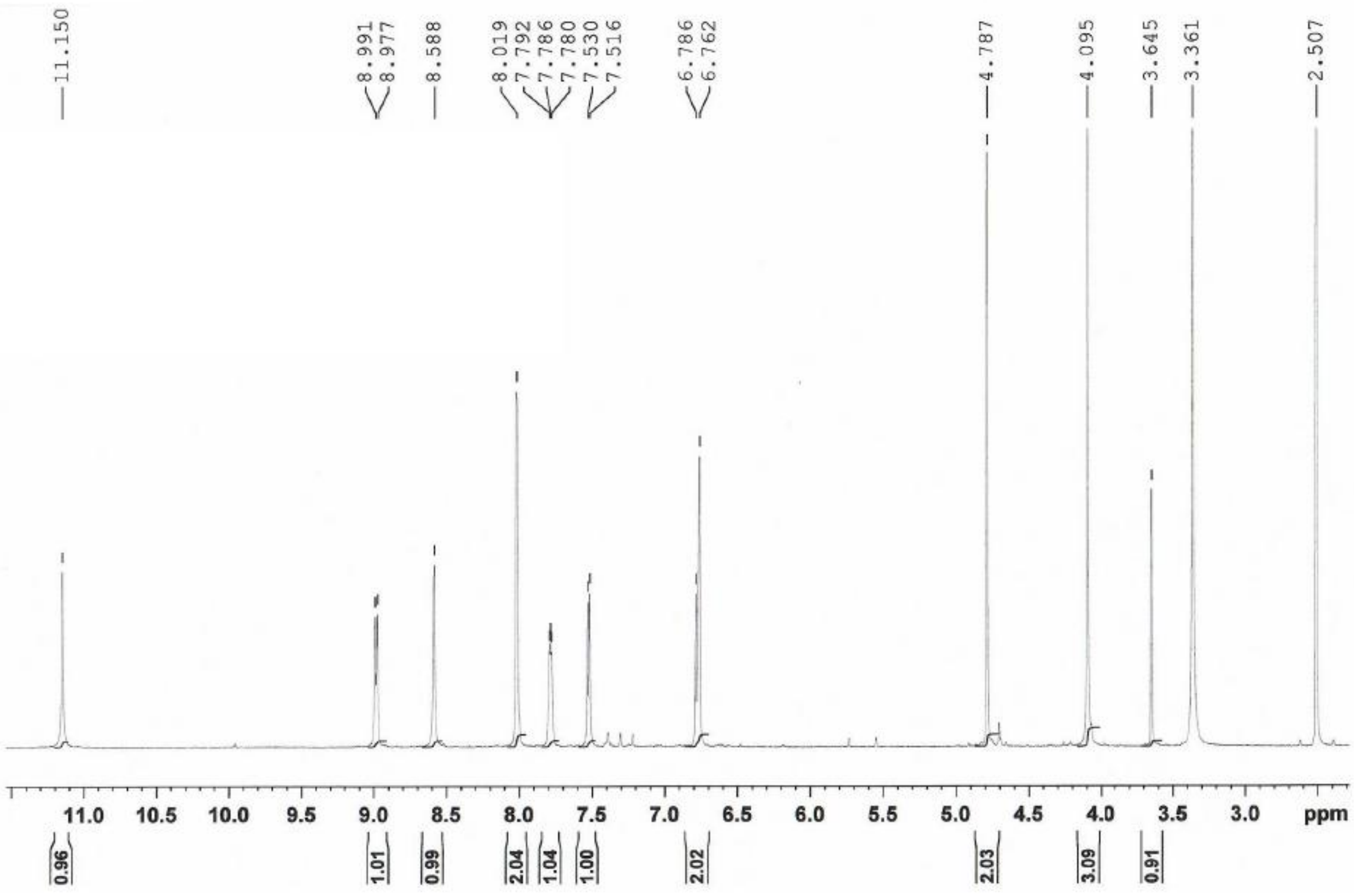


$^{1}$H NMR spectrum of 9-propargyloxy-5-methyl-12*H*-quino[3,4-*b*][1,4]benzothiazinium chloride **6g** in $DMSO_{-d6}$

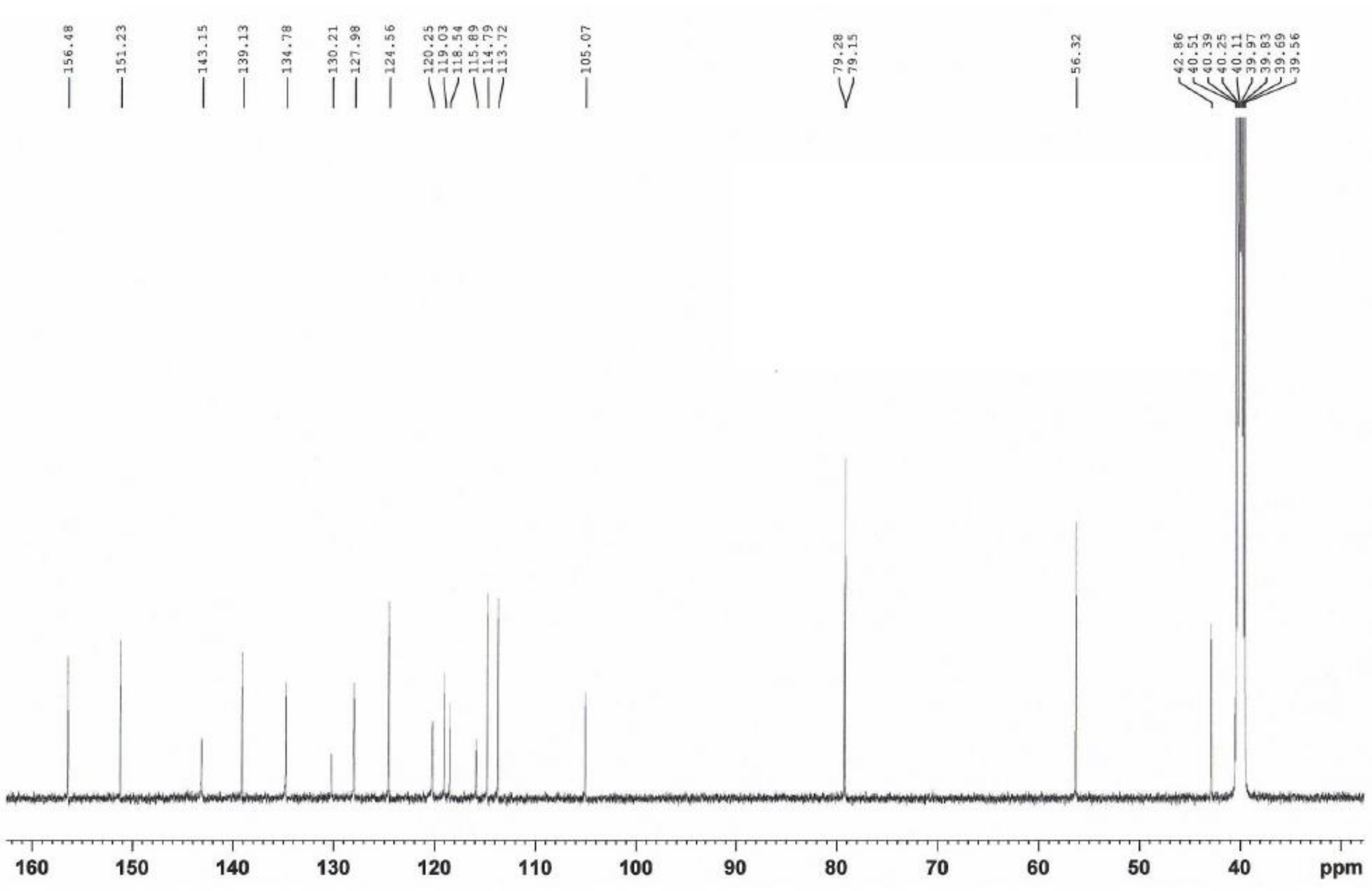


$^{13}$C NMR spectrum of 9-propargyloxy-5-methyl-12*H*-quino[3,4-*b*][1,4]benzothiazine chloride **6g** in DMSO$_{-d6}$.

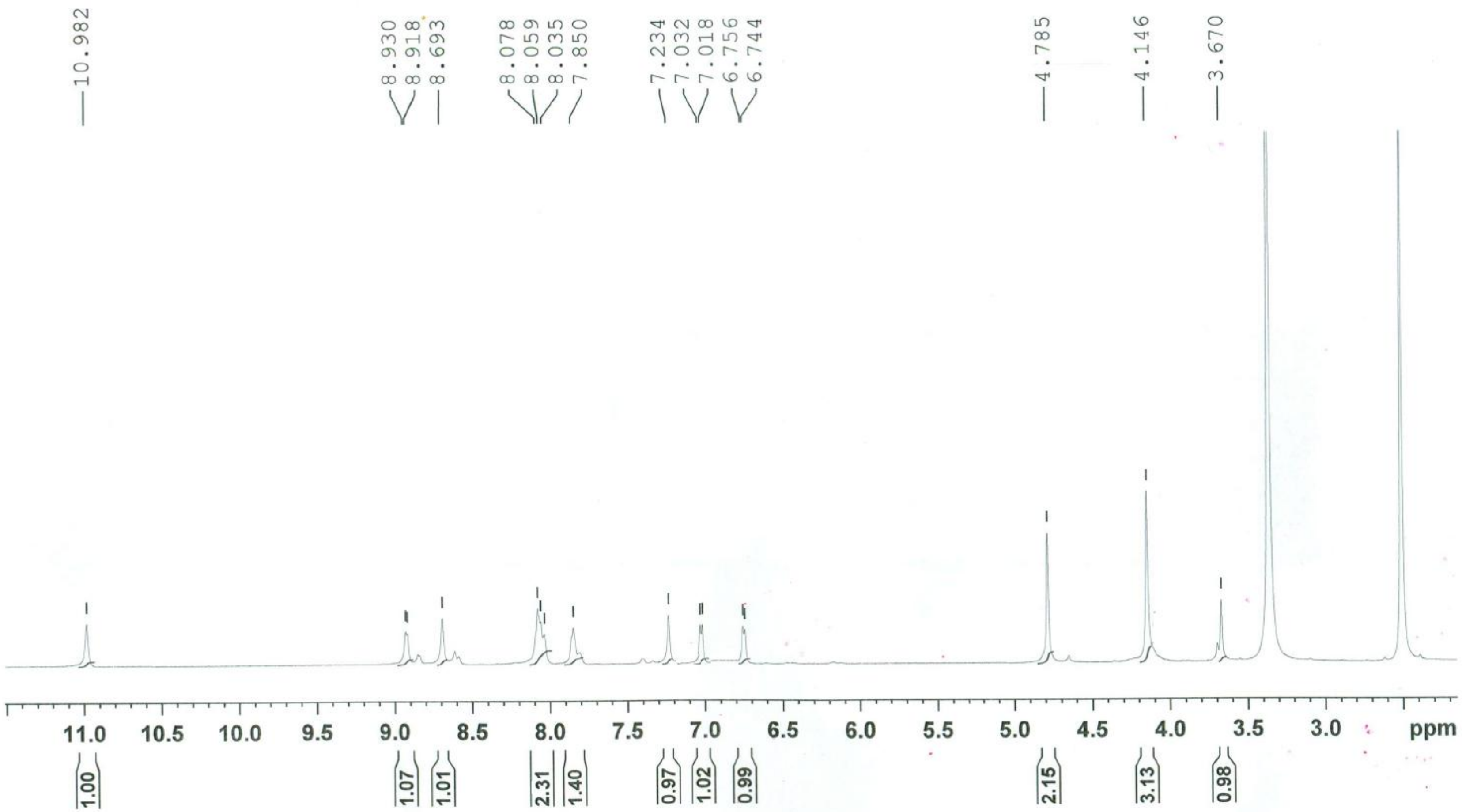


$^{1}$H NMR spectrum of 10-propargyloxy-5-methyl-12*H*-quino[3,4-*b*][1,4]benzothiazine chloride **6h** inDMSO$_{-d6}$

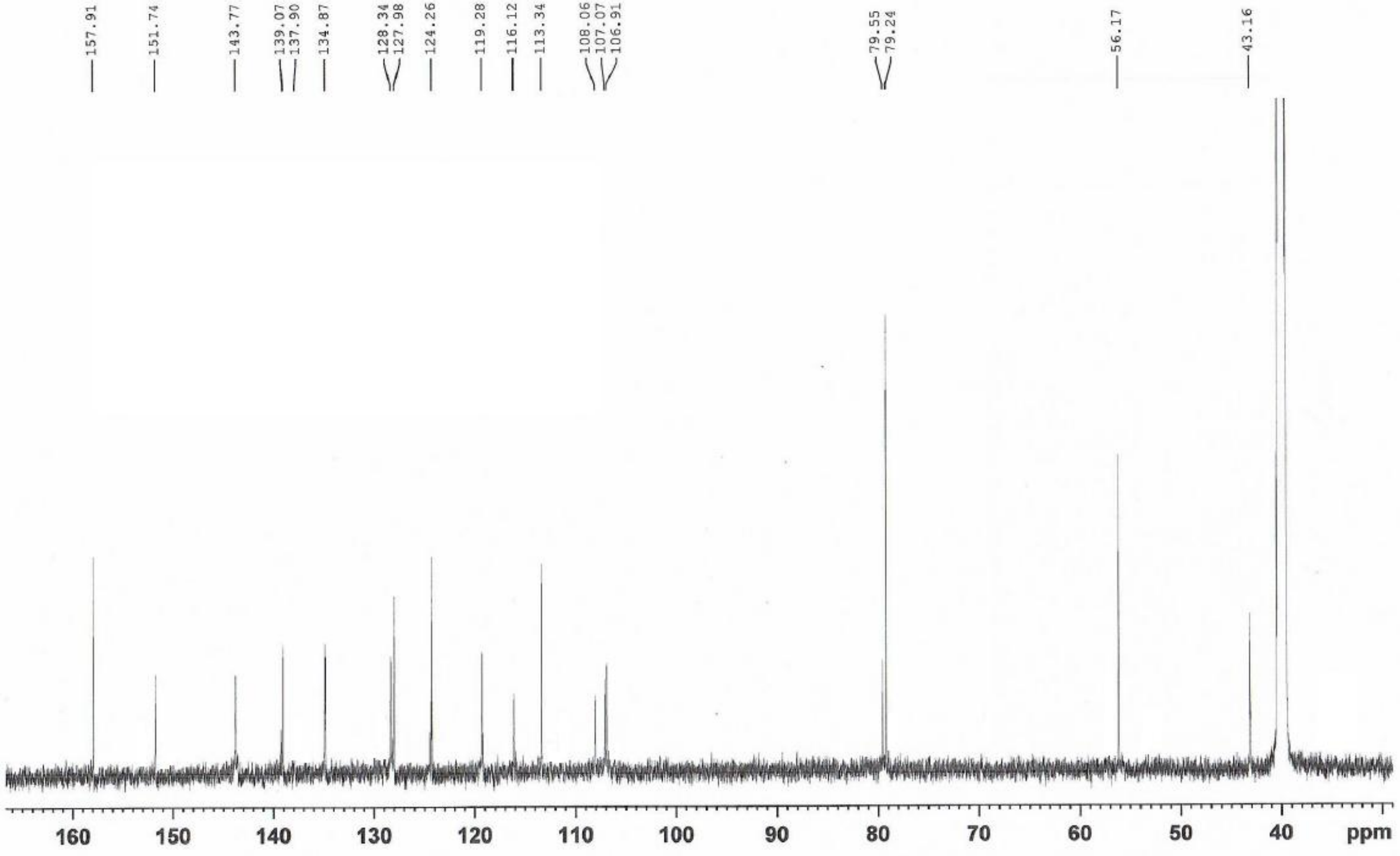


13C NMR spectrum of 10-propargyloxy-5-methyl-12*H*-quino[3,4-*b*][1,4]benzothiazinium chloride **6h** inDMSO$_{-d6}$

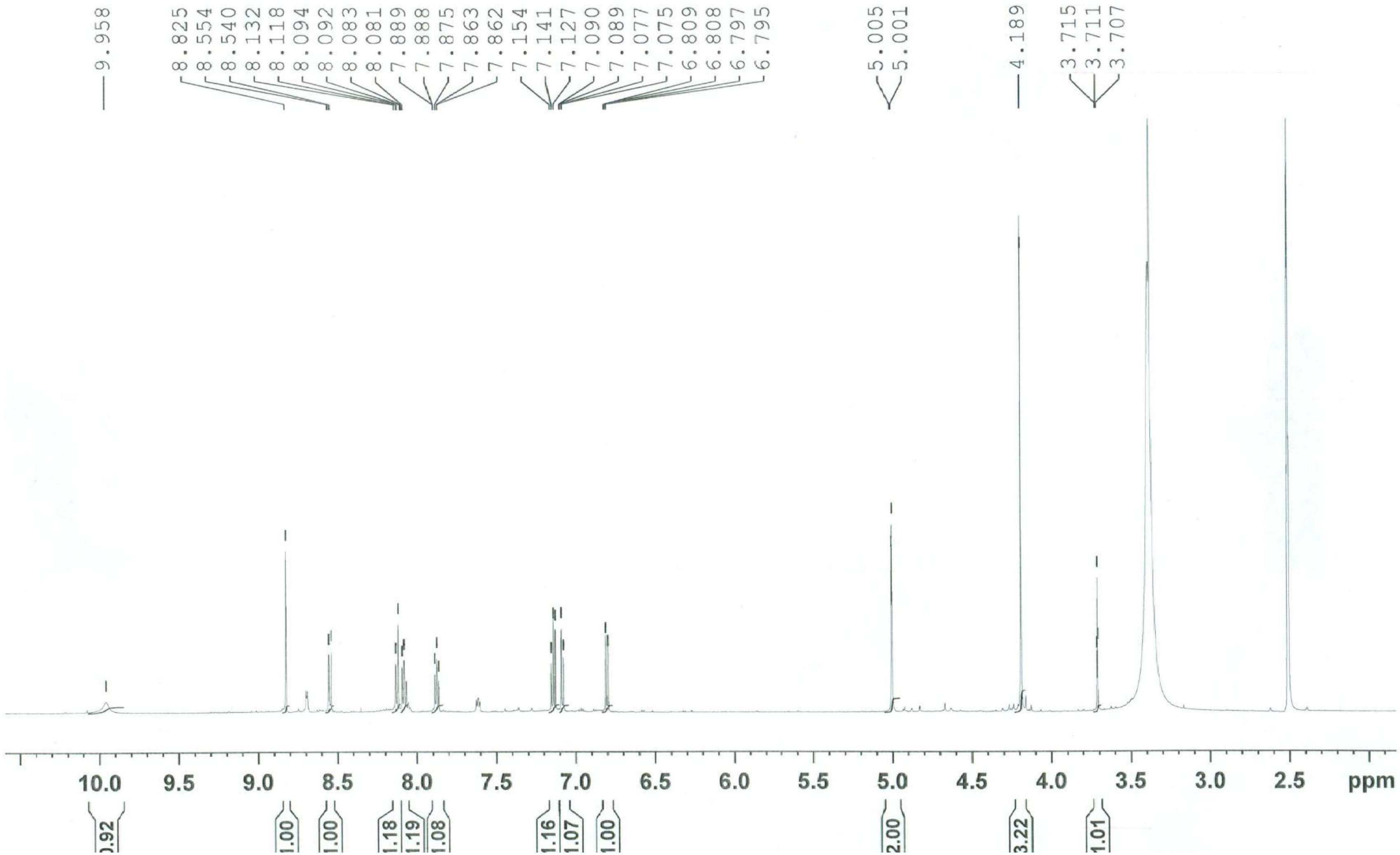


1H NMR spectrum of 11-propargyloxy-5-methyl-12*H*-quino[3,4-*b*][1,4]benzothiazinium chloride **6i** inDMSO$_{-d6}$

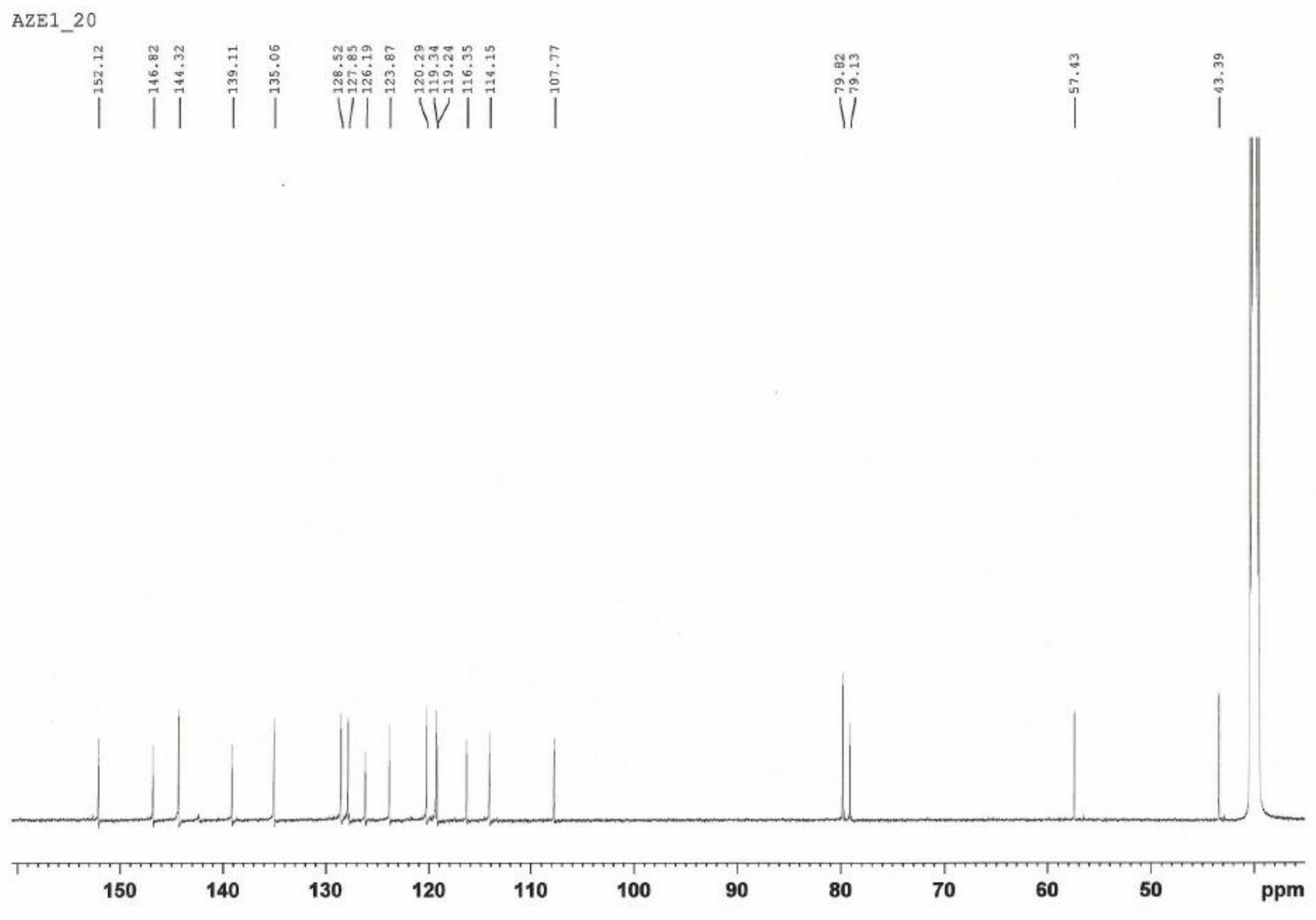


13C NMR spectrum of 11-propargyloxy-5-methyl-12*H*-quino[3,4-*b*][1,4]benzothiazinium chloride **6i** inDMSO$_{-d6}$

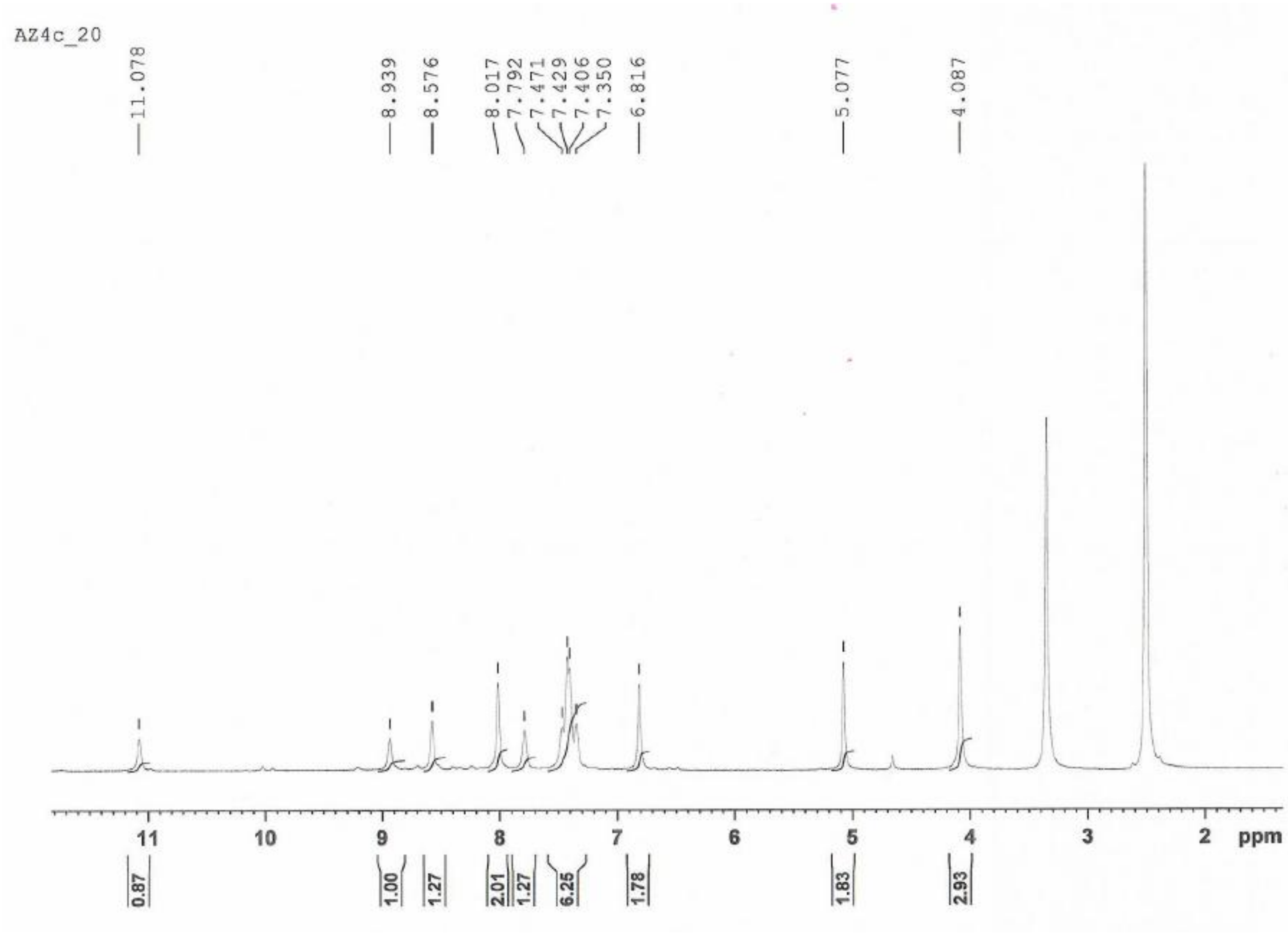


$^{1}$H NMR spectrum of 9-benzyloxy-5-methyl-12*H*-quino[3,4-*b*][1,4]benzothiazinium chloride **6j** in DMSO$_{-d6}$

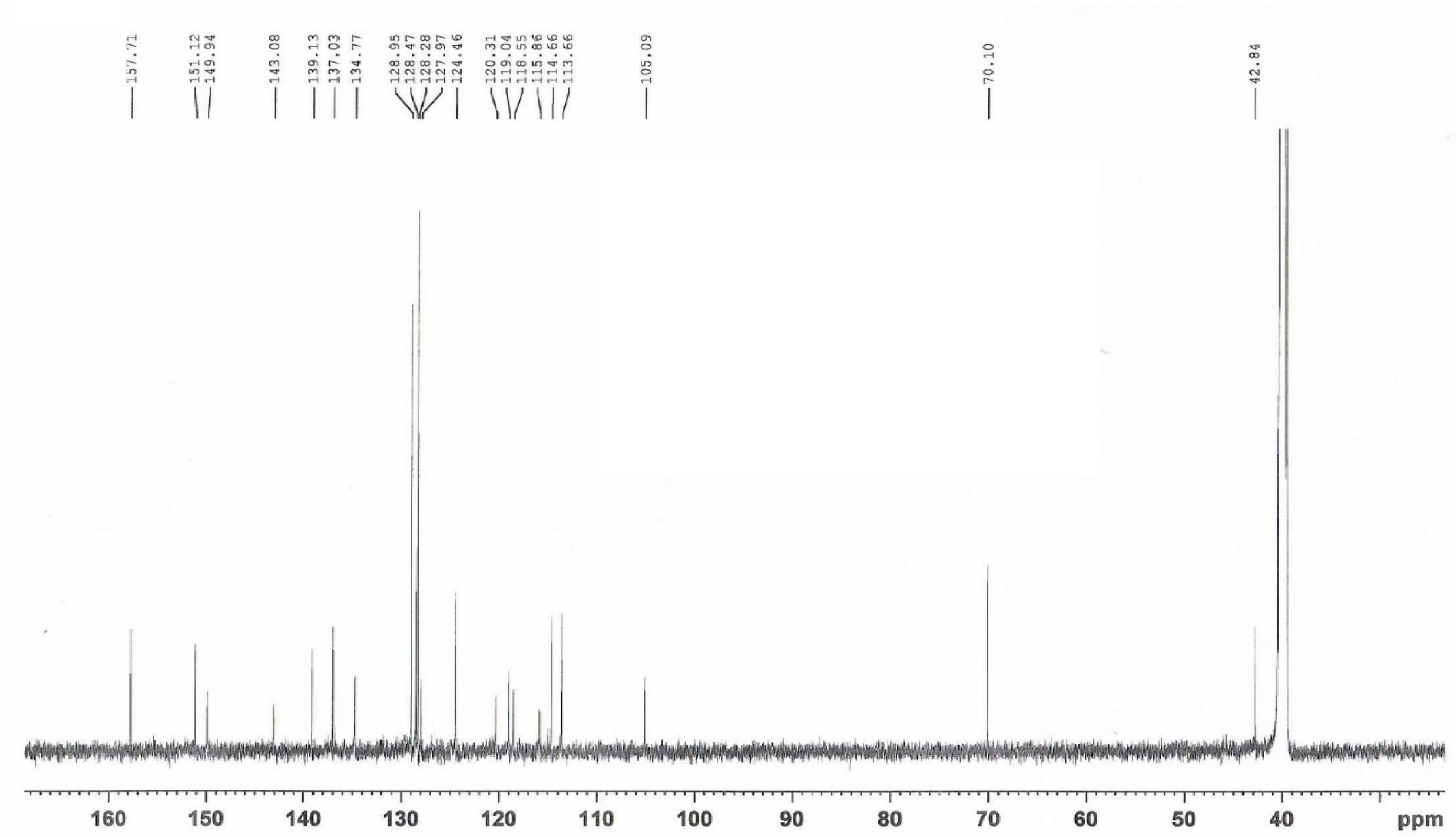


$^{13}$C NMR spectrum of9 -benzyloxy-5-methyl-12*H*-quino[3,4-*b*][1,4]benzothiazinium chloride **6j** in DMSO$_{\text{-d6}}$

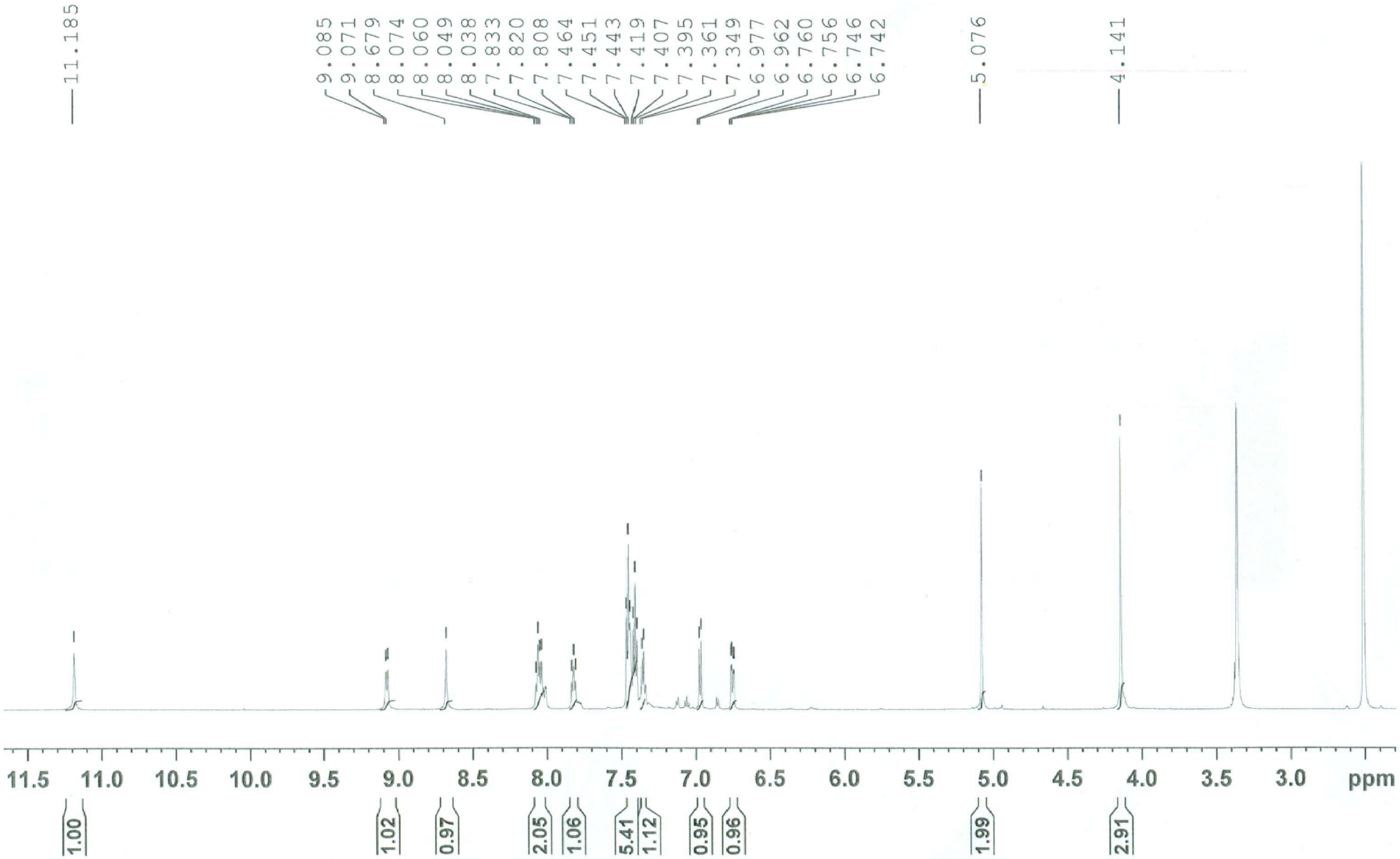


$^{1}$H NMR spectrum of 10-benzyloxy-5-methyl-12*H*-quino[3,4-*b*][1,4]benzothiazinium chloride **6k** in DMSO$_{-d6}$

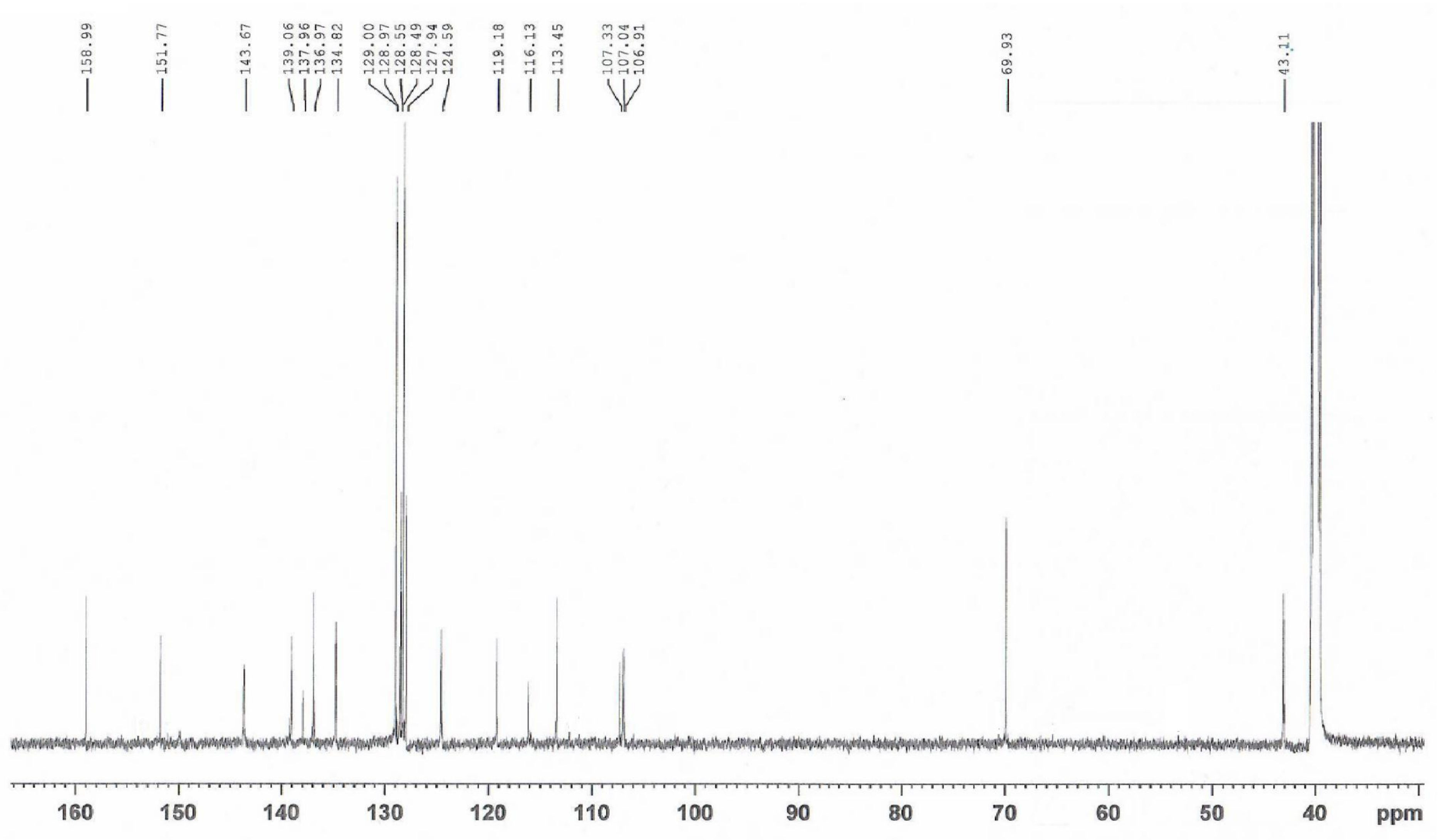


$^{13}$C NMR spectrum of 10-benzyloxy-5-methyl-12*H*-quino[3,4-*b*][1,4]benzothiazinium chloride **6k** in DMSO$_{-d6}$

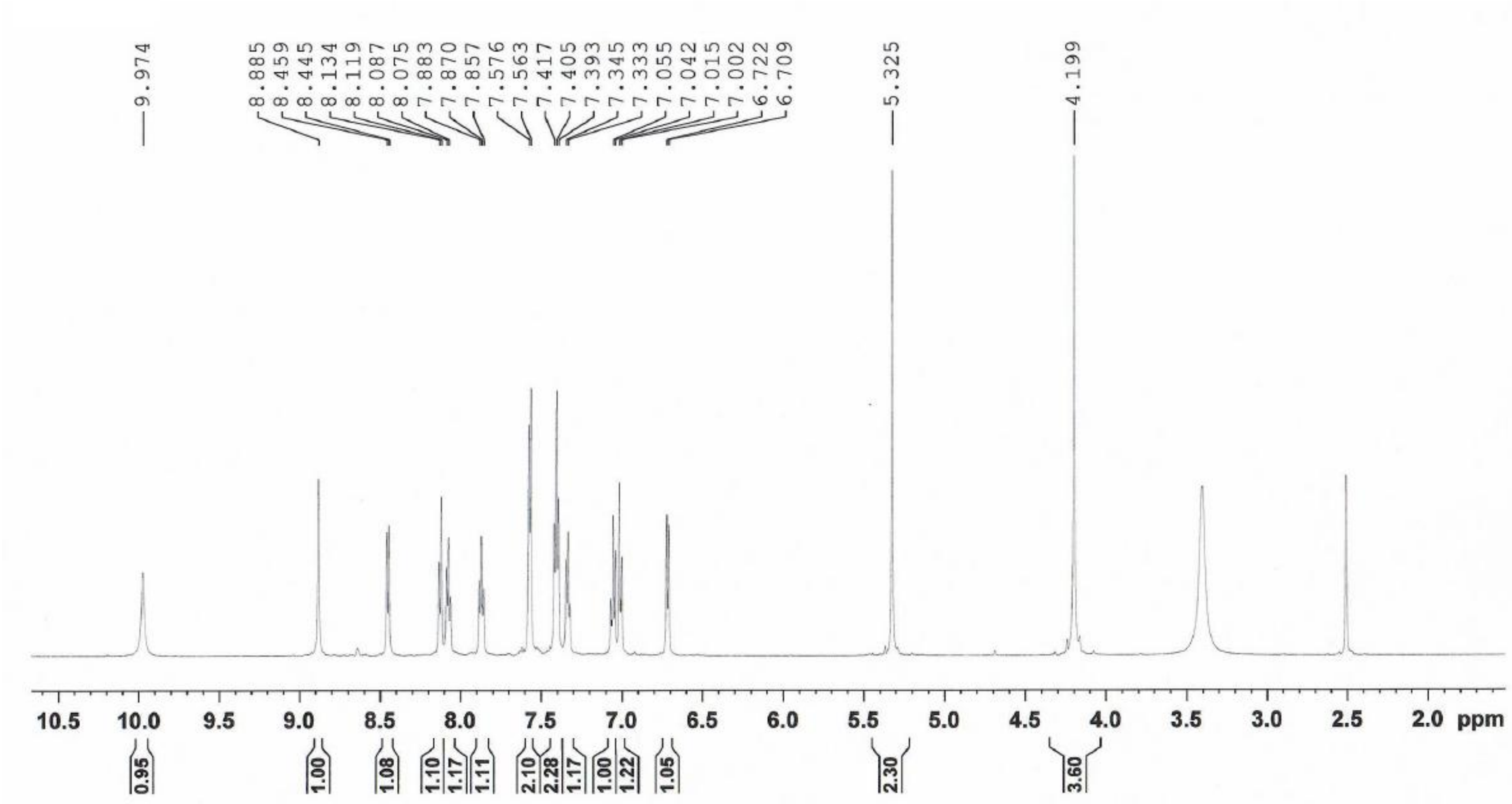


$^{1}$H NMR spectrum of 11-benzyloxy-5-methyl-12*H*-quino[3,4-*b*][1,4]benzothiazinium chloride **6l** in DMSO$_{\text{-d6}}$

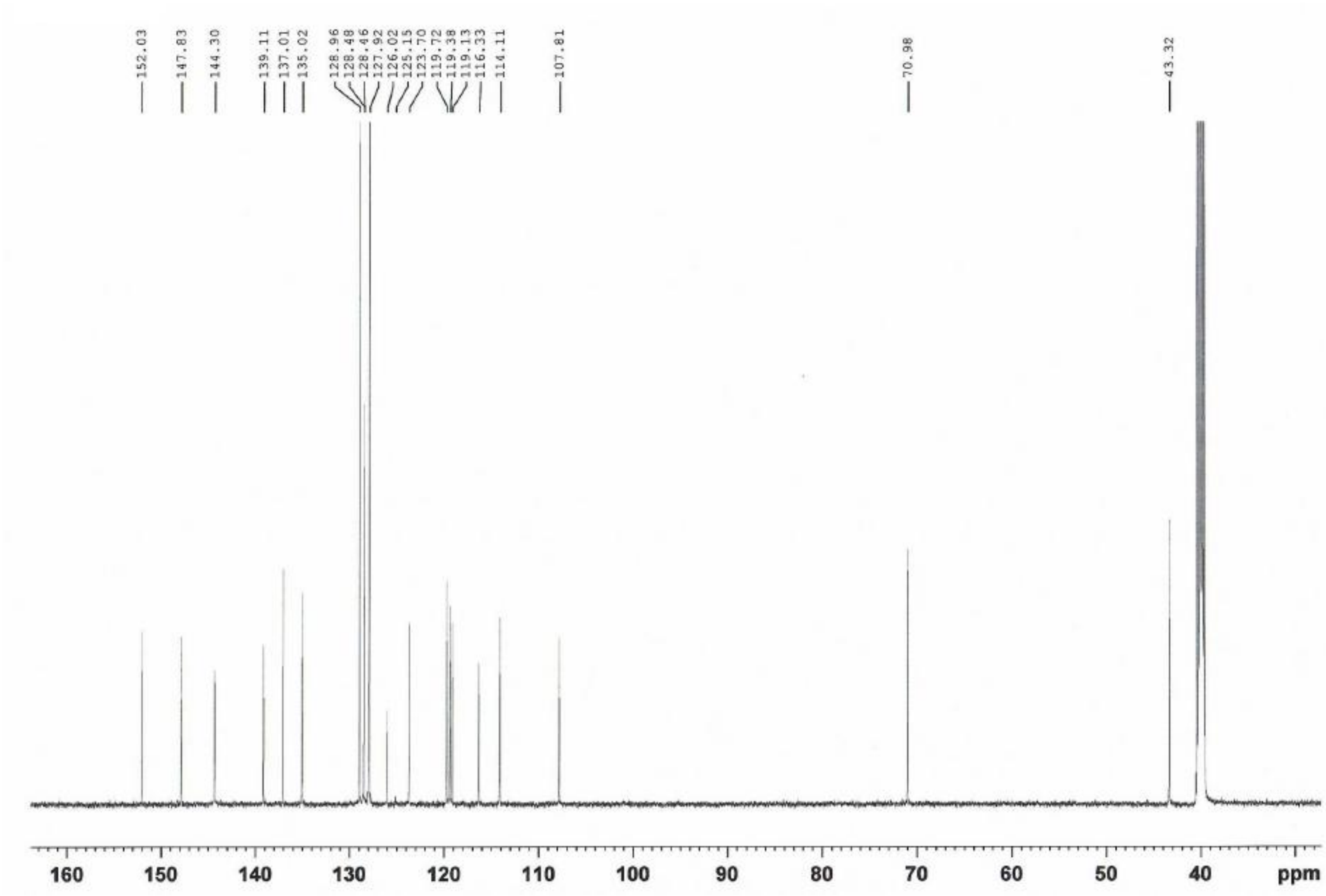


$^{13}$C NMR spectrum of 11-benzyloxy-5-methyl-12*H*-quino[3,4-*b*][1,4]benzothiazinium chloride **6l** in $DMSO_{-d6}$
